# Redefining Possible: Combining Phylogenomic and Supersparse Data in Frogs

**DOI:** 10.1093/molbev/msad109

**Published:** 2023-05-04

**Authors:** Daniel M Portik, Jeffrey W Streicher, David C Blackburn, Daniel S Moen, Carl R Hutter, John J Wiens

**Affiliations:** Department of Ecology and Evolutionary Biology, University of Arizona, Tucson, AZ; Department of Herpetology, California Academy of Sciences, USA; Department of Life Sciences, The Natural History Museum, London, United Kingdom; Department of Natural History, Florida Museum of Natural History, University of Florida, Gainesville, FL; Department of Integrative Biology, 501 Life Sciences West, Oklahoma State University, Stillwater, OK; Museum of Natural Science and Department of Biological Sciences, Louisiana State University, Baton Rouge, LA; Department of Ecology and Evolutionary Biology, University of Arizona, Tucson, AZ

**Keywords:** amphibians, missing data, phylogenomics, phylogeny, supermatrix

## Abstract

The data available for reconstructing molecular phylogenies have become wildly disparate. Phylogenomic studies can generate data for thousands of genetic markers for dozens of species, but for hundreds of other taxa, data may be available from only a few genes. Can these two types of data be integrated to combine the advantages of both, addressing the relationships of hundreds of species with thousands of genes? Here, we show that this is possible, using data from frogs. We generated a phylogenomic data set for 138 ingroup species and 3,784 nuclear markers (ultraconserved elements [UCEs]), including new UCE data from 70 species. We also assembled a supermatrix data set, including data from 97% of frog genera (441 total), with 1–307 genes per taxon. We then produced a combined phylogenomic–supermatrix data set (a “gigamatrix”) containing 441 ingroup taxa and 4,091 markers but with 86% missing data overall. Likelihood analysis of the gigamatrix yielded a generally well-supported tree among families, largely consistent with trees from the phylogenomic data alone. All terminal taxa were placed in the expected families, even though 42.5% of these taxa each had >99.5% missing data and 70.2% had >90% missing data. Our results show that missing data need not be an impediment to successfully combining very large phylogenomic and supermatrix data sets, and they open the door to new studies that simultaneously maximize sampling of genes and taxa.

## Introduction

Someday, we may have massive genome-scale data for most extant, described species on Earth with which to infer phylogenies (Lewin et al. 2018). But that day is clearly not yet here. Instead, we now have many species with molecular data from thousands of genes, many more species with data from a handful of genes (or none), and others with various numbers in between (e.g., Hinchliff et al. 2015; Lewin et al. 2018).

Given this situation, what is the best way to design molecular phylogenetic studies to deal with these gross disparities in the number of genes currently available for each species? At present, numerous studies use phylogenomic data sets, which typically contain limited numbers of species but hundreds of genes (e.g., Dunn et al. 2008; Prum et al. 2015; Hime et al. 2021) or thousands (e.g., Jarvis et al. 2014; Misof et al. 2014; Irisarri et al. 2017; Longo et al. 2017; Streicher et al. 2018, 2020). Other studies include fewer genes (often 20 or less) but can include thousands of species by incorporating data from GenBank (e.g., McMahon and Sanderson 2006; Pyron and Wiens 2011; Jetz et al. 2012; Pyron et al. 2013; Rainford et al. 2014; Jetz and Pyron 2018). These latter analyses are often referred to as “supermatrix” (Driskell et al. 2004; de Queiroz and Gatesy 2007) or “megaphylogeny” analyses (Smith et al. 2009). These well-sampled trees are crucial for large-scale evolutionary studies.

**Fig. 1.**
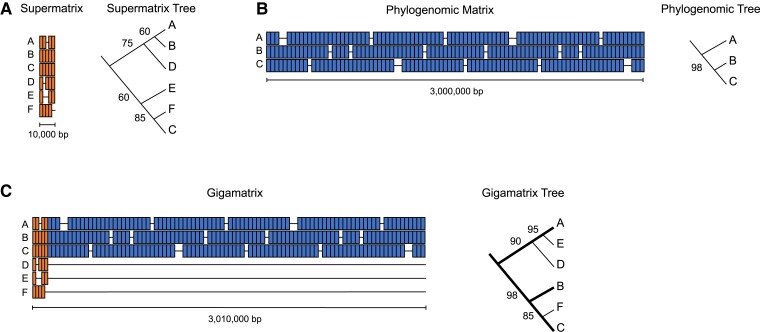
Cartoon examples illustrating the major types of analyses compared in this study. (A) The supermatrix approach includes many taxa and few genes, with some taxa missing data from one or more genes. (B) The phylogenomic matrix includes few taxa but many genes, again with some taxa missing data for one or more genes. (C) The gigamatrix combines the supermatrix and phylogenomic matrices and has many genes and taxa, but extensive missing data for those taxa not in the phylogenomic data set. Note that the numbers of genes shown here for each data set are much smaller than the actual numbers used in our study (i.e., ∼300 for the supermatrix vs. ∼4,000 for the phylogenomic and gigamatrix data sets). A simplified hypothetical tree generated from each matrix is also shown, with bootstrap support values at each node. Here, the gigamatrix tree is fully resolved and has higher mean support values than the supermatrix tree alone. Moreover, conflicts between the supermatrix and phylogenomic trees (regarding the placement of taxon B) are resolved in favor of the phylogenomic tree in the gigamatrix tree. Many other outcomes might be possible, however. For example, the gigamatrix tree might have weaker mean branch support than the trees from the separate analyses, and the conflict between the supermatrix and phylogenomic trees over the position of taxon B might be resolved in favor of the supermatrix tree instead.

Is it possible to combine phylogenomic and supermatrix approaches? For example, can we make a single, massive “gigamatrix” (fig. 1) that contains thousands of markers and hundreds (or thousands) of species? Should we? One can imagine pros and cons to such an approach (table 1). On the positive side, the phylogenomic data could allow for better resolution of higher-level relationships (given the increased number of informative sites), whereas the supermatrix data would allow for inclusion of hundreds of species (fig. 1). Hypothetically, this combination should give a better estimate of higher-level relationships than a pure supermatrix approach (based on many fewer genes), whereas estimates for species-level relationships might be no worse.

**Table 1.** Potential Advantages and Disadvantages of the Gigamatrix Approach, and Whether They Are Supported by Our Results or Not.

Potential Advantage	Supported Here?
Higher-level relationships resemble those from phylogenomic tree	Yes
Many species can be accurately placed based on supermatrix data alone	Yes
Stronger support for higher-level relationships than from supermatrix	Yes
Potential disadvantage	
Many highly incomplete taxa misplaced by extensive missing data	No
Higher-level relationships resemble those from supermatrix tree	No
Higher-level relationships identical to phylogenomic tree	No
Weak support for higher-level tree because of highly incomplete taxa	Usually no
Difficult to use species-tree methods on gigamatrix	Yes
Difficult to assemble gigamatrix	Mostly no
Computationally intensive (difficult to simultaneously analyze thousands of genes and thousands of taxa)	Yes

Of course, this is an optimistic view. There are several potential negatives (table 1). Most importantly, the data matrix from the combined phylogenomic supermatrix (gigamatrix) analysis would be dominated by missing data (i.e., unknown or ambiguous data cells). For example, each taxon in the supermatrix (represented by few genes) would have missing data for the thousands of genes in the phylogenomic data set (fig. 1). Presumably, these highly incomplete taxa with few genes would greatly outnumber those with phylogenomic data, leading to a new matrix that is supersparse (i.e., most taxa with >90% missing data). But whether this massive amount of missing data would be problematic is less clear. The impact of missing data on phylogenetic analysis has been an ongoing topic of theoretical and empirical research and debate (e.g., Wiens 2003; Philippe et al. 2004; Lemmon et al. 2009; Cho et al. 2011; Sanderson et al. 2010, 2011, 2015; Wiens and Morrill 2011; Roure et al. 2013; Jiang et al. 2014; Hosner et al. 2016; Streicher et al. 2016; Xi et al. 2016; Nute et al. 2018; Talavera et al. 2022). Although the specific impacts of missing data may depend on the phylogenetic method and other details, some studies suggest that missing data cells are not intrinsically problematic, and excluding taxa and/or characters to avoid missing data may be problematic instead (e.g., Jiang et al. 2014; Streicher et al. 2016).

Moreover, there are many other unresolved questions (table 1). First, would the higher-level relationships from the gigamatrix tree reflect those from the phylogenomic analysis (making the combined analysis worthwhile), or would they have a greater resemblance to those from the supermatrix (making the phylogenomic data superfluous)?

Second, would support values for higher-level relationships in the gigamatrix tree reflect the phylogenomic analyses (fig. 1) or the supermatrix analysis? A terminal taxon lacking phylogenomic data might be weakly resolved in its placement simply because it has data for few genes. More importantly, a highly incomplete taxon with little data to resolve its placement might lead to weak support over much of the tree, even with data for thousands of genes in the other terminal taxa. On the positive side, including phylogenomic data might help resolve the placement of those taxa with data for only a few genes. Alternatively, the support for placing highly incomplete taxa might be limited by their overall lack of data.

Third, the combination of phylogenomic and supermatrix data sets might preclude the use of species-tree methods to estimate relationships. Thus, gigamatrix analysis may require use of concatenated analyses. Yet, it remains unclear whether species-tree methods presently give better estimates than concatenated analyses. For example, Portik and Wiens (2021) found that concatenated analyses recovered a higher proportion of well-established relationships (i.e., corroborated by both molecular and morphological data) than did species-tree analyses.

Finally, the combination of supermatrix and phylogenomic data sets by itself presents major bioinformatic challenges. Obtaining sequences for thousands of species and markers from public databases (e.g., NCBI and GenBank) is not trivial. Bioinformatic methods for creating supermatrices fall into two general categories. First, some methods perform automated sequence clustering to find usable markers. These include PhyLoTA (Sanderson et al. 2008), SUPERSMART (Antonelli et al. 2017), phylotaR (Bennett et al. 2018), and PyPHLAWD (Smith and Walker 2018). Second, some methods perform targeted searches for specific markers, including phyloGenerator (Pearse and Purvis 2013) and SuperCRUNCH (Portik and Wiens 2020). Targeted searches may be especially useful for building customized matrices (e.g., combining supermatrix and phylogenomic data sets). Furthermore, many phylogenomic data sets are not on NCBI but instead are only available as Supplementary Materials online or in other repositories (e.g., Dryad and FigShare). Therefore, phylogenomic data can be incompatible with methods that rely exclusively on GenBank (e.g., phyloGenerator, PhyLoTA, phylotaR, PyPHLAWD, and SUPERSMART). This incompatibility issue also applies to newly generated sequences, making inclusion of unpublished sequences difficult.

Given these issues, what is known about the combination of phylogenomic and supermatrix data sets? Few studies have addressed this topic. For example, one study (Zheng and Wiens 2016) performed such a combined analysis in squamate reptiles (lizards and snakes). However, their “phylogenomic” data set was very limited: only 44 genes for 161 species. These data were combined with a supermatrix of 12 genes for 4,161 species. The higher-level relationships from the combined analysis generally reflected those from the 44-gene data set more than those from the 12-gene data set. Mean bootstrap support for higher-level nodes in the combined data tree was marginally higher than in the analyses of the 12-gene data set. Similarly, a study in butterflies by Talavera et al. (2022) showed that adding a backbone data set of nine genes (in 8% of the taxa) to a single-gene DNA-barcoding data set (92% of taxa) yielded combined trees in which higher-level relationships more closely matched the backbone data set than the barcoding data alone. Those authors also used simulations to show that trees from combined data sets were more accurate than trees from barcodes alone and could be highly accurate when the added backbone data set included only 5–50% of the taxa. Although these results are promising, it is unclear what would happen if the phylogenomic data set contained thousands of markers instead of dozens.

Here, we test the effects of combining phylogenomic and supermatrix approaches in frogs. First, we assembled a phylogenomic data set of 155 species (138 frogs and 17 outgroups), including new data from 70 species. This data set consisted of sequences from UCEs (Bejerano et al. 2004; Faircloth et al. 2012) and contained 3,784 nuclear markers. Second, we assembled a supermatrix of 307 markers representing most frog genera, including 441 ingroup taxa (of 456 genera; AmphibiaWeb 2020). These included markers used in large-scale supermatrices (Pyron and Wiens 2011; Jetz and Pyron 2018) and large-scale multilocus data sets (Feng et al. 2017; Hime et al. 2021). We focused on including most genera, given that initial matrices containing all these markers and ∼5,000 species proved to be computationally intractable. Third, we combined the UCE data set and supermatrix into a single gigamatrix, with 4,091 markers and 441 ingroup taxa. We used SuperCRUNCH (Portik and Wiens 2020) to address the bioinformatic challenges of generating a new supermatrix and combining this matrix with published (and unpublished) phylogenomic data sets.

We used the trees from these three matrices to address three key questions about the gigamatrix approach. First, does the large amount of missing data in the combined phylogenomic-supermatrix analysis cause problematic results, given that 86% of the 1.5 billion cells in this gigamatrix are empty and >40% of the taxa each have >99.5% missing data cells? The most obvious problem would be that some taxa are clearly misplaced on the tree, relative to their placement in previous molecular and morphological studies. Second, do higher-level relationships from the gigamatrix analysis (441 taxa) share more higher-level nodes with the phylogenomic (UCE) analysis or with the supermatrix analysis? If relationships are most similar to those in the supermatrix analysis, it would suggest that the potential power of the phylogenomic data to help resolve those nodes is overwhelmed by the many supersparse taxa in the supermatrix data set. Third, do the phylogenomic (UCE) data increase support for higher-level relationships in the gigamatrix tree, relative to the supermatrix alone? Or is branch support limited by the instability of the most incomplete terminal taxa? In addition to our analyses of frogs, we also compare the answers to these questions to those from a gigamatrix study in squamates with fewer markers (Zheng and Wiens 2016). Overall, we find that the gigamatrix approach yields reasonable placements of highly incomplete taxa (i.e., consistent with previous studies), higher-level relationships that are similar to the phylogenomic tree, and increased branch support relative to the supermatrix approach alone.

Finally, we note that despite recent studies on frog phylogeny, many aspects of anuran relationships remain uncertain. Recent studies have generally agreed regarding the base of frog phylogeny, but there has been conflict over relationships within Neobatrachia. Neobatrachia contains most frog species and families (∼95% of species and >80% of families; AmphibiaWeb 2020). Results are most disparate between a supermatrix study based on 15 mitochondrial (mt) and nuclear genes (Jetz and Pyron 2018) and phylogenomic studies using many nuclear markers (Feng et al. 2017; Streicher et al. 2018; Hime et al. 2021). Furthermore, these phylogenomic studies used a limited number of markers (<250; Feng et al. 2017; Hime et al. 2021) or used many markers (>2,000) but focused on only one clade (Hyloidea; Streicher et al. 2018). Here, we present the first large-scale phylogenomic analysis that spans most frog families and includes thousands of markers, along with a gigamatrix analysis that includes all families and most genera. We also include a time-calibrated version of the gigamatrix tree for use in comparative studies.

## Results

### Data Matrices

Basic properties of the data sets are given in table 2 and supplementary material S1 and figure S1, Supplementary Material online. The UCE data set (table 2) contained 3,784 markers and 155 species (138 ingroup and 17 outgroup; supplementary material S2, Supplementary Material online). The concatenated alignment was 2,935,116 base pairs (bp) in length, with 63.2% missing data cells. The average number of UCEs per frog species was 1,887 (SD [standard deviation] = 675; range = 42–2,880; supplementary material S2, Supplementary Material online). For new UCE data generated here, the mean was 2,157 UCEs (*n* =70 species; range = 460–2,880; supplementary material S2, Supplementary Material online). UCE sequences and alignments are provided on an Open Science Framework project page: https://osf.io/fzw3x/. The supermatrix contained 452 taxa, 307 markers and was 27,611 bp in length (with 70.4% missing data; details in supplementary materials S3 and S4, Supplementary Material online).

**Table 2.** Characteristics of the Concatenated Data Matrices Analyzed Here.

Marker Sets	Rank	Taxa	Markers	Sequences	Length (bp)	Info. sites (%)	Missing Data (%)
UCEs	Species	155	3,784	302,047	2,935,116	1,449,947 (49.4)	63.2
UCEs	Genus	137	3,784	275,290	2,935,116	1,411,791 (48.1)	62.3
Supermatrix	Genus	452	307	51,798	27,611	13,198 (47.8)	70.4
Gigamatrix	Genus	458	4,091	327,088	3,298,631	1,606,433 (48.7)	86.0

Length is the number of base pairs (bp) in each concatenated alignment. The percentage of sites that are parsimony-informative is given in “Info. Sites.” See also supplementary material S1 and figure S1, Supplementary Material online.

The gigamatrix (combined UCE + supermatrix; table 2) contained 4,091 markers and 458 taxa (441 ingroup genera, 17 outgroup species). The alignment was 3,298,631 bp long and contained 1,510,772,998 cells and 86.0% missing data (tables 2 and 3). We note that most of the missing data were from markers being entirely absent in some taxa, but there were also some missing data that came from within some markers (e.g., due to gaps in alignment).

**Table 3.** Characteristics of Different Marker Types in the Gigamatrix.

Marker Set	Markers	Taxa	Length (bp)	Info. Sites (%)	Missing Data (%)
AHE	194	189	1,290	54.8	2.9
NPCL	89	116	961	48.9	3.2
Legacy	24	194	1,150	47.0	24.8
UCE	3,784	72	775	48.4	29.3

Number of taxa, length, informative (info.) sites, and missing data are all means per marker in each marker set. AHE refers to anchored hybrid enrichment markers of Hime et al. (2021). NPCL refers to PCR-based nuclear protein-coding loci of Feng et al. (2017). Legacy refers to 16 nuclear markers (*BDNF*, *BMP2*, *CMYC*, *CXCR4*, *H3A*, *NCX1*, *NT3*, *POMC*, *RAG1* regions 1 and 2, *RAG2*, *RHO*, *SIA*, *SLC8A3*, *TNS3*, and *TYR*) and 8 mt markers (*12S*, *16S*, *CO1* regions 1 and 2, *CYTB*, *ND1*, *ND2*, and *ND4*) frequently used in frog phylogenetic studies and supermatrices.

### Phylogenetic Results

**Fig. 2.**
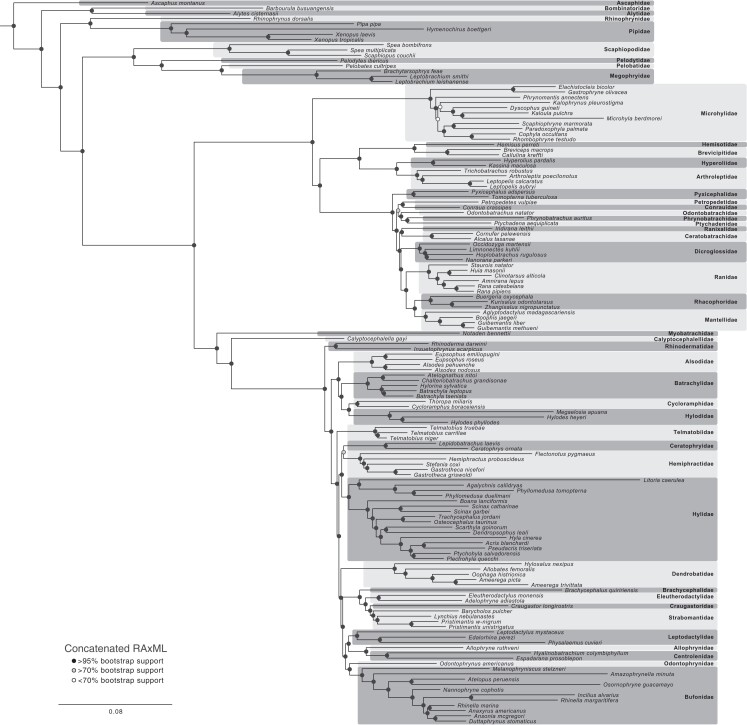
Phylogenetic estimate of anurans based on the concatenated ML analysis of 3,784 UCEs using RAxML. Scale bar represents substitutions per site. The bootstrap support value for each node is given in supplementary material S5 and figure S1, Supplementary Material online.

We first analyzed the UCE data alone, with concatenated maximum likelihood (ML) analysis (RAxML) and species-tree analysis (ASTRAL-III). The ML analysis used 128 threads on CIPRES, required ∼40-Gb memory and an average of ∼30 min per tree optimization, and ran for 5 h (∼650 CIPRES hours; note we provide these values for all ML analyses for comparison). The log-likelihood of the best ML tree was −42,126,237. The estimated ML and species trees were broadly similar to each other (fig. 1; supplementary material S1 and fig. S2 and supplementary material S5 and figs. S1 and S2, Supplementary Material online) and to previous estimates. However, we found alarming patterns in the ASTRAL-III tree for certain taxa (supplementary material S1, Supplementary Material online, support in supplementary fig. S2, Supplementary Material online; gene concordance in supplementary fig. S3, Supplementary Material online). Specifically, *Spea bombifrons* was placed near the base of Hyloidea, rather than with other *Spea* in Scaphiopodidae. Similarly, Litoria caerulea was placed near the base of Hyloidea, rather than with other Hylidae. Finally, two genera of Leptodactylidae (*Edalorhina* and *Physalaemus*) were together placed as the sister to Bufonidae, rather than with the confamilial genus *Leptodactylus* (which was sister to Terrarana + Bufonidae). These unusual placements were all strongly supported by ASTRAL-III. In contrast, in the concatenated analysis (fig. 2), these taxa were placed where expected with strong support (i.e., in *Spea*, Hylidae, and Leptodactylidae). Given these problematic results from ASTRAL-III, we focus primarily on the concatenated ML results. Given the large number of taxa and closely spaced nodes, for all trees we present the full tree with detailed support values in supplementary material S5, Supplementary Material online and use summary trees and support values in the main text and elsewhere. Note that the quartet analysis of gene tree congruence shows that there are conflicts between gene trees over many relationships, especially those with shorter branch lengths in the UCE tree, but most relationships are strongly supported nonetheless (supplementary material S5 and figs. S1–S3, Supplementary Material online).

**Fig. 3.**
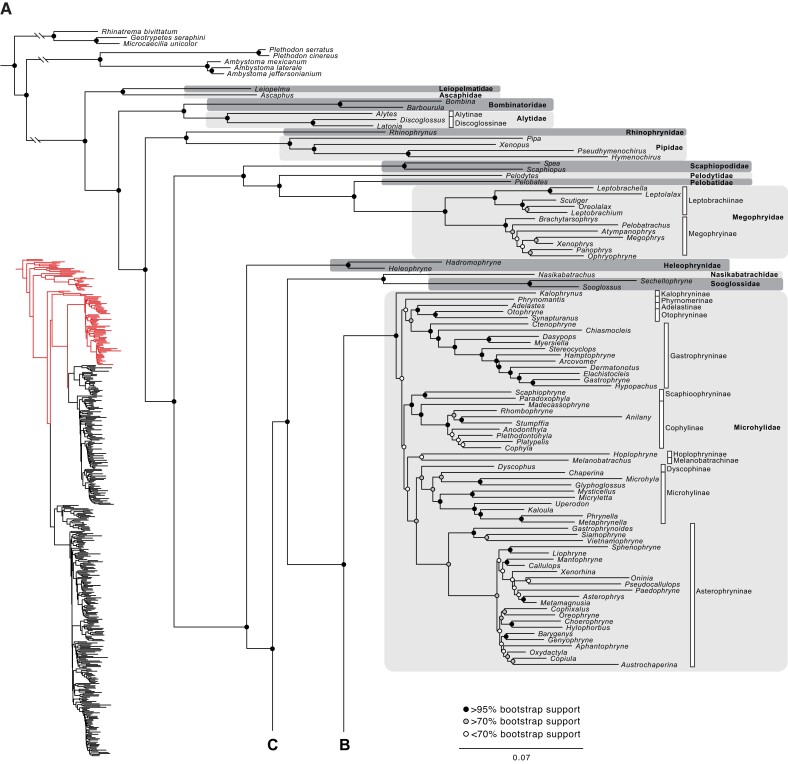

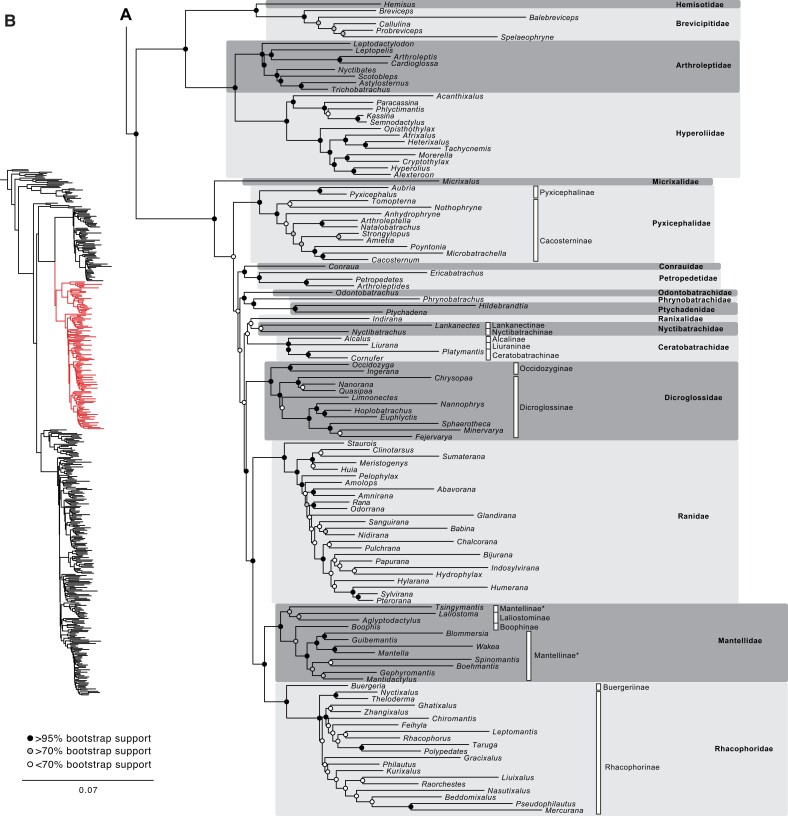

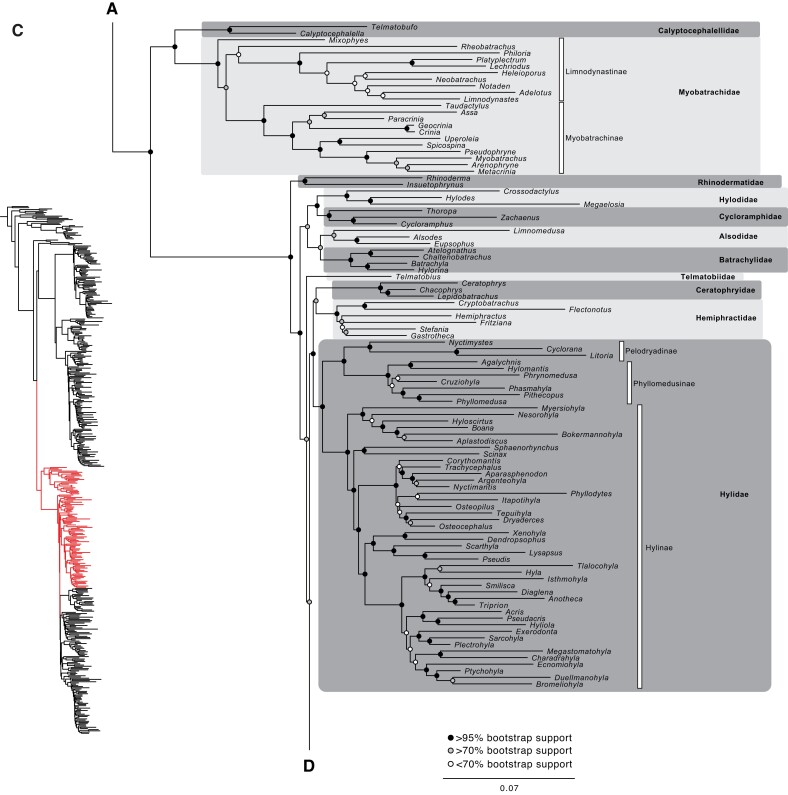

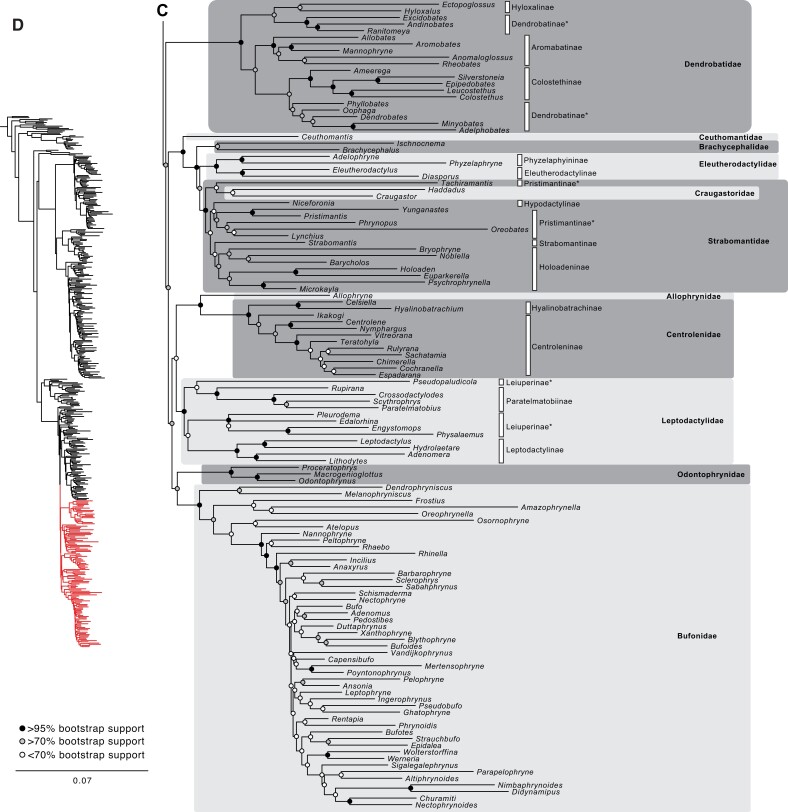
Concatenated ML analysis of the gigamatrix (combined UCE-supermatrix) with 4,091 markers. Scale bar represents substitutions per site. The analysis was partitioned. The phylogenetic tree is shown across four panels (A–D), with letters on branches representing connection points to the indicated panel. The exact bootstrap support value for each node is given in supplementary material S5 and figure S6, Supplementary Material online. White bars above genera indicate subfamilies (following the taxonomy of AmphibiaWeb 2020 used elsewhere in the paper). Asterisked subfamilies are nonmonophyletic. Note that many families lack subfamilies.

We next analyzed the gigamatrix data set (combined UCE + supermatrix) of 441 ingroup taxa. Among these ingroup taxa, 312 (70.7%) had >90% missing data, and 187 of these 312 (42.5%) had >99.5% missing data. We conducted a preliminary ML analysis of the gigamatrix using unpartitioned data, the GTR + CAT setting, and ten alternate runs on distinct starting trees. The unpartitioned analysis used 128 threads on CIPRES, required ∼33-Gb memory and an average of ∼5 h per tree optimization, and ran for 40 h (∼5,000 CIPRES hours). The log-likelihood of the best tree was −52,609,008. The topology and support values of this tree (supplementary material S1 and fig. S3 and supplementary material S5 and fig. S4, Supplementary Material online) were similar to those based on the partitioned analysis (fig. 3). These results suggest that choices about partitioning do not strongly impact our conclusions.

We then conducted a partitioned analysis of the gigamatrix using the GTR + gamma model and ten alternate runs on distinct starting trees. These partitioned analyses of the gigamatrix used 128 threads on CIPRES, required ∼132-Gb memory, required an average of ∼42 h per tree optimization, and ran for ∼350 h (∼45,000 CIPRES hours). The log-likelihood of the best tree found was −52,497,828. Although our best tree search was limited in scope, the ten trees were similar. The average normalized Robinson-Foulds distance (Robinson and Foulds 1981) among the ten trees was 0.06 (±0.03 SD). The average absolute Robinson–Foulds distance was 56 (±17 SD), indicating that an average of 56 nodes differed between the trees (among the 457 nodes present per tree). Differing nodes were within families and were associated with low bootstrap support (supplementary material S5 and fig. S5, Supplementary Material online).

In the concatenated ML analysis of the gigamatrix, almost all taxa were placed in the expected higher-level clades and families (fig. 3A–D). One family was nonmonophyletic (Strabomantidae), but monophyly of this family was rejected previously with supermatrix (e.g., Pyron and Wiens 2011) and phylogenomic analyses (Barrientos et al. 2021). Thus, taxa were not misplaced among families simply because they had extensive missing data. Results were similar for subfamilies within families (see supplementary material S1, Supplementary Results, Supplementary Material online). Among the 35 subfamilies represented by two or more genera, 31 (88.6%) were monophyletic. Among the four that were not, three were also nonmonophyletic in the supermatrix tree (Leiuperinae, Mantellinae, and Pristimantinae). Thus, their nonmonophyly is not explained by the gigamatrix approach. The one exception (Dendrobatinae) was nonmonophyletic in the gigamatrix tree because of a difference in the placement of a strongly supported clade of three genera (not a single taxon of uncertain placement).

Relationships among families were generally strongly supported in the gigamatrix tree (mean bootstrap = 88.5%; 53 nodes), but some relationships were weakly supported (fig. 3; supplementary material S5 and fig. S6, Supplementary Material online). Among families, we found especially weak support within Ranoidea (fig. 3B). Therefore, we performed an analysis to identify rogue taxa (i.e., with highly unstable placement). RogueNaRok (Aberer et al. 2013) identified 29 rogue taxa in the supermatrix phylogeny (supplementary material S1 and table S2, Supplementary Material online). Removal of the rogue taxa greatly improved branch support in some parts of the tree, especially relationships among families within Natatanura and Hyloidea and within some portions of Bufonidae and Microhylidae (supplementary material S1 and fig. S5 and supplementary material S5 and fig. S7, Supplementary Material online). The 29 rogue taxa had significantly more missing data than the other 412 taxa (mean for rogues = 97.96% missing data, nonrogue mean = 86.13%; *P* < 0.0001, *W* = 2,266.5; from nonparametric Wilcoxon test in R). Yet, one rogue taxon (*Duttaphrynus*) had 2,529 UCEs.

The concordance among genes and sites on each branch of the gigamatrix tree are visualized in supplementary materials S7 and S8, Supplementary Material online, respectively. The numbers of genes and sites that are decisive (informative) for each branch are shown in supplementary materials S9 and S10, Supplementary Material online. The latter are especially useful for visualizing how the amount of nonmissing data varies among branches, with among-family relationships often based on thousands of genes, and relationships within families often based on less than 10 genes (given that many genera are included based on few markers). Some of the weakly supported relationships among families are also based on very few genes, given the limited number of genes in certain taxa (e.g., Micrixalidae).

There was also considerable heterogeneity in branch lengths among terminal taxa in this tree (fig. 3). We address this in supplementary material S1, Supplementary Results, Supplementary Material online. The heterogeneity seemed to be related to heterogeneity in the type of data present in each terminal taxon, not the amount of missing data alone. Specifically, longer branches seemed to be associated with faster evolutionary rates in the mt markers for taxa with only mt markers or a similar number of mt and nuclear markers (i.e., those with legacy data alone), whereas branch lengths were shorter in taxa having a preponderance of nuclear markers (e.g., those with UCE data).

We then conducted a partitioned analysis of the supermatrix using the GTR + gamma model and 50 alternate runs on distinct starting trees. This tree is summarized in supplementary material S1 and figure S4, Supplementary Material online. The supermatrix analyses used 24 threads on CIPRES, required ∼1-Gb memory and an average of ∼1 h per tree optimization, and ran for a total of ∼70 h (∼1,600 CIPRES hours). The log-likelihood of the best supermatrix tree was −1,287,346.

We compared the percentage of shared nodes and mean bootstrap values among families for the concatenated ML trees from the UCE-only data (fig. 2; supplementary material S5 and fig. S1, Supplementary Material online), gigamatrix (combined UCE + supermatrix) data set (fig. 3; supplementary material S5 and fig. S6, Supplementary Material online), and supermatrix (supplementary material S5 and fig. S8, Supplementary Material online). When comparing the UCE tree to the gigamatrix tree, we found that 93.5% of comparable nodes among families were shared (43/46), with only three nodes in conflict (supplementary material S1 and table S3, Supplementary Material online). The UCE data set has only 47 families, reducing comparable nodes. When comparing the gigamatrix and supermatrix trees, only 62.3% of nodes among families were shared (33/53). This difference in proportions (0.935 vs. 0.623) was significant using a chi-squared test in R (*P* = 0.0002). Only 56.5% of nodes among families were shared between the UCE and supermatrix trees (26/46). Mean bootstrap support for among-family relationships (supplementary material S1 and table S3, Supplementary Material online) was highest for the UCE tree (mean = 99.3%; 46 nodes), lower for the gigamatrix tree (mean = 88.5%; 53 nodes), and lowest for the supermatrix tree (mean = 67.1%; 53 nodes). These differences were significant using a nonparametric Wilcoxon test in R (UCE vs. gigamatrix: *P* = 0.0003, *W* = 757.5; gigamatrix vs. supermatrix: *P* = 0.0018, *W* = 1875). In summary, our results suggest that adding the UCE data to the supermatrix yields a tree that is far more similar to the UCE tree and has higher bootstrap support than the tree based on the supermatrix data alone (for relationships among families). Furthermore, taxa were generally placed in the expected families and subfamilies (supplementary material S1, Supplementary Results, Supplementary Material online), regardless of their missing data.

We compare our phylogenetic results to those of other recent studies in supplementary material S1, Supplementary Results, Supplementary Material online. In short, we found that our gigamatrix tree was similar to those from other phylogenomic studies and not previous supermatrix trees.

We also estimated time-calibrated versions of the gigamatrix tree, both with and without rogue taxa (supplementary materials S11 and S12, Supplementary Material online). The estimated divergence dates from these trees are broadly similar to those from other recent studies, with some exceptions (supplementary materials S1 and tables S4 and S5, Supplementary Material online).

The treefiles from these analyses are available as supplementary materials S13–S21, Supplementary Material online. These include trees from UCE data alone (likelihood, supplementary material S13, Supplementary Material online; ASTRAL, supplementary material S14, Supplementary Material online), the supermatrix (supplementary material S15, Supplementary Material online), and the gigamatrix with and without rogue taxa (supplementary materials S16 and S17, Supplementary Material online), with time calibration (supplementary materials S18 and S19, Supplementary Material online), and with gene and site concordance factors for each node (supplementary material S20 and S21, Supplementary Material online).

## Discussion

The sequence data currently available for molecular phylogenetics are wildly heterogeneous, with data from thousands of genes for some species and only one or two genes for most others (and everything in between). Can we perform combined analyses that include species with these disparate amounts of data? And regardless of whether we can, should we?

Here, we show that such combined (gigamatrix) analyses are possible and potentially advantageous. We were able to include most anuran genera (97%) in our gigamatrix tree (combined UCE + supermatrix), even though 42.5% of the terminal taxa had >99.5% missing data and many taxa had data from only one or two genes. Almost every genus was placed in the expected family and subfamily. Overall, we found little evidence that highly incomplete taxa were misplaced in the gigamatrix tree.

We also found that higher-level relationships in the gigamatrix (supermatrix + UCE) tree generally reflected those from the phylogenomic (UCE) data set. For comparable among-family nodes, 93.5% of the nodes in the gigamatrix tree were shared with the UCE-only tree, whereas only 62.3% were shared with the supermatrix tree. Moreover, the mean bootstrap support for these among-family nodes was higher in the gigamatrix tree than the supermatrix tree (88.5% vs. 67.1%), but not as high as in the UCE-only tree (99.3%). Importantly, many family-level relationships in the gigamatrix tree were very different from a supermatrix study based on 15 genes (Jetz and Pyron 2018). For example, many major clades found in the UCE-only tree were not monophyletic in that supermatrix tree, including the major hyloid clades Amazorana and Commutabirana and the clade in Ranoidea uniting Afrobatrachia and Natatanura. These clades were also supported in a phylogenomic study based on hundreds of nuclear markers (Hime et al. 2021). Our results suggest that the higher-level relationships and their support values in the gigamatrix tree are generally determined by the data set with the most markers (phylogenomic), even though most taxa are included based on a much smaller number of markers.

Readers may have two main questions about our study. First, do we know that the gigamatrix approach recovers accurate phylogenies? Second, do these findings apply to other clades besides frogs? First, there are many simulation and empirical studies showing that highly incomplete taxa can be accurately placed in phylogenies (review in Wiens and Morrill 2011). Thus, we found here that 187 taxa, each with >99.5% missing data, were all placed in their expected families, often with strong support. Although this result might sound unbelievable, the extensive missing data in these taxa is a function of the large numbers of genes in the other taxa. Thus, our results are consistent with theory suggesting that the accuracy with which taxa are placed depends on the data that they have, not the amount that they lack (Wiens 2003). Moreover, previous simulation and empirical studies suggest that adding data sets with missing data in many taxa (e.g., the phylogenomic data set added to the supermatrix) can be beneficial for recovering known clades (simulations) and well-established clades (empirical studies), relative to analyzing a smaller set of characters with more complete data in all taxa (e.g., simulations: Gouveia-Oliveira et al. 2007; Wiens 2009; Wiens and Morrill 2011; Talavera et al. 2022; empirical: Wiens et al. 2005; Cho et al. 2011; Jiang et al. 2014; Streicher et al. 2016; Talavera et al. 2022). This combination is beneficial because of the improved accuracy associated with the data sets with more characters but fewer taxa. Note that we did not conduct new simulations based on our empirical data sets, since the large size of these data sets makes them impractical for simulations. Overall, our results here uniquely support and extend these previous studies by showing that their theoretical conclusions seem to apply to very large-scale data sets with thousands of markers, millions of characters, and extreme amounts and proportions of missing data.

The second question is whether our results apply to other clades. In some ways, the concordance between our results and the previous simulation and empirical studies cited above already answers that question. The generality of our conclusions is supported by many studies showing that highly incomplete taxa can be accurately placed in phylogenies and that adding data sets having data for only some taxa can be beneficial. Furthermore, focusing more specifically on the gigamatrix approach, our results are concordant with a previous study that combined phylogenomic (44 genes and 161 species) and supermatrix data sets (12 genes and 4,161 species) in squamate reptiles, albeit with a smaller phylogenomic data set (Zheng and Wiens 2016). Those authors addressed the same three questions that we did here. First, our study found no cases in which highly incomplete taxa were placed in the “wrong” family (i.e., based on previous taxonomy and previous molecular and morphological studies). This was also generally true for the squamate study. Zheng and Wiens (2016) did find a few nonmonophyletic families (i.e., Cylindrophiidae and Lamprophiidae), but their nonmonophyly was also found in supermatrix studies (e.g., Figueroa et al. 2016), and so these cases do not appear to be caused by the gigamatrix approach. Second, higher-level relationships in the squamate gigamatrix tree more strongly resembled those from the phylogenomic data set (90% of higher-level nodes shared) than the supermatrix data set (77% shared). Similarly, we found here that for among-family nodes, 93.5% of the nodes in the gigamatrix tree were shared with the phylogenomic tree, whereas only 62.3% were shared with the supermatrix tree. Thus, in both cases, the gigamatrix tree generally reflected the phylogenomic data for higher-level relationships, as intended. Third, Zheng and Wiens (2016) found that mean bootstrap support for higher-level nodes in the gigamatrix tree was higher than the supermatrix tree, but not significantly (74.1% vs. 71.2%). These gigamatrix bootstrap values were also significantly lower than in the 44-gene data set (74.1% vs. 88.9%). Here, we found that the mean bootstrap support for these among-family nodes in the gigamatrix tree was significantly higher than in the supermatrix tree (88.5% vs. 67.1%) but significantly lower than the UCE-only tree (99.3%). Thus, mean support for the higher-level relationships was consistently higher in the gigamatrix tree relative to the supermatrix tree, as intended. Overall, the results are broadly concordant between the two studies, but we suggest that the much larger number of markers in our phylogenomic data set here help explain why the phylogenomic data set in frogs more strongly influenced the gigamatrix tree than in squamates (both in determining higher-level relationships and increasing bootstrap support for those relationships). Thus, in the context of a gigamatrix analysis, incorporating a larger phylogenomic data set (i.e., more markers) seems to be more beneficial, despite increasing the overall amount and proportion of missing data in the matrix.

Empirical analyses in butterflies by Talavera et al. (2022) also showed that combining multilocus data sets containing few taxa with single-gene DNA-barcoding data sets with many taxa yielded higher-level phylogenies more similar to the multilocus data sets (similar to frogs and squamates). They also performed simulations and found that this combined approach improved accuracy relative to analyzing DNA-barcoding data alone. These results further support the generality of our conclusions about the gigamatrix approach. Furthermore, considering both our study and that of Talavera et al. (2022) suggests that it should be possible to combine DNA-barcoding data sets (with a single gene for many taxa) with phylogenomic data sets (containing hundreds or thousands of markers for a more limited set of taxa). Most importantly, our results suggest that the extreme levels of missing data in taxa with DNA-barcoding data alone should not be an impediment to their correct placement in a gigamatrix tree.

Our results also suggest that a gigamatrix approach might have advantages relative to simply constraining the supermatrix analysis to match the phylogenomic tree. For example, our concatenated UCE-only tree showed the grouping of Calyptocephalellidae + Myobatrachidae to be paraphyletic with strong support. However, the placement of these two families as sister taxa has been supported in most recent phylogenetic analyses of anurans (Feng et al. 2017; Jetz and Pyron 2018; Hime et al. 2021). Intriguingly, the gigamatrix tree strongly supported Myobatrachidae + Calyptocephalellidae. This shows that these much smaller data sets (i.e., fewer genes) were together able to overturn some relationships from the much larger data set, even though they differed in size by more than 10-fold (307 vs. 3,784 genes). This pattern would have been missed if we had simply assumed that the UCE tree was correct. We found other cases in which the gigamatrix tree differed from the UCE tree alone (e.g., within Neoaustrarana), although in these other cases, the relationships are not as well established. The ability of the data set with fewer genes to overturn relationships from the data set with many more genes almost certainly results (at least in part) from there being some support in the larger data set for the alternative relationship (e.g., many UCEs support monophyly of Myobatrachidae + Calyptocephalellidae; Streicher et al. 2018). Therefore, a constrained approach may not be optimal, although it may be more computationally efficient.

Along these lines, a recent study combined phylogenomic data with GenBank data to estimate a large-scale phylogeny of 4,705 mammal species (Álvarez-Carretero et al. 2021). In that study, higher-level relationships (and associated divergence dates) were estimated and fixed based on phylogenomic data (15,268 genes for 72 species), and species-level relationships and divergence times were estimated separately within 13 clades using a data set of 182 genes. These species-level trees were then combined to make the overall phylogeny. This approach seems reasonable, although those authors had to “manually adjust” aspects of their tree to match expected relationships. However, we found that the addition of smaller numbers of markers can help resolve higher-level relationships, rather than simply assuming that the phylogenomic data set is always correct. Furthermore, there can be a continuum in the sizes of the phylogenomic and species-level data sets. For example, our supermatrix data set includes data from a study based on next-generation sequencing and hundreds of markers (Hime et al. 2021), and one taxon in the phylogenomic data set had data from only 42 UCEs. The gigamatrix approach does not require making a distinction between phylogenomic and supermatrix data sets.

We do acknowledge several potential disadvantages of the gigamatrix approach (table 1). The most important disadvantage may be that it is computationally intensive. Our initial goal was to include ∼5,000 anuran species in the gigamatrix. After much time and effort, we found that this was simply not computationally tractable at present (neither on CIPRES nor the UK Crop Diversity Bioinformatics High Performance Computing Resource, nor with the help of A. Stamatakis and his considerable expertise and resources). For example, all analyses crashed before completing a single likelihood optimization. Our genus-level sampling represented a compromise. However, our results show that analyses of gigamatrix data sets are feasible for hundreds of taxa and thousands of markers and are not misled by the extensive missing data in most taxa. We suspect that analyses combining thousands of taxa and markers (in at least some taxa) will become computationally tractable in the near future.

Another disadvantage of the gigamatrix approach is that the placement of many taxa can depend on only a few markers and may be correspondingly uncertain. For example, we found that relationships among families of Natatanura were strongly supported by UCE data (fig. 2) but weakly supported when supermatrix taxa were added (fig. 3). The uncertain placement of Micrixalidae (lacking UCE data) seemed to contribute greatly to the low support. We used RogueNaRok (Aberer et al. 2013) to identify rogue taxa, and deleting rogue taxa improved support in several parts of the tree (supplementary material S1 and fig. S5 and supplementary material S5 and fig. S6, Supplementary Material online). Most rogue taxa had data for only a few genes (but one had UCE data; supplementary material S1 and table S2, Supplementary Material online). Overall, we caution that the gigamatrix approach can yield disappointing support values in some parts of the tree. We assume that this occurs primarily because relationships among many taxa are only addressed by a limited number of genes. Nevertheless, bootstrap support for higher-level relationships was substantially higher in the gigamatrix tree than the supermatrix tree, but not as high as in the UCE-only tree.

We also found considerable heterogeneity in the branch lengths among terminal taxa in the gigamatrix tree (supplementary material S1, Supplementary Results, Supplementary Material online). Our analyses suggest that this heterogeneity is related to some taxa having mostly fast-evolving mt genes, whereas branch lengths were more homogeneous among taxa with many nuclear genes. These results, as well as previous analyses (e.g., Pyron et al. 2011; Wiens and Morrill 2011; Jiang et al. 2014), suggest that branch length variation is not simply related to the amount of missing data alone. Importantly, we did not find clear evidence here that this heterogeneity has other negative consequences (e.g., causing long-branch taxa to be placed in the wrong family). Further, previous analyses suggest that the presence of extensive missing data in most genes may have limited impact on widely used tree-dating methods (e.g., Zheng and Wiens 2015; Talavera et al. 2022). Indeed, we found that the divergence dates estimated from our gigamatrix tree were broadly similar to other recent estimates (supplementary material S1 and table S4, Supplementary Material online).

Another potential disadvantage of the gigamatrix approach is that it may disallow use of species-tree analyses. These species-tree approaches can perform well if taxa with missing data are randomly distributed across markers but may be unreliable if missing data are nonrandomly distributed (Xi et al. 2016). In our gigamatrix, missing data are not randomly distributed but instead are concentrated in ingroup taxa lacking UCE data (68.7% of taxa). A potential solution would be to estimate higher-level relationships using a species-tree analysis of the phylogenomic data and then perform a supermatrix analysis that constrained higher-level relationships to the species-tree estimate. However, we found that the species-tree analysis of the UCE data gave some problematic but strongly supported results (e.g., nonmonophyly of Hylidae, Leptodactylidae, and Scaphiopodidae). We have also found other cases where species-tree analyses give problematic results that conflict with concordant evidence from concatenated molecular markers and from morphology (Streicher et al. 2018; Portik and Wiens 2021). Species-tree analyses have important advantages relative to concatenated analyses in theory (e.g., Kubatko and Degnan 2007; Edwards 2009; Leaché and Rannala 2011). Yet our comparisons involving empirical data sets suggest that additional work may be needed to overcome the problems observed in practice. Whether these problems are specific to UCE data or apply broadly to phylogenomic data is a question of urgent importance, both for those who use UCE data and those who do not.

We also caution that the promising results from frogs (and squamates; Zheng and Wiens 2016) are not a guarantee that the gigamatrix approach will perform well in every case. We suspect that an important key to its success in frogs is that numerous researchers have used the mt 12S and 16S genes in systematic studies in frogs, such that almost all taxa had data for one or more of these genes here (supplementary material S4, Supplementary Material online). This allowed fine-scale placement of many sampled frog species. DNA-barcoding data from the same fast-evolving mt gene (cytochrome oxidase I) from large numbers of taxa may have similar benefits (Talavera et al. 2022). A lack of overlap among genes sampled for different taxa may be especially problematic (Sanderson et al. 2010) and is a somewhat distinct problem from the amount of missing data per se. This lack of overlap can also make a supermatrix approach problematic. Overall, we predict that the gigamatrix approach will be most successful when combining a supermatrix that can fully resolve species-level relationships with a phylogenomic data set that can strongly resolve higher-level relationships.

Finally, we acknowledge that additional work is still needed on frog phylogeny. The gigamatrix tree estimated here should generally be an improvement relative to previous estimates. However, estimation will continue to improve as more genes are added for more taxa. We also note that UCEs are only one approach for sampling thousands of markers from a genome. For example, all species lacked data for at least some of the 3,784 UCEs (the maximum number in any species was 2,880). Perhaps the biggest drawback of phylogenomic data is that the number of species sequenced is often limited, as broad sampling remains expensive and labor-intensive. Including more markers with some missing data appears to be preferable to sampling a smaller number of more complete markers (Streicher et al. 2016). Future studies will doubtlessly increase taxon sampling, sampling of markers, and completeness per marker. New approaches might also greatly increase the number of markers per species (e.g., to 13,500 per species; Hutter et al. 2022). In the short term, such extensive gene sampling for a limited number of species will only exacerbate the disparities in markers available for each taxon. Fortunately, our results here suggest that combining such expanded phylogenomic data sets with existing phylogenomic and supermatrix data sets should not be problematic.

## Conclusions

Our study shows that it is possible to combine phylogenomic data sets containing thousands of genes with supermatrix data sets consisting of hundreds of taxa (some with data from only one or two genes). We refer to this combination as the gigamatrix approach. We found that the higher-level relationships from the gigamatrix tree are generally strongly supported and resemble those from the phylogenomic analysis, rather than from the supermatrix analysis. We also found that all taxa were placed in the expected families, even though 42.5% of the taxa had >99.5% missing data. We showed that missing data were not an impediment to combining data sets with wildly disparate numbers of taxa and genes, opening the door to new types of analyses. Most importantly, these new types of analyses can potentially replace supermatrix analyses based on limited numbers of genes for large numbers of taxa.

## Materials and Methods

### Overview

Given the length restrictions of the journal, we give a brief sketch of the methods used here and give full details in supplementary material S1, Supplementary Methods, Supplementary Material online.

### Taxon Sampling for UCE Data

We assembled UCE data from 138 species, representing 47 of 54 anuran families (87%; supplementary material S2, Supplementary Material online), using published and new data. Streicher et al. (2018) sampled extensively within Hyloidea (and outgroups), including data for 54 species for up to 2,214 UCEs. We used these data and UCE data from seven species from Hutter et al. (2022), who sampled broadly across Anura and targeted 2,085 UCEs.

We obtained new data for species of Ranoidea (*n* = 42) and Archaeobatrachia (*n* = 11). We sampled 17 of 18 ranoid families (all but Micrixalidae). Within Archaeobatrachia, we sampled all families but Leiopelmatidae. We also sampled additional species (*n* = 17) from several diverse families in Hyloidea.

Finally, we used published genomes to extract UCE data from 24 species, including 7 frog species, various amphibian outgroups (Caudata and Gymnophiona), and more distant nonamphibian outgroups. Details of taxon sampling are in supplementary material S2, Supplementary Material online. The details for collecting new UCE data, processing these data, and combining them with published data are in supplementary material S1, Supplementary Methods, Supplementary Material online.

### Genus-Level Supermatrix with GenBank Data

We generated a supermatrix to include as many genera as possible. We identified 22 molecular markers that are widely used in anuran phylogenetics. These included 15 nuclear genes (*BDNF*, *BMP2*, *CMYC*, *CXCR4*, *H3A*, *NCX1*, *NT3*, *POMC*, *RAG1*, *RAG2*, *RHO*, *SIA*, *SLC8A3*, *TNS3*, and *TYR*) and seven mt genes (12S, 16S, *CO1*, *CYTB*, *ND1*, *ND2*, and *ND4*). For both *RAG1* and *CO1*, multiple primer designs have led to two nonoverlapping regions being sequenced, and we therefore treated them as distinct markers. We refer to these 24 markers as “legacy” markers.

We also included two nuclear data sets having fewer taxa. The first data set included 92 markers and 156 species (Feng et al. 2017; Tu et al. 2018). Three of these markers overlapped with the legacy markers. The second data set included 230 taxa with 220 nuclear markers (Hime et al. 2021). Twenty-six markers were shared between these data sets (283 combined). In total, the supermatrix data set included 307 non-UCE markers.

### Phylogenetic Analyses

The UCE data were analyzed using both concatenated ML using RAxML (Stamatakis 2014) and a gene-tree summary method (ASTRAL-III; Zhang et al. 2017). All other data sets (i.e., supermatrix and gigamatrix) were analyzed with concatenated ML. Details are in supplementary material S1, Supplementary Methods, Supplementary Material online, including computational resources used and final likelihoods.

## Supplementary Material

msad109_Supplementary_Data

## Data Availability

Supplementary materials S1–S21 will be available on Dryad and Zenodo (doi:10.5061/dryad.f7m0cfz0n) and are currently available through https://datadryad.org/stash/share/3n1-6ozz0Lw-l4ihTcDfPG_hmgVqKYHF3_F0LmsThRs. In addition, we provide the UCE sequences from all sources, individual alignments, and the supermatrix and gigamatrix files on an Open Science Framework project page: https://osf.io/fzw3x/. These files are generally too large for Dryad. This project page also contains the phylogenetic trees created from each data set. Newly sequenced samples have been deposited in the NCBI Sequence Read Archive (SRA): Accessions SRR24313344–SRR24313392. ***Conflict of interest statement.*** The authors declare no competing interests.
