# Supplementary material for: Redefining Possible: Combining Phylogenomic and Supersparse Data in Frogs
Source: Mol Biol Evol. 2023 May 4;40(5):msad109. doi: 10.1093/molbev/msad109 (PMC10202597; doi:10.1093/molbev/msad109)
Supplement: msad109_Supplementary_Data [file msad109_supplementary_data.zip › Supplementary File Guide.docx]

**Supplementary Files**

Supplementary File S1. Supplementary Methods, Results, References, Figures (S1–S5), and Tables (S1–S5)

Supplementary File S2. Information on UCE data

Supplementary File S3. Selection of species for the supermatrix

Supplementary File S4. GenBank accession numbers for all markers and taxa in the legacy and NPCL datasets

Supplementary File S5. Figures S1–S8.

Supplementary File S6. The three partitioning schemes considered in this study

Supplementary File S7. Figure showing the gigamatrix tree with gene concordance factors for each branch.

Supplementary File S8. Figure showing the gigamatrix tree with site concordance factors for each branch.

Supplementary File S9. Figure showing the gigamatrix tree with the number of decisive genes for each branch.

Supplementary File S10. Figure showing the gigamatrix tree with the number of decisive sites for each branch.

Supplementary File S11. Figure showing the time-calibrated gigamatrix tree including all taxa.

Supplementary File S12. Figure showing the time-calibrated gigamatrix tree with 29 rogue taxa excluded.

Supplementary File S13. Concatenated maximum likelihood treefile for the UCE data.

Supplementary File S14. ASTRAL treefile for the UCE data.

Supplementary File S15. Supermatrix treefile.

Supplementary File S16. Gigamatrix treefile, all taxa included.

Supplementary File S17. Gigamatrix treefile, rogue taxa excluded.

Supplementary File S18. Time-calibrated gigamatrix treefile, all taxa included.

Supplementary File S19. Time-calibrated gigamatrix treefile, 29 rogue taxa excluded.

Supplementary File S20. Gigamatrix treefile with gene concordance factors and related measures for each branch.

Supplementary File S21. Gigamatrix treefile with site concordance factors and related measures for each branch.
