# Supplementary material for: Redefining Possible: Combining Phylogenomic and Supersparse Data in Frogs
Source: Mol Biol Evol. 2023 May 4;40(5):msad109. doi: 10.1093/molbev/msad109 (PMC10202597; doi:10.1093/molbev/msad109)
Supplement: msad109_Supplementary_Data [file msad109_supplementary_data.zip › Supplementary File S1.pdf]

Supplementary File S1

Redefining Possible: Combining Phylogenomic and Supersparse Data in Frogs

Supplementary Methods

Supplementary Results

Supplementary Figures

Figure S1. Swarm plots of alignment characteristics for different marker classes.
Figure S2. Phylogenetic estimate of anurans based on the species-tree analysis of 3,784 UCE markers using ASTRAL-III.
Figure S3. Maximum likelihood tree from the unpartitioned gigamatrix analysis.
Figure S4. Estimated tree from maximum likelihood analysis of the supermatrix.
Figure S5. Tree from concatenated maximum likelihood analysis of the gigamatrix, with rogue taxa removed.

Supplementary Tables

Table S1. Voucher information for tissue samples used this study.
Table S2. List of rogue taxa identified by RogueNaRok.
Table S3. Comparison of congruence and support among higher-level nodes in the gigamatrix, UCE, and supermatrix trees
Table S4. Comparison of estimated clade ages in this study (gigamatrix tree, all taxa included) to a selection other recent trees
Table S5. Comparison of estimated clade ages in this study (gigamatrix tree, rogue taxa excluded) to a selection other recent trees

Supplementary Methods

Taxon Sampling for UCE data

We assembled UCE data from 138 species, representing 47 of 54 anuran families (87%, Supplementary File S2), using published and new data. Streicher et al. (2018) sampled extensively within Hyloidea (and outgroups), including data for 54 species for up to 2,214 UCEs. We used these data and UCE data from 7 species from Hutter et al. (2022), who sampled broadly across Anura and targeted 2,085 UCEs.

We obtained new data for species of Ranoidea (*n*=42) and Archaeobatrachia (*n*=11). We sampled 17 of 18 ranoid families (all but Micrixalidae). Within Archaeobatrachia, we sampled all families but Leiopelmatidae. We also sampled additional species (*n*=17) from several diverse families in Hyloidea.

Finally, we used published genomes to extract UCE data from 24 species, including 7 frog species, various amphibian outgroups (Caudata, Gymnophiona), and more distant non-amphibian outgroups. Details of taxon sampling are in Supplementary File S2.

Details of UCE Data Collection

We extracted DNA from 67 tissue samples (Supplementary File S2, Table S1) using the Qiagen DNEasy Blood & Tissue Kit. DNA concentration was quantified using a Qubit DNA BR assay (Life Technologies), and 1,500 ng of DNA per sample was sheared using a BioRuptor (Diagenode). We used the FrogCap protocol to perform target capture for 21 of the 70 samples, following methods detailed in Hutter et al. (2022). For the other 49 samples, we performed a targeted UCE capture. Initially all samples were subjected to 10 cycles (30 sec on/30 sec off) to achieve a target size of 400–600 bp. Fragmentation was assessed using a BioAnalyzer 2100 (Agilent). Several samples required an additional 8 or 16 cycles for fragmentation. We prepared libraries from the fragmented DNA using the Kapa HyperPrep kit (Roche). We followed the standard protocol but used Serapure beads for cleanup steps and TruSeq DNA unique dual index adapters (IDT for Illumina). We used the myBaits UCE Tetrapods 5Kv1 probe set (8-reaction kit size, Arbor Biosciences) for captures, following the v4.01 protocol. We pooled 6–7 libraries for each capture reaction (1500 ng total input DNA) and performed hybridization for 24 hrs. Post-capture PCR amplification used 12 cycles, and the post-cleanup product was eluted in 12 µL of

buffer EB (10mM Tris-HCl). The eight capture products were pooled in equimolar amounts and sequenced on one lane of an Illumina HiSeq4000 with 150 bp paired-end (PE) reads.

UCE Data Processing

We obtained UCE data from a variety of sources, including new sequence data, published whole genomes, and multiple UCE target capture studies. To maintain consistency, we re-processed the published sequence data using the same analysis pipeline as our newly generated data. The major pipeline steps included adapter trimming and read-quality filtering, de novo assembly, and UCE identification using PHYLUCE version 1.6.6 (Faircloth 2016), and filtering based on coverage. The UCE sequences obtained from the new sequence data, whole genomes, and published datasets were then combined and analyzed using SuperCRUNCH (Portik and Wiens 2020). We describe these methods in greater detail below.

UCEs from Whole-Genome Datasets. We used published genomes to extract new UCE data for 12 amphibian species (including eight frogs, three caecilians, and one salamander) and nine outgroup species (Supplementary File S1). We downloaded all genomes in FASTA format and converted these files to 2bit format using the faToTwoBit program of the UCSC Genome Browser (Kent et al. 2002). PHYLUCE was used to align the tetrapod 5k UCE probe set (5,060 loci) to each 2bit genome file using LASTZ (Harris 2007). We then used PHYLUCE to extract all relevant UCE sequences, retaining up to 500 base pairs of flanking sequence per side. Finally, we used a script to relabel the sequences (https://github.com/dportik/phyluce-genomes-to-supercrunch) to ensure compatibility with SuperCRUNCH.

UCEs from Target-Capture Datasets. We obtained new sequence data for 67 samples, published sequence data from the NCBI Sequence Read Archive (SRA) for 58 samples, and previously assembled contigs for 10 species (Supplementary File S1). The SRA data included 54 frogs (Streicher et al. 2018) and four salamanders (Newman and Austin 2016; Denton et al. 2018), whereas the contig sets for 10 species came from Hutter et al. (2022). We used sratoolkit and fastqdump from the SRAtoolkit (v2.9.6) to obtain fastq files for the SRA samples and used Trimmomatic (Bolger et al. 2014) or FASTP (Chen et al. 2018) to remove adapters and low-quality reads from the fastq files (newly sequenced and SRA).

The Illumina sequence data were obtained using different instruments and contained different read lengths (100, 150, and 300 bp PE). Consequently, they required a flexible strategy

for assembly. We used VelvetOptimiser (https://github.com/tseemann/VelvetOptimiser) to de novo assemble each sample with Velvet (v.1.2.1; Zerbino and Birney 2008). Given the variation in read lengths across samples, we generated assemblies using a range of hash values (55–105, by increments of 10). This resulted in six Velvet assemblies per sample. We also used aTRAM2 (Allen et al. 2015, 2018) to perform an iterative, seeded assembly for each sample. For this method, we used the UCE Tetrapods 5Kv1 probe sequences as the bait sequences, and Velvet as the assembler. For each of the seven assemblies per sample (e.g., six from Velvet, one from aTRAM2), we used PHYLUCE to identify contigs matching UCEs. We used *phyluce_assembly_match_contigs_to_probes* to align the tetrapod 5k UCE probes to each contig set using LASTZ. We then ran *phyluce_assembly_get_match_counts* and *phyluce_assembly_get_fastas_from_match_counts* to extract sequences for all matched UCEs.

The published UCE data from Hutter et al. (2022) and the 18 samples with FrogCap UCE data were provided as contigs assembled through the pipeline in Hutter et al. (2022). These had been previously assembled using SPAdes v3.12 (Bankevich et al. 2012). For these data, we used PHYLUCE to identify and extract UCE sequences as described above.

We performed a final filtering step for all UCE contigs. For each assembly (Velvet, aTRAM2, or Spades), we used nucleotide BLAST (dc-megablast) to match the UCE contigs to the tetrapod 5k UCE probe set. We removed any UCE contig that matched probes for more than one UCE and removed any UCE contig that matched a probe containing a different UCE label. For the Velvet and aTRAM2 assemblies, we then mapped the reads to the filtered UCE contigs using BWA (Li and Durbin 2009). Picard (v.1.119; https://broadinstitute.github.io/picard/) was used to mark and remove PCR duplicates, and coverage was estimated using the depth module of Samtools (Li et al. 2009). We removed all UCE contigs with <5X coverage. Among the 1,205,752 starting UCE contigs, this action removed 225,009 (18.6%) low coverage contigs, resulting in the retention of 980,743 contigs. This coverage filter primarily affected contigs from Streicher et al. (2018), which were generated from substantially fewer reads (Illumina MiSeq) relative to the other datasets.

For samples assembled using Velvet, our goal was to maximize the number of UCEs obtained. We therefore compared the number of filtered UCEs obtained across the different hash values and selected the assembly with the highest number. For a given sample, the selected Velvet assembly and aTRAM2 assembly contained redundant UCE sequences, but each method

also resulted in unique UCE sequences that increased the overall yield of UCEs. We therefore kept the UCE sequences from both assemblies and used SuperCRUNCH to select the best sequence per UCE (see below). To investigate whether this approach produced more UCE sequences than either method alone, we also analyzed the Velvet and aTRAM2 contigs separately.

UCE SuperCRUNCH Analysis. We used SuperCRUNCH to create UCE-specific FASTA files, and for each file to perform sequence-similarity filtering, sequence selection, alignment, and trimming. To properly process local sequence data (i.e., data not downloaded directly from GenBank), SuperCRUNCH requires FASTA description lines to contain a unique identifier, taxon name, and locus abbreviation/description (mimicking NCBI GenBank format). We relabeled the UCE sequences obtained from whole genomes and target captures to comply with these criteria. All UCE sequences were combined into a single FASTA file, which contained a total of 417,373 sequences. SuperCRUNCH also requires a list of taxa and locus search terms to assemble marker-specific FASTA files. We obtained the taxon list from the UCE FASTA file (*Fasta_Get_Taxa.py*) and used the UCE 5k search-terms file included with SuperCRUNCH. The taxon list and search-terms files were used to run *Parse_Loci.py*, which produced 5,041 UCE files containing at least two sequences.

We performed similarity filtering for each UCE sequence set using *Cluster_Blast_Extract.py*. For each marker, this method clusters the sequences using CD-HIT-EST (Li and Godzik 2006), designates the largest cluster as a BLAST reference database, performs BLASTn searches for all sequences (including those in the reference cluster, excluding self-hits), and outputs sequences that are trimmed based on their merged BLAST hit coordinates (Portik and Wiens 2020). Following similarity filtering, we used *Filter_Seqs_and_Species.py* to select a single representative sequence per species per UCE, taking the longest available sequence if multiple sequences were present (two sequences could occur for a UCE for samples assembled with Velvet and aTRAM2). During selection, we enforced a 200-base pair minimum length to retain a sequence (following recommendations of Hosner et al. 2015). After the selection of representative sequences, we screened the UCE markers to ensure they contained at least 10 species using *Fasta_Filter_by_Min_Seqs.py*. We ensured that each UCE marker contained more than just outgroup sequences.

Sequence alignment and trimming were performed following recommendations for UCEs by Portik and Wiens (2021). We used MAFFT v7.475 (Katoh et al. 2002, 2005; Katoh and Standley 2013) with the automatic algorithm selection option and performed trimming with trimAl (Capella-Gutiérrez et al. 2009) using a gap-threshold value of 0.2. This trimming threshold removes columns containing gaps for more than 80% of the sequences present. Sequences were relabeled prior to trimming using *Fasta_Relabel_Seqs.py*, such that they only included species names (vs. full description lines). Finally, we concatenated the trimmed UCE alignments using *Concatenation.py*. We refer to this as the UCE dataset.

Assembling UCE Contigs

To maximize the overall yield of sequences, we used a strategy that involved combining two methods to assemble UCE contigs. Using both methods produced a combined total of 313,361 UCE sequences (185,517 from Velvet, 127,844 from aTRAM2). However, this combined set likely contained many redundant sequences (e.g., the same UCE locus was assembled for a species by both methods). To explore this idea, we used SuperCRUNCH to analyze each contig set separately and to analyze the combined contig set. The total number of sequences obtained by analyzing the aTRAM2 dataset alone was 85,895, and the analysis of the Velvet dataset alone produced only 179,088 total sequences. The combined analysis resulted in 209,791 total UCE sequences, with 125,743 resulting from Velvet assemblies and 84,048 resulting from aTRAM2. These results indicate that performing assembly with multiple methods and selecting sequences with SuperCRUNCH improved the overall yield of UCEs by ~30,000 sequences (relative to the Velvet results), with an average increase of ~200 sequences per sample.

Details of Supermatrix Construction

We generated a supermatrix to allow us to include as many anuran genera as possible. We identified 22 molecular markers that have been widely used in anuran phylogenetics (e.g., Pyron and Wiens 2011; Jetz and Pyron 2018). These included 15 nuclear genes (*BDNF, BMP2, CMYC, CXCR4, H3A, NCX1, NT3, POMC, RAG1, RAG2, RHO, SIA, SLC8A3, TNS3, TYR*) and seven mitochondrial (mt) genes (*12S, 16S, CO1, CYTB, ND1, ND2, ND4*). For both *RAG1* and *CO1,* multiple primer designs have led to two non-overlapping regions being sequenced, and we

therefore treated each of these markers as having two distinct regions. We refer to these 22 genes as the "legacy" markers since they have been widely used in previous phylogenetic studies.

In addition to these traditional GenBank markers, we also included two sets of nuclear markers that were available for smaller numbers of taxa. The first set included 92 markers sequenced by Feng et al. (2017) and Tu et al. (2018). These were available for a total of 156 frog species. These are a subset of the PCR-based nuclear protein-coding loci (NPCL) described in Shen et al. (2013). The second set included 230 taxa with 220 nuclear markers sequenced by Hime et al. (2021). These are conserved nuclear exon sequences obtained through anchored-hybrid enrichment (AHE; Lemmon et al. 2012). We determined that 26 markers were shared between these two datasets, with no taxonomic overlap. We ultimately constructed alignments for these shared markers using a combination of the AHE and NPCL sequences, but for categorization we considered these to be NPCL markers.

In total, we included 286 non-UCE markers. All sequences for the legacy and NPCL markers were available on GenBank. To obtain them, we downloaded all available sequences for Anura from NCBI on November 17, 2020. We obtained AHE sequences by downloading alignment files from the supplemental materials provided in Hime et al. (2021). To make the AHE data compatible with SuperCRUNCH, we stripped the alignments (i.e., removed all gaps), updated the taxon labels to be compatible with the taxonomy of AmphibiaWeb, assigned each sequence a unique identifier (e.g., "accession" number), and added gene-description terms. We downloaded the AmphibiaWeb taxonomy on November 17, 2020 and created a taxon list containing all recognized anuran species at that time (n=7,266). We then generated a marker search-terms file that included all 314 of these markers.

We created marker-specific FASTA files in SuperCRUNCH using *Parse_Loci.py*. We then performed sequence-similarity filtering steps with *Cluster_Blast_Extract.py* for most markers to ensure the same target regions were obtained for alignment. This method: (i) creates sequence clusters using CD-HIT-EST (Li and Godzik 2006) at the 80% sequence identity threshold, (ii) performs BLASTn searches (dc-megablast) for all sequences using the largest sequence cluster as the reference database, and (iii) outputs sequences that are trimmed based on their merged BLAST hit coordinates (Portik and Wiens 2020). We used *Reference_Blast_Extract.py* to perform sequence-similarity filtering for markers with complex records (those containing the target region plus non-target sequence), which included all mtDNA

markers and both regions of *RAG1*. This method differs from *Cluster_Blast_Extract.py* only by requiring a user-supplied set of reference sequences to use as the BLAST reference database (vs. a cluster generated from the input sequences). For reference sequences, we used the vertebrate RAG1 and the anuran mtDNA reference sets available from SuperCRUNCH (https://github.com/dportik/SuperCRUNCH/tree/master/data/reference-sequence-sets). We used *Filter_Seqs_and_Species.py* to select one sequence per species per gene (-s oneseq) based on the longest available sequence (-f length), with a minimum sequence length requirement of 300 bp (-m 300). For protein-coding markers, we pre-processed all sequences using *Coding_Translation_Tests.py*, which identifies the correct reading frame of sequences, adjusts them to the first codon position, and ensures completion of the final codon. We then used the MACSE v2 translation aligner (Ranwez et al. 2018), which can align coding sequences with respect to their translation while accounting for multiple frameshifts or stop codons. For all other markers we used MAFFT (Katoh et al. 2002, 2005; Katoh and Standley 2013) with the automatic alignment algorithm selection option.

We counted the number of markers with representation for each species across the different sets of markers (legacy, NPCL, AHE, and UCE). For each marker set, we selected the species with the greatest amount of data to represent a given genus. Thus, each genus is represented by one terminal taxon, but does not necessarily have data for the same species for each marker (Supplementary File S3). Accession numbers for all GenBank sequences selected (legacy and NPCL markers) are provided in Supplementary File S4. We obtained data for one or more of the selected markers for 441 of 456 frog described genera (96.7%; AmphibiaWeb 2021).

We generated two major concatenated data matrices. One contained all markers except the UCE markers. We refer to this as the supermatrix dataset. The other combined this supermatrix with the UCE data. We refer to this combined UCE-supermatrix dataset as the gigamatrix. These concatenated matrices were generated in SuperCRUNCH, after the selected sequences for each marker were relabeled with genus names. The basic properties of the different datasets are given in Tables 2 and 3 and in Supplementary File S2, Fig. S1. All individual alignments and the gigamatrix (with partition files) are provided on an Open Science Framework project page: https://osf.io/fzw3x/.

Phylogenetic Analyses

The UCE data were analyzed using both concatenated maximum likelihood (ML) and gene-tree summary methods. For the combined supermatrix and gigamatrix datasets, we only performed concatenated ML analyses given that many genera had data for only a few genes. We chose RAxML for all ML analyses because RAxML was found to obtain better results than IQ-TREE (Nguyen et al. 2015) for phylogenomic datasets containing >200 taxa (Zhou et al. 2018).

For the UCE data, we used RAxML v8.2 (Stamatakis 2014) on the Cyberinfrastructure for Phylogenetic Research (CIPRES) Science Gateway (Miller et al. 2010). We performed 50 alternate runs on distinct starting trees to find the best-scoring ML tree and conducted 100 rapid bootstrap analyses. We used the standard GTR+CAT model (general-time-reversible model with the CAT approximation of the gamma distribution of rates among sites, which is considered the optimal model in RAxML for very large datasets; Stamatakis 2006). We did not partition the UCE data because vertebrate UCE loci are often not protein-coding, and partition schemes are therefore unresolved (but see Tagliacollo and Lanfear 2018). Furthermore, as we describe below, using a large number of partitions was simply not practical for these datasets. We also found little difference between trees from partitioned and unpartitioned analyses.

We conducted species-tree analyses of the UCE data using a gene-tree summary method, ASTRAL-III (Mirarab et al. 2014; Mirarab and Warnow 2015; Zhang et al. 2017). A gene tree was constructed for each UCE using RAxML v8.2. Given the large number of UCEs, we used the relatively fast GTR+CAT model, with 10 replicate searches per gene. The complete set of gene trees was used to infer a species tree using ASTRAL-III. Branch support was assessed using local posterior probabilities (LPP), which are computed from gene-tree quartet frequencies (Sayyari and Mirarab 2016). We also visualized the level of congruence among gene trees on each branch of the UCE species-tree. Specifically, we used ASTRAL-III version 5.7.8 to output quartet support values on the species tree. These values represent the percentage of gene tree quartets that agree with each node in the species tree (Sayyari and Mirarab 2016). The results generally show that congruence between genes is greater on longer branches (i.e., in the concatenated ML tree), as expected from theory and previous studies (e.g., Wiens et al. 2008).

We used PartitionFinder2 (Lanfear et al. 2016) to identify the best partitioning scheme for our supermatrix and gigamatrix datasets. It was not possible to run PartitionFinder2 on the gigamatrix (combined UCE+supermatrix), even with greatly reduced taxon sampling and when

treating the UCEs as a single concatenated locus. Therefore, we analyzed the supermatrix dataset and extended these results to the gigamatrix, treating the UCEs as a single partition.

We performed two partitioning analyses. For the first analysis, the input partitions consisted of separate partitions for each codon position in each gene (with a single partition for non-protein coding genes, like 12S and 16S). For the second analysis, there was a single partition per gene. The best-fit partitioning schemes for these analyses had 916 and 225 partitions, respectively (Supplementary File S6). The GTR+gamma model was selected as the best RAxML model for most partitions (>90%) recovered in both analyses. Given that only one model type can be specified across each partition in RAxML, we selected GTR+gamma to represent all partitions. Running each of these partitioned datasets was computationally intractable on CIPRES. Specifically, the analyses failed in the first few minutes.

We therefore used a simpler partitioning scheme for the gigamatrix that contained a total of eight partitions: a partition for each of the three codon positions in the nuclear protein-coding genes, a partition for each of the three codon position in the mitochondrial protein-coding genes, a single partition for mitochondrial ribosomal RNA (including 12S and 16S), and one partition consisting of the UCEs (Supplementary File S6). We used seven partitions for the supermatrix (identical but with the exclusion of the UCEs). We then performed partitioned analyses of the supermatrix and gigamatrix datasets.

Given the large size of the gigamatrix we performed an initial investigation of the computational resources required. We first ran the dataset without partitions, using the GTR+CAT model with 10 replicate searches for the optimal tree.

For the gigamatrix, we conducted 10 alternate runs on distinct starting trees to find the best-scoring ML tree. To complete the analysis within CIPRES run-time allotments, we performed two separate analyses involving 5 alternate runs on distinct starting trees, with a final branch-length optimization on the best tree found in each analysis. Due to limitations in computational resources, our search for the best tree was not extensive. To address this potential shortcoming, we calculated the pairwise distance of Robinson and Foulds (1981) for the 10 optimized trees to determine their overall topological similarity. We then conducted four separate bootstrapping analyses of 25 bootstraps (to allow completion within CIPRES run-time allotments). These replicates were then combined manually to obtain a total of 100 rapid bootstrap replicates.

In addition to bootstrap analyses, for the gigamatrix tree we also calculated gene concordance factors (gCF) and site concordance factors (sCF) using IQ-TREE2 (Nguyen et al. 2015; Minh et al. 2020). The gCF is based on the proportion of loci that are consistent with a particular branch among the loci that are decisive for that branch (i.e., they could contain that branch). By contrast, the sCF is the average proportion of sites that are decisive for a given branch that are concordant with that branch. The latter is calculated at the per-site level, rather than whole-alignment level (i.e., it is performed with quartet approaches). We also included a summary of the number of decisive genes and sites for each branch (gN and sN; Minh et al. 2020). This was especially helpful for visualizing the data associated with each branch, given the variation in gene sampling among terminal taxa.

We analyzed the supermatrix in a similar fashion, using a partitioned analysis and the GTR+gamma model assigned to all seven partitions (see above). However, use of the GTR+gamma model required only ~1 Gb memory for the supermatrix. Therefore, we were able to conduct a more thorough search (50 alternate runs on distinct starting trees) to look for the best-scoring ML tree.

For the gigamatrix dataset (combined UCE+supermatrix), we were interested in identifying potential rogue taxa. These are taxa that are highly unstable in their phylogenetic placement and can reduce branch support in parts of the tree because of their instability. We used RogueNaRok (Aberer et al. 2013) to perform these analyses, using the best-scoring ML tree and bootstrap trees as inputs. The set of rogue taxa (*n*=29) was removed from the gigamatrix, and we ran an additional concatenated ML analysis with RAxML with 100 rapid bootstraps.

Time-calibrated Phylogeny

We estimated divergence times for the gigamatrix trees. We used penalized likelihood implemented with treePL (Sanderson, 2001; Smith and O'Meara, 2012). This is a standard approach for estimating divergence times for large trees. It is particularly advantageous here in that it used the maximum-likelihood branch lengths already calculated, rather than re-estimating these across thousands of loci. We removed non-amphibian outgroups prior to conducting divergence-time estimation. We conducted a thorough analysis in treePL during two optimization phases, one for search settings and one for smoothing parameter identification. The "thorough" setting ensures the analysis continue to iterate until convergence of log likelihoods is

observed. We tested the following standard smoothing values in treePL: 0.1, 1, 10, 100, 1,000, and 10,000. We conducted analyses on both the full gigamatrix tree and on the tree with the 29 rogue taxa excluded.

We used 16 fossil calibration points in the dating analyses. To find fossil calibration points we reviewed those used in recent large-scale time-calibrated phylogenies of amphibians (Feng et al. 2017; Jetz and Pyron 2018; Hime et al. 2021) and included other recent works on fossil frogs (see below). We used the geological time scale version 5.0 (Walker et al. 2018). Below, we list the fossil calibration points used.

The calibration points listed below were minimum ages with a single exception: we used a maximum value for Batrachia of 251.2 Mya, following Benton et al. (2015). This follows from the absence of crown lissamphibians from the Permian fossil record (Benton et al. 2015; Cannatella 2015).

1) Batrachia crown group (crown-group of frogs and salamanders). Feng et al. (2017) used a minimum date of 247.2 Mya for *Triadobatrachus massinoti*, following Cannatella (2015). This is a stem anuran from the Early Triassic (Carroll 1988; Rage and Roček 1989). Jetz and Pyron (2018) used 245 Mya. The minimum age for the beginning of the Early Triassic is 247.2 Mya.

Crown-group age of Batrachia: minimum=247.2 Mya; maximum=251.2 Mya

2) Alytoidea crown group (crown-group of Alytidae+Bombinatoridae). Feng et al. (2017) used *Iberobatrachus angelae* to set a minimum age of 125 Mya for the alytoid crown group. The phylogenetic analysis by Báez (2013) placed this taxon as the sister to *Discoglossus*, but did not show Alytidae as monophyletic (but instead as paraphyletic with respect to Bombinatoridae). We conservatively used this taxon to set the minimum age for the alytoid crown group. This taxon is from the Late Barremian. According to Walker et al. (2018) the minimum age for the Late Barremian is 125.0 Mya.

Crown-group age of Alytoidea: minimum=125.0 Mya

3) Alytidae. Fossils of the genus *Latonia* (Roček and Rage 2000) have been shown to be within crown-group Discoglossidae (Yuan et al. 2000) and are at least 23.0 Mya (Late Oligocene). In

the tree estimated here, *Latonia* and *Discoglossus* are sister taxa, and so we used these fossils to set the minimum age of the split between them.

Crown-group age of *Latonia* + *Discoglossus*: minimum=23.0 Mya.

4) Pipoidea crown group (crown-group of Pipidae+Rhinophrynidae). Feng et al. (2017) used the fossil *Rhadinosteus parvus* to set a minimum age for Pipanura (crown group of Pipoidea + Pelobatoidea + Neobatrachia) at 148.1 Mya, following Cannatella (2015). The analysis by Báez (2013) showed that *Rhadinosteus parvus* is more closely related to *Rhinophrynus* than *Pipa+Xenopus.* Therefore, we used this taxon to set the minimum age of Pipoidea (Rhinophrynidae+Pipidae) and not Pipanura (although equal-weights parsimony analyses by Gómez (2016) placed *Rhadinosteus* in a polytomy with other pipoids). *Rhadinosteus parvus* is from the Late Jurassic (Tithonian), and Cannatella (2015) reviewed evidence suggesting that the appropriate minimum age is 148.1 Mya (based on the age of the Brush Bush Formation where the taxon was found). Note that this is older than the minimum age of 127.2 Mya set for Pipoidea by Feng et al. (2017), using *Neusibatrachus wilferti* or *Cordicephalus* (125 Mya) used by Jetz and Pyron (2018).

Crown-group age of Pipoidea: minimum=148.1 Mya

5) Pipidae crown group. Feng et al. (2017) used the pipid fossil *Pachycentrata taqueti* to set a minimum age of 83.6 Mya for this family, following Cannatella (2015). Cannatella (2015) noted that the minimum age of the formation in which this fossil was found is 83.6 Mya, based on the age of the "In Beceten" Formation from the late Coniacian-Santonian (Upper Cretaceous). The minimum age of the Santonian is 83.6 Mya (Walker et al. 2018). Cannatella (2015) used a phylogenetic analysis to show that this taxon is within the crown-group of Pipidae.

Crown-group age of Pipidae: minimum=83.6 Mya

6) Scaphiopodidae crown group. We used a minimum age of 50.3 Mya, given a fossil scaphiopodid (*Scaphiopus guthriei*) from the Wind River formation (lower Eocene Wasatchian 50.3—55.4 Mya) that is more closely related to *Scaphiopus* than to *Spea* (Gao and Chen 2017). We assigned this taxon to the crown-group of Scaphiopodidae, which contains only two living genera (*Scaphiopus, Spea*).

Crown-group age of Scaphiopodidae: minimum=50.3 Mya

7) Clade of Pelobatidae and Megophryidae (crown group). The analysis of Chen et al. (2016) found *Gobiates spinari* nested within a clade containing Megophryidae + Pelobatidae. We used this taxon as a minimum calibration point for this clade. *Gobiates spinari* is from the Coniacian (Upper Cretaceous) of Uzbekistan (Roček 2008). According to Walker et al. (2018) the minimum age for the Coniacian is 86.3 Mya. Feng et al. (2017) used *Elkobatrachus brocki* with a minimum age of 46.1 Mya to date the crown group of Pelobatoidea, and the fossil pelodytid frog *Miopelodytes gilmorei* (minimum age of 38.9 Mya) following Henrici and Haynes (2006), to date the clade of Pelodytidae + Pelobatidae + Megophryidae. We did not use these two calibration points because the older age of *Gobiates spinari* for the younger clade Pelobatidae + Megophryidae makes them redundant.

Crown-group age of Pelobatidae+Megophryidae: minimum=86.3 Mya

8) Calyptocephalellidae (crown group). We used a minimum age of 61.7 Mya, given fossil *Calyptocephalella* (formerly *Caudiverbera*) from the Early Paleocene (65.5–61.7 Mya; Báez 2000). Feng et al. (2017) used the fossil *Calyptocephalella pichileufensis* to set a minimum age of 47.5 Mya for the Myobatrachoidea (Myobatrachidae + Calyptocephalellidae). However, given the taxon sampling here, this fossil should instead set the minimum age for the split between the two genera of Calyptocephalellidae (*Calyptocephalella* and *Telmatobufo*). Furthermore, we used the older fossil calibration point for this genus.

Crown-group age of Calyptocephalellidae: minimum=61.7 Mya.

9) Myobatrachidae (crown group). We used a minimum age of 54.6 Mya, given fossils assigned to the extant myobatrachid genus *Lechriodus* (Sanchiz 1998; Evans et al. 2008). We assumed that the precise phylogenetic placement of this taxon was not known, and so we merely assigned it to the Myobatrachidae crown group.

Crown-group age of Myobatrachidae: minimum=54.6 Mya

10) Hylidae: Pelodryadinae stem group. Pelodryadine fossils are present from the late Oligocene of Australia (23.0–28.4 Mya; Sanchiz 1998). Therefore, we set the minimum age of the split between Pelodryadinae and its sister group (Phyllomedusinae) to be at least 23.0 Mya.

Stem-group age of Pelodryadinae: minimum=23.0 Mya

11) Hylidae: crown-group age of *Acris* + *Pseudacris*. Given the fossil taxon *Acris barbouri* from the Miocene (Hemingfordian: 16.3–20.6 Mya) of Florida (Holman, 2003; Marjanovic and Laurin, 2007), we set a minimum age of 16.3 Mya on the split between the genera *Acris* and *Pseudacris*.

Crown-group age of *Acris* + *Pseudacris*: minimum=16.3 Mya

12) Eleutherodactylidae: Eleutherodactylinae crown group. For the age of this clade, we used the minimum age in the Oligocene of the San Sebastian Formation in Puerto Rico (~29.5 Mya) from which a fossil referred to as *Eleutherodactylus* was described by Blackburn et al. (2020). Poinar and Cannatella (1987) described an amber fossil of *Eleutherodactylus* from the La Toca formation (Dominican Republic) as being from the Eocene, but these deposits have been more recently interpreted as Miocene in age. The content of *Eleutherodactylus* has changed considerably over time, but *Eleutherodactylus* (sensu stricto) is the only genus of terraranans that currently occurs on Puerto Rico (and other islands of the Greater Antilles). Therefore, we assigned this fossil to the genus *Eleutherodactylus* and used it as a minimum age constraint on the crown-group age of the subfamily (which also contains the mainland genus *Diasporus*).

Crown-group age of Eleutherodactylinae (*Diasporus*+*Eleutherodactylus*): minimum=29.5 Mya

13) Crown Bufonidae. We used a minimum age of 56.0 Mya, given fossils of putative *Bufo* (sensu lato) from the late Paleocene (56.0–59.2 Mya; Báez 2000; Báez and Nicoli 2004; Walker et al. 2018). We considered these fossils to be within the crown group of Bufonidae, given that they were considered to be “unquestionable” remains of *Bufo,* sensu lato (Báez and Nicoli 2004), which represents a subclade within the bufonid crown group.

Crown age of Bufonidae: minimum=56.0 Mya

14) Stem *Rhinella*. Báez and Nicoli (2004) identified bufonid fossils that they considered to be closely related to extant *Rhinella arenarum*, from the late Oligocene (specifically 29.4–25.5 Mya). We used these fossils to set the minimum age for the split between *Rhinella* and its sister group. In the gigamatrix tree, *Rhinella* is sister group to a large clade including most bufonid genera that were formerly within *Bufo* (e.g., *Anaxyrus, Incillius, Duttaphrynus*) and their close relatives (e.g., *Nectophrynoides, Pedostibes*).

Stem age of *Rhinella*: minimum=25.5 Mya

15) Stem Ptychadenidae. Following Feng et al. (2017), we constrained the minimum age of the node uniting Ptychadenidae and Phrynobatrachidae based on a fossil of Ptychadenidae that was well-dated to 24.5–25.5 Mya (Blackburn et al. 2015). We used a date of 24.5 Mya to constrain the minimum age of the split between Ptychadenidae and its putative sister group in our tree, Phrynobatrachidae.

Crown age of Ptychadenidae+Phrynobatrachidae: minimum=24.5 Mya.

16) Crown Pyxicephalidae. We set a minimum age for the crown group of Pyxicephalidae based on the fossil taxon *Thaumastosaurus* (Lemierre et al. 2021). The oldest remains of this genus are from the late middle Eocene of Europe, dated to ~39.5 Mya (Lemierre et al. 2021). Lemierre et al. (2021) considered this taxon to belong to the subfamily Pyxicephalinae, based on their phylogenetic analyses (e.g., their Fig. 15). However, to be conservative we used it to date the crown-group age of the family.

Crown age of Pyxicephalidae: minimum=39.5 Mya

Supplementary Results

Monophyly of Subfamilies

The gigamatrix tree (Fig. 3) also supported the monophyly of most subfamilies in which two or more genera were sampled. Following the taxonomy of AmphibiaWeb (2020; November), these subfamilies included those in: Alytidae (Discoglossinae), Megophryidae (Leptobrachiinae, Megophryinae), Microhylidae (Otophryninae, Gastrophryninae, Scaphiophryninae, Cophylinae, Microhylinae, Asterophryninae), Pyxicephalidae (Pyxicephalinae, Cacosterninae), Ceratobatrachidae (Ceratobatrachinae), Dicroglossidae (Occidozyginae, Dicroglossinae), Mantellidae (Laliostominae), Rhacophoridae (Rhacophorinae), Myobatrachidae (Limnodynastinae, Myobatrachinae), Hylidae (Pelodryadinae, Phyllomedusinae, Hylinae), Dendrobatidae (Hyloxalinae, Aromobatinae, Colostethinae), Eleutherodactylidae (Phyzelaphryninae, Eleutherodactylinae), Strabomantidae (Holoadeninae), Centrolenidae (Hyalinobatrachinae, Centroleninae), and Leptodactylidae (Paratelmatobiinae, Leptodactylinae). These results suggest that highly incomplete taxa were also correctly placed in clades within families, and not merely placed in the correct families.

We found four non-monophyletic subfamilies in the gigamatrix tree (Fig. 3). In Mantellidae, we did not support monophyly of Mantellinae, given the weakly supported placement of the mantelline *Tsingymantis* with Laliostominae. *Tsingymantis* is represented by 6 markers here and is 99.88% incomplete. In Dendrobatidae we supported monophyly of Aromobatinae, Colostethinae, and Hyloxalinae, but not Dendrobatinae. Non-monophyly of Dendrobatinae was not related to a single misplaced taxon: instead a strongly supported clade of *Excidobates, Andinobates*, and *Ranitomeya* was weakly placed with Hyloxalinae and not with other dendrobatine genera (but note that Dendrobatinae is monophyletic in the supermatrix tree). In Leptodactylidae, we did not support monophyly of Leiuperinae, given that *Pseudopaludicola* is weakly placed with Paratelmatobiinae. *Pseudopaludicola* is represented by 12 markers and is 99.76% incomplete. In Strabomantidae, Pristimantinae is non-monophyletic given the placement of *Tachiramantis* with Craugastoridae. *Tachiramantis* is represented by 4 markers and is 99.89% incomplete. Importantly, in the supermatrix tree, *Tsingymantis* does not group with other mantellines, *Pseudopaludicola* is placed with Paratelmatobiinae, and *Tachiramantis* is placed with Craugastoridae. Therefore, non-monophyly of Mantellinae, Leiuperinae, and Pristimantinae cannot be attributed to extensive missing data arising from the assembly of the gigamatrix.

Furthermore, these genera are not especially incomplete (i.e., 49 genera have fewer than 6 markers, with 12 genera having only 1 or 2 markers).

Analysis of Branch Lengths

There was also considerable heterogeneity in branch lengths among the terminal taxa in this tree (Fig. 3). We found a significant positive correlation between terminal branch lengths and missing data among these terminal taxa (tau=0.27, $P<0.0001$; using non-parametric Kendall correlation in R; data in Supplementary File S3). Considering only taxa with a predominance of nuclear markers (UCE, AHE, and/or NPCL, not those with legacy-data only), the mean terminal branch length among the 234 taxa was 0.0370 substitutions/site. Among these taxa, there was a weak negative correlation, such that taxa with more missing data had shorter branch lengths (tau=-0.09, $P=0.0417$). The percentage of missing data for these taxa ranged from 40.7% to 96.7%. Considering only species that had legacy data alone (i.e., with more equivalent numbers of mitochondrial and nuclear markers), the mean terminal branch length among the 207 taxa was 0.0570. Among these legacy-only taxa, there was a significant positive, correlation between missing data and branch lengths (tau=0.16, $P=0.0007$). Yet, the percentage of missing data among these taxa ranged only from 97.19% to 99.98%. We suggest that these patterns are explained (at least in part) by longer branch lengths associated with faster evolutionary rates in the mitochondrial markers, which predominate among the highly incomplete legacy-only taxa. For example, those taxa with only 1 or 2 markers in total had only mitochondrial markers and each had >99.9% missing data. Thus, the heterogeneity in branch lengths seems to be related to heterogeneity in the type of data present, not the amount of missing data alone.

Comparison To Other Recent Studies

Here, we compare our main trees to those in other recent studies. Specifically, we compare our concatenated UCE-only tree (Fig. 2) and combined UCE-supermatrix (gigamatrix) tree to three other recent estimates: two based on phylogenomic datasets (Feng et al. 2017; Hime et al. 2021) and the supermatrix study of Jetz and Pyron (2018). For brevity, we focus primarily on relationships among families and other higher-level relationships. Furthermore, the phylogenomic studies have limited sampling within families.

We first compare the overall congruence of our tree with these trees quantitatively, then address specific clades. Specifically, we compared the number of nodes shared between each of these trees and our gigamatrix tree (Fig. 3), for relationships among families. Some families were missing in the tree of Hime et al. (2021) and the phylogenomic tree of Feng et al. (2017; their Fig. 1). We excluded from these comparisons those nodes in the gigamatrix tree in which directly relevant families were absent in the other trees (e.g., a clade consisting of families A+B in our tree when B is absent in the other tree), but we included nodes that were potentially congruent (e.g., a clade consisting of (A(B,C)) in our tree when B is absent in the other tree). For the supermatrix tree of Jetz and Pyron (2018), 33 of 53 nodes were concordant (62.3%). For Feng et al. (2017), this depended on the tree used. For their tree including all families (their Fig. 2), 35 of 53 nodes were concordant (66.0%). Much of the discordance involved the placement of Cycloramphidae, Micrixalidae, and Ranixalidae, which lacked full phylogenomic data. Considering their smaller, strictly phylogenomic dataset, six nodes were not comparable due to missing taxa, and 43 of 47 were concordant (91.5%). For the tree of Hime et al. (2021), four nodes were not comparable, and 44 of 49 were concordant (89.8%). Using a Chi-squared test in R (which does not assume a normal distribution), there was a significantly higher proportion of nodes shared between the gigamatrix tree and these phylogenomic trees than between the gigamatrix tree and the supermatrix tree of Jetz and Pyron (2018): supermatrix vs. Hime et al. (2021), P=0.0012; supermatrix vs. Feng et al. (2017; Fig. 1), P=0.0006. Overall, we found that higher-level relationships in the gigamatrix tree were most concordant with those from other, strictly phylogenomic datasets (e.g., Feng et al. 2017 [Fig. 1]; Hime et al. 2021), and not from supermatrix datasets (e.g., Jetz and Pyron 2018). This result parallels the comparison of our own gigamatrix, supermatrix, and phylogenomic trees.

Here, we describe the specific areas of congruence and discordance with these three previous higher-level studies. Our results agree with previous studies regarding the major clades of frogs. All recent studies agree on the relationships among Leiopelmatoidea (Ascaphidae+Leiopelmatidae), Discoglossoidea (Alytidae, Bombinatoridae), Pipoidea (Rhinophrynidae, Pipidae), Pelobatoidea (Scaphiopodidae, (Pelodytidae, (Pelobatidae, Megophryidae))), and Neobatrachia. Our gigamatrix tree agrees with other recent studies in that Heleophrynidae is sister to all other neobatrachians (although we lacked UCE data for

Heleophrynidae) and that the clade Sooglossidae+Nasikabatrachidae is sister to Ranoidea (but we lacked UCE data for both families).

Our UCE and gigamatrix trees place Microhylidae as sister to all other Ranoidea (Figs. 2, 3A). This result is supported by other recent studies (Feng et al. 2017; Hime et al. 2021), except for Jetz and Pyron (2018), who placed Microhylidae as sister to Afrobatrachia instead. Within Afrobatrachia, our results and other recent studies agree on the relationships: ((Hemisotidae, Breviciptidae), (Hyperoliidae, Arthroleptidae)).

Relationships within Natatanura (all other ranoids) show considerable discordance, however. Our UCE data alone place the African family Pyxicephalidae as sister to all other Natatanura, including a clade of mostly African families ((Conrauidae, Petropedetidae), (Odontobatrachidae, (Ptychadenidae, Phrynobatrachidae))) and a clade of mostly Asian families: ((Ranixalidae, Ceratobatrachidae), ((Dicroglossidae, (Ranidae, (Mantellidae, Rhacophoridae))))). These relationships are generally strongly supported (Fig. 2). The gigamatrix (combined UCE-supermatrix) tree supports somewhat different relationships, which are generally weakly supported (Fig. 3B). The South Asian family Micrixalidae (absent in the UCE dataset) is sister to all other Natatanura, and the African families do not form a monophyletic group. The trees of Feng et al. (2017) and Hime et al. (2021) both support a clade of primarily African families: ((Odontobatrachidae, (Ptychadenidae, Phrynobatrachidae)), (Pyxicephalidae, (Conrauidae, Petropedetidae))). All these trees (including the gigamatrix tree) agree that Conrauidae and Petropedetidae are sister taxa and that Odontobatrachidae is the sister group to the clade of Ptychadenidae and Phrynobatrachidae. The tree of Jetz and Pyron (2018) is more discordant: it also suggests that African families form a paraphyletic group with respect to the primarily Asian families, but the overall ranoid relationships are quite different.

Among the primarily Asian families in Natatanura, the relationships found in the UCE-only and gigamatrix trees (Figs. 2, 3B) are generally concordant with those of Hime et al. (2021) and the phylogenomic tree of Feng et al. (2017; their Fig. 1). The gigamatrix tree shows: ((Ceratobatrachidae, (Nyctibatrachidae, Ranixalidae)), ((Dicroglossidae, (Ranidae, (Mantellidae, Rhacophoridae)))). However, our UCE-only tree lacked data for Nyctibatrachidae (Fig. 2), Hime et al. (2021) lacked data for Ranixalidae, and Feng et al. (2017; Fig. 1) lacked data for both. Jetz and Pyron (2018) supported somewhat different relationships: ((Nyctibatrachidae, Ceratobatrachidae), (((Ranixalidae, Dicroglossidae), (Ranidae, (Mantellidae, Rhacophoridae)))).

Furthermore, the concatenated tree of Feng et al. (2017; their Fig. 2) supported quite different relationships, and placed Nyctibatrachidae as the sister group to Ranidae, and placed Micrixalidae + Ranixalidae as the sister group to Mantellidae + Rhacophoridae.

Most studies agree that Myobatrachidae and Calyptocephalellidae form a clade that is the sister group to Hyloidea, including our gigamatrix tree (Fig. 3C) and the analyses of Feng et al. (2017), Jetz and Pyron (2018), and Hime et al. (2021). Surprisingly, our analysis of UCE data alone suggest that this grouping is paraphyletic with respect to Hyloidea (Fig. 2).

Overall, hyloid relationships (Figs. 2, 3C) were broadly similar between our results and those of Feng et al. (2017; Fig. 1) and Hime et al. (2021). These analyses agree that Rhinodermatidae is sister to all other Hyloidea, that a clade of southern South American frogs (Neoaustrarana; Streicher et al. 2018) is sister to the remaining Hyloidea (Cornucopirana), and that Telmatobiidae is sister to other members of Cornucopirana (Streicher et al. 2018). The remaining hyloids generally belong to the groups Amazorana and Commutabirana (Streicher et al. 2018). Commutabirana also includes Terraranae. However, the tree of Jetz and Pyron (2018) is broadly discordant with our trees and others within Hyloidea.

There is also disagreement between analyses within some of these groups. Within Neoaustrarana (Fig. 2), the UCE-only data show strong support for the relationships: (Alsodidae, (Batrachylidae, (Cycloramphidae, Hylodidae). The gigamatrix tree (Fig. 3C) shows: ((Cycloramphidae, Hylodidae), (Alsodidae, Batrachylidae)). Hime et al. (2021) found the relationships: (Cycloramphidae, (Hylodidae, (Alsodidae, Batrachylidae)). The concatenated likelihood analysis of Feng et al. (2017) did not support the monophyly of Neoaustrarana, because their concatenated analysis placed Cycloramphidae in Commutabirana. Jetz and Pyron (2018) did support monophyly of Neoaustrarana, and the relationships: (Cycloramphidae, (Batrachylidae, (Alsodidae, Hylodidae))).

Within the predominantly South American clade Amazorana (Figs. 2, 3C), our UCE-only and gigamatrix trees support the relationships: (Hylidae, (Hemiphractidae, Ceratophryidae)). Feng et al. (2017) placed Ceratophryidae as sister to the other two families, whereas Hime et al. (2021) placed Hemiphractidae as sister to Hylidae and Ceratophryidae. Jetz and Pyron (2018) did not place these three families together.

Relationships were more congruent within Commutabirana. Our UCE-only and gigamatrix (combined UCE-supermatrix) data (Figs. 2, 3D) support the relationships:

(Dendrobatidae, (Terraranae, ((Leptodactylidae, (Allophrynidae, Centrolenidae), (Bufonidae, Odontophrynidae))))). Hime et al. (2021) found the same relationships, but they did not include Allophrynidae. Feng et al. (2017) supported Dendrobatidae and Terraranae as successive sister taxa to other members of this group (as did we), but other relationships were somewhat different, and they included Cycloramphidae in this group as well. Jetz and Pyron (2018) did not support monophyly of Commutabirana, but they did support a clade including: ((Bufonidae, Odontophrynidae), (Leptodactylidae (Allophrynidae, Centrolenidae))). This is congruent with our results.

Finally, there was general agreement among these studies on higher-level relationships within Terraranae. The UCE data alone (Fig. 2) supported the relationships: (Brachycephalidae (Eleutherodactylidae (Craugastoridae, Strabomantidae))). The gigamatrix dataset (Fig. 3D) and those of Feng et al. (2017) and Hime et al. (2021) supported similar relationships, but with Ceuthomantidae included as sister to all other terraranans. However, the tree of Jetz and Pyron (2018) differed in placing Eleutherodactylidae and Brachycephalidae as sister taxa.

Divergence Times

The best smoothing value for the treePL analysis of the full gigamatrix tree was 1, based on the cross-validation analysis. We also conducted an analysis with the 29 rogue taxa excluded (where the best smoothing value was 1,000). Note that both smoothing values were intermediate (neither the smallest nor largest examined), suggesting that the optimal value was indeed within the range examined. The trees are shown in Supplementary Files S11 and S12 (all taxa vs. no rogues) and are available in newick format in Supplementary Files S18 and S19.

Compared to previous studies (Tables S4, S5), our divergence times were similar for shallow nodes in the phylogeny, but were often younger for deeper nodes. In particular, the origin of Leiopelmatoidea (Ascaphidae [North America] + Leiopelmatidae [New Zealand]) was estimated to be substantially younger (106 Mya, for all taxa) than previous studies (Feng et al. 2017; Jetz and Pyron 2018; Hime et al. 2021). Nevertheless, a Middle Cretaceous origin of Leiopelmatoidea is plausible and a hypothesis with some precedent. Both geological evidence and estimates from other vertebrate timetrees (e.g., moas from New Zealand and their American relatives, ~80 Mya) suggest that continental connections between New Zealand and the Americas were present in the Late Cretaceous (Cooper et al. 2001; Kula et al. 2007). Indeed, some

amphibian biologists have previously used this time frame to calibrate the minimum divergence time of Leiopelmatoidea to the Late Cretaceous (Roelants and Bossuyt 2005).

References

Allen JM, Huang DI, Cronk QC, Johnson KP. 2015. aTRAM automated target restricted assembly method a fast method for assembling loci across divergent taxa from next-generation sequencing data. *BMC Bioinform.* **16**:98.

Allen JM, LaFrance R, Folk RA, Johnson KP, Guralnick RP. 2018. aTRAM 2.0: an improved, flexible locus assembler for NGS data. *Evol Bioinform.* **14**:1–4.

Báez AM, Moura GJB, Gomez RO. 2009. Anurans from the Lower Cretaceous Crato Formation of northeastern Brazil: implications for the early divergence of neobatrachians. *Cretac Res.* **30**:829–846.

Báez AM, Nicoli L. 2004. Bufonid toads from the late Oligocene beds of Salla, Bolivia. *J Vertebr Paleontol.* **24**:73–79.

Báez AM. 2000. Tertiary anurans from South America. Pp. 1388–1401 in H. Heatwole and R. L. Carroll (eds.) Amphibian biology. Vol. 4. Surrey Beatty, Chipping Norton, Australia.

Báez AM. 2013. Anurans from the Early Cretaceous Lagerstätte of Las Hoyas, Spain: New evidence on the Mesozoic diversification of crown-clade Anura. *Cretac Res.* **41**:90–106.

Bankevich A, Nurk S, Antipov D, Gurevich AA, Dvorkin M, Kulikov AS, Lesin VM, Nikolenko SI, Pham S, Prjibelski AD, *et al.* 2012. SPAdes: A new genome assembly algorithm and its applications to single-cell sequencing. *J Comput Biol.* **19**:455–477.

Benton MJ, Donoghue PCJ, Asher RJ, Friedman M, Near TJ, Vinther J. 2015. Constraints on the timescale of animal evolutionary history. *Palaeont Electron.* **18.1.1FC**:1–106.

Blackburn DC, Keeffe RM, Vallejo-Pareja MC, Vélez-Juarbe J. 2020. The earliest record of Caribbean frogs: a fossil coquí from Puerto Rico. *Biol Lett.* **16**:20190947.

Blackburn DC, Roberts EM, Stevens NJ. 2015. The earliest record of the endemic African frog family Ptychadenidae from the Oligocene Nsungwe Formation of Tanzania. *J Vertebr Paleontol.* **35**:e907174.

Bolger AM, Lohse M, Usadel B. 2014. Trimmomatic: a flexible trimmer for Illumina sequence data. *Bioinformatics* **30**:2114–2120.

Cannatella DC. 2015. *Xenopus* in space and time: fossils, node calibrations, tip-dating, and

paleobiogeography. *Cytogenet Genome Res* **145**, 283–301.
Capella-Gutiérrez S, Silla-Martínez JM, Gabaldón T. 2009. trimAl: a tool for automated alignment trimming in large-scale phylogenetic analyses. *Bioinformatics* **25:**1972–1973.
Chen J, Bever GS, Yi HY, Norell MA. 2016. A burrowing frog from the late Paleocene of Mongolia uncovers a deep history of spadefoot toads (Pelobatoidea) in East Asia. *Sci Rep*. **6**:19209.
Chen S, Zhou Y, Chen Y, Gu J. 2018. Fastp: an ultra-fast all-in-one FASTQ preprocessor. *Bioinformatics* **34**:i884–i890.
Cooper A, Lalueza-Fox C, Anderson S, Rambaut A, Austin J, Ward R. 2001. Complete mitochondrial sequences of two extinct moas clarify ratite evolution. *Nature* **409**:704–707.
Denton RD, Morales AE, Gibbs HL. 2018. Genome-specific histories of divergence and introgression between an allopolyploid unisexual salamander lineage and two ancestral sexual species. *Evolution* **72:**1689–1700.
Faircloth BC. 2016. PHYLUCE is a software package for the analysis of conserved genomic loci. *Bioinformatics* **32:**786–788.
Feng YJ, Blackburn DC, Liang D, Hillis DM, Wake DB, Cannatella DC, Zhang P. 2017. Phylogenomics reveals rapid, simultaneous diversification of three major clades of Gondwanan frogs at the Cretaceous-Paleogene boundary. *Proc Natl Acad Sci USA* **114**:E5864–E5870.
Gao KQ, Chen J. 2017. A new crown-group frog (Amphibia: Anura) from the Early Cretaceous of northeastern Inner Mongolia, China. *Am Mus Novt.* **2017***:*1–39.
Gómez RO.2016. A new pipid frog from the Upper Cretaceous of Patagonia and early evolution of crown-group Pipidae. *Cretac Res.* **62**:52–64.
Harris RS. 2007. Improved pairwise alignment of genomic DNA. Ph.D. Thesis, The Pennsylvania State University.
Hime PM, Lemmon AR, Lemmon ECM, Prendini E, Brown JM, Thomson RC, Kratovil JD, Noonan BP, Pyron RA, Peloso PLV. *et al.* 2021. Phylogenomics reveals ancient gene tree discordance in the amphibian tree of life. *Syst Biol.* **70:**49–66.
Holman JA. 2003. Fossil Frogs and Toads of North America. (Indiana University Press, Bloomington and Indianapolis, Indiana).

Jetz W, Pyron RA. 2018. The interplay of past diversification and evolutionary isolation with present imperilment across the amphibian tree of life. *Nat Ecol Evol*. **2**:850.

Katoh K, Standley DM. 2013. MAFFT multiple sequence alignment software version 7: improvements in performance and usability. *Mol Biol Evol.* **30**:722–780.

Katoh K, Kuma K, Toh H, Miyata T. 2005. MAFFT version 5: improvement in accuracy of multiple sequence alignment. *Nucleic Acids Res.* **33**:511–518.

Katoh K, Misawa K, Kuma K, Miyata T. 2002. MAFFT: a novel method for rapid multiple sequence alignment based on fast Fourier transform. *Nucleic Acids Res.* **30**:3059–3066.

Kent WJ, Sugnet CW, Furey TS, Roskin KM, Pringle TH, Zahler AM, Haussler D. 2002. The human genome browser at UCSC. *Genome Res.* **12:**996–1006.

Kula J, Tulloch A, Spell TL, Wells ML. 2007. Two-stage rifting of Zealandia-Australia-Antarctica: Evidence from ^{40}Ar/^{39} Ar thermochronometry of the Sisters shear zone, Stewart Island, New Zealand. *Geology* **35**:411–414.

Laloy F, Rage J-C, Evans SE, Boistel R, Lenoir N, Laurin M. 2013. A re-interpretation of the Eocene anuran *Thaumastosaurus* based on microCT examination of a 'mummified' specimen. *PLoS One* **8**:e74874

Lanfear R, Frandsen PB, Wright AM, Senfeld T, Calcott B. 2016. PartitionFinder2: new methods for selecting partitioned models of evolution for molecular and morphological phylogenetic analyses. *Mol Biol Evol.* **34**:772–773.

Lemierre A, Folie A, Bailon S, Robin N, Laurin M. 2021. From toad to frog, a CT-based reconsideration of Bufo servatus, an Eocene anuran mummy from Quercy (France). *J Vertebr Paleontol.* **41**:e1989694

Li H, Durbin R. 2009. Fast and accurate short read alignment with Burrows-Wheeler Transform. *Bioinformatics* **25**:1754–1760.

Li H, Handsaker B, Wysoker A, Fennell T, Ruan J, Homer N, Marth G, Abecasis G, Durbin R, and 1000 Genome Project Data Processing Subgroup. 2009. The Sequence alignment/map (SAM) format and SAMtools. *Bioinformatics* **25**:2078–2079.

Li W, Godzik A. 2006. Cd-hit: a fast program for clustering and comparing large sets of protein or nucleotide sequences. *Bioinformatics* **22**:1658–1659.

Marjanovic D, Laurin M. 2007. Fossils, molecules, divergence times, and the origin of Lissamphibians. *Syst Biol.* **56**:369–388.

Miller MA, Pfeiffer W, Schwartz T. 2010. Creating the CIPRES Science Gateway for inference of large phylogenetic trees. In Proceedings of the Gateway Computing Environments Workshop (GCE), 14 Nov. 2010, New Orleans, LA pp 1–8.

Minh BQ, Hahn MW, Lanfear R. 2020. New methods to calculate concordance factors for phylogenomic datasets. *Mol Biol Evol.* **37**:2727–2733.

Mirarab S, Warnow T. 2015. ASTRAL-II: coalescent-based species tree estimation with many hundreds of taxa and thousands of genes. *Bioinformatics* **31**:i44–i52.

Mirarab S, Reaz R, Bayzid MS, Zimmermann T, Swenson MS, Warnow T. 2014. ASTRAL: genome-scale coalescent-based species tree estimation. *Bioinformatics* **30**:i541–i548.

Newman CE, Austin CC. 2016. Sequence capture and next-generation sequencing of ultraconserved elements in a large-genome salamander. *Mol Ecol.* **25**:6162–6174.

Nguyen LT, Schmidt HA, von Haeseler A, Minh BQ. 2015. IQ-TREE: a fast and effective stochastic algorithm for estimating maximum-likelihood phylogenies. *Mol Biol Evol.* **32:**268–274.

Poinar GO, Cannatella DC. 1987. An upper Eocene frog from the Dominican Republic and its implication for Caribbean biogeography. *Science* **237**:1215–1216.

Ranwez V, Douzery EJP, Cambon C, Chantret N, Delsuc F. 2018. MACSE v2: toolkit for the alignment of coding sequences accounting for frameshifts and stop codons. *Mol Biol Evol.* **35:**2582–2584.

Roček Z. 2008. The Late Cretaceous frog *Gobiates* from Central Asia: its evolutionary status and possible phylogenetic relationships. *Cretac Res.* **29**:577–591.

Roček Z, Rage J-C. 2000. Tertiary Anura of Europe, Africa, Asia, North America, and Australia. Pp. 1332–1387 in H. Heatwole and R. L. Carroll (eds.) Amphibian biology. Vol. 4. Surrey Beatty, Chipping Norton, Australia.

Roelants K, Bossuyt F. 2005. Archaeobatrachian paraphyly and Pangaean diversification of crown-group frogs. *Syst Biol.* **54**:111–126.

Sanchiz FB. 1998. Encyclopedia of Paleoherpetology, Part 4, Salientia.

Sanderson MJ, McMahon MM, Steel M. 2011. Terraces in phylogenetic tree space. *Science* **333**:448–450.

Sayyari E, Mirarab S. 2016. Fast coalescent-based computation of local branch support from quartet frequencies. *Mol Biol Evol.* **33:**1654–1668.

Shen XX, Liang D., Feng YJ, Chen MY, Zhang P. 2013. A versatile and highly efficient toolkit including 102 nuclear markers for vertebrate phylogenomics, tested by resolving the higher level relationships of the Caudata. *Mol Biol Evol.* **30:**2235–2248.

Smith SA, O'Meara BC. 2012. treePL: divergence time estimation using penalized likelihood for large phylogenies. *Bioinformatics* **28:**2689–2690.

Stamatakis A. 2006. Phylogenetic models of rate heterogeneity: a high performance computing perspective. In: Proceedings of IPDPS2006, HICOMB Workshop, Proceedings on CD, IEEE, Rhodos, Greece.

Tagliacollo VA, Lanfear R. 2018. Estimating improved partitioning schemes for ultraconserved elements. *Mol Biol Evol.* **35:**1798–1811.

Walker JD, Geissman JW, Bowring SA, Babcock LE, compilers. 2018. Geologic Time Scale v. 5.0: Geological Society of America, https://doi.org/10.1130/2018.CTS005R3C.

Wiens JJ. 2011. Re-evolution of lost mandibular teeth in frogs after more than 200 million years, and re-evaluating Dollo's law. *Evolution* **65**:1283–1296.

Wiens JJ, Kuczynski CA, Smith SA, Mulcahy DG, Sites Jr. JW, Townsend TM, Reeder TW. 2008. Branch length, support, and congruence: testing the phylogenomic approach with 20 nuclear loci in snakes. *Syst Biol.* **57:**420–431.

Wiens JJ, Pyron RA, Moen DC. 2011. Phylogenetic origins of local-scale diversity patterns and the causes of Amazonian megadiversity. *Ecol Lett.* **14**:643–652.

Yuan W, Keqin G, Xing X. 2000. Early evolution of discoglossid frogs: new evidence from the Mesozoic of China. *Naturwissenschaften* **87**:417–420

Zerbino DR, Birney E. 2008. Velvet: algorithms for de novo short read assembly using de Bruijn graphs. *Genome Res.* **18:**821–829.

Zhou X, Shen X-X, Hittinger CT, Rokas A. 2018. Evaluating fast maximum likelihood-based phylogenetic programs using empirical phylogenomic data sets. *Mol Biol Evol.* **35:**486–503.

Figure S1. Swarm plots of alignment characteristics for different marker classes. Comparisons are shown for alignment length, percent informative sites, and percent missing data. Data are also summarized in Table 3.

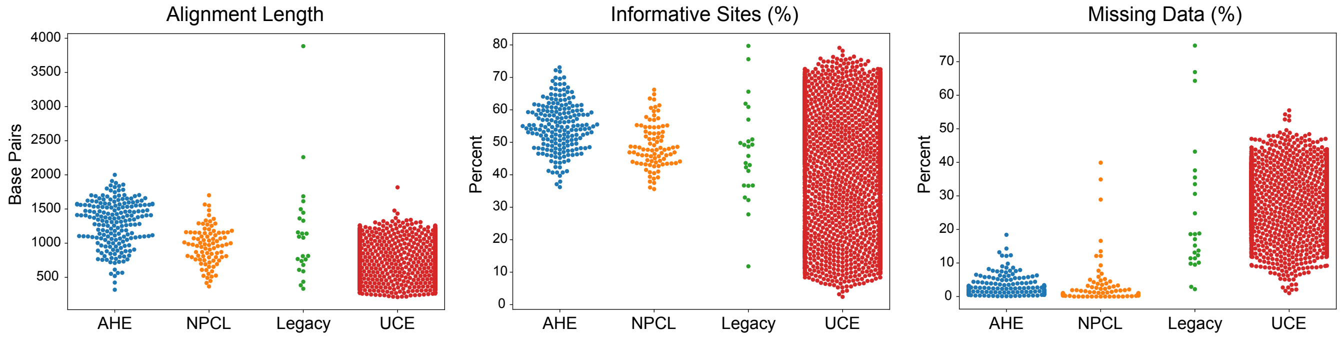

Figure S2. Phylogenetic estimate of anurans based on the species-tree analysis of 3,784 UCE loci using ASTRAL-III. The analysis is based on gene trees generated from untrimmed MAFFT alignments. Scale bar represents coalescent units. Asterisks indicate families that are inferred to be polyphyletic in this tree. Local posterior probabilities for each node are given in Supplementary File S5, Fig. S2.

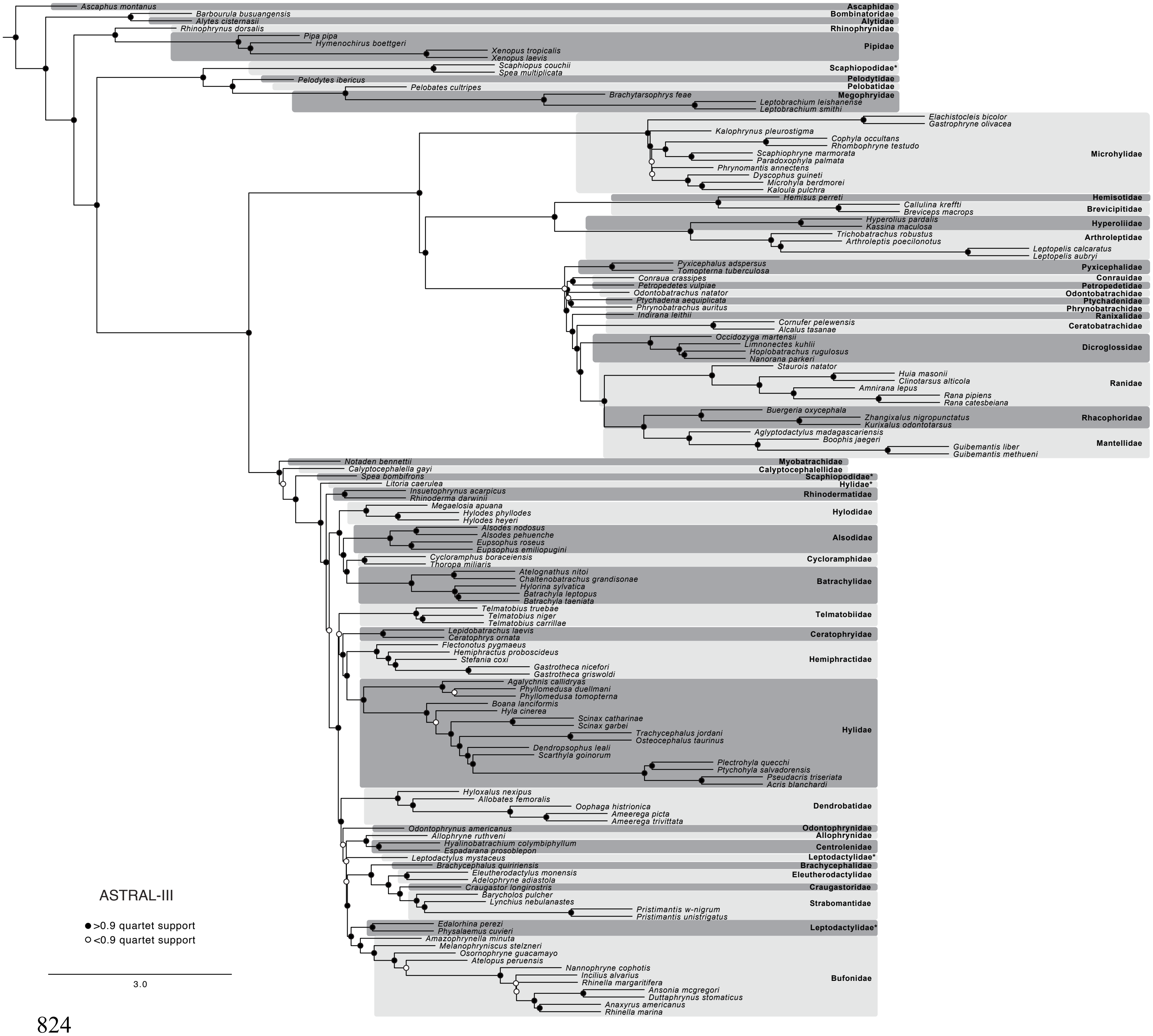

Figure S3. Maximum likelihood tree from the unpartitioned gigamatrix analysis. Scale bar represents substitutions per site. The phylogenetic tree is shown across four panels (A–D), with letters on branches representing connection points across panels. Bootstrap values for each node are given in Supplementary File S5, Fig. S4.

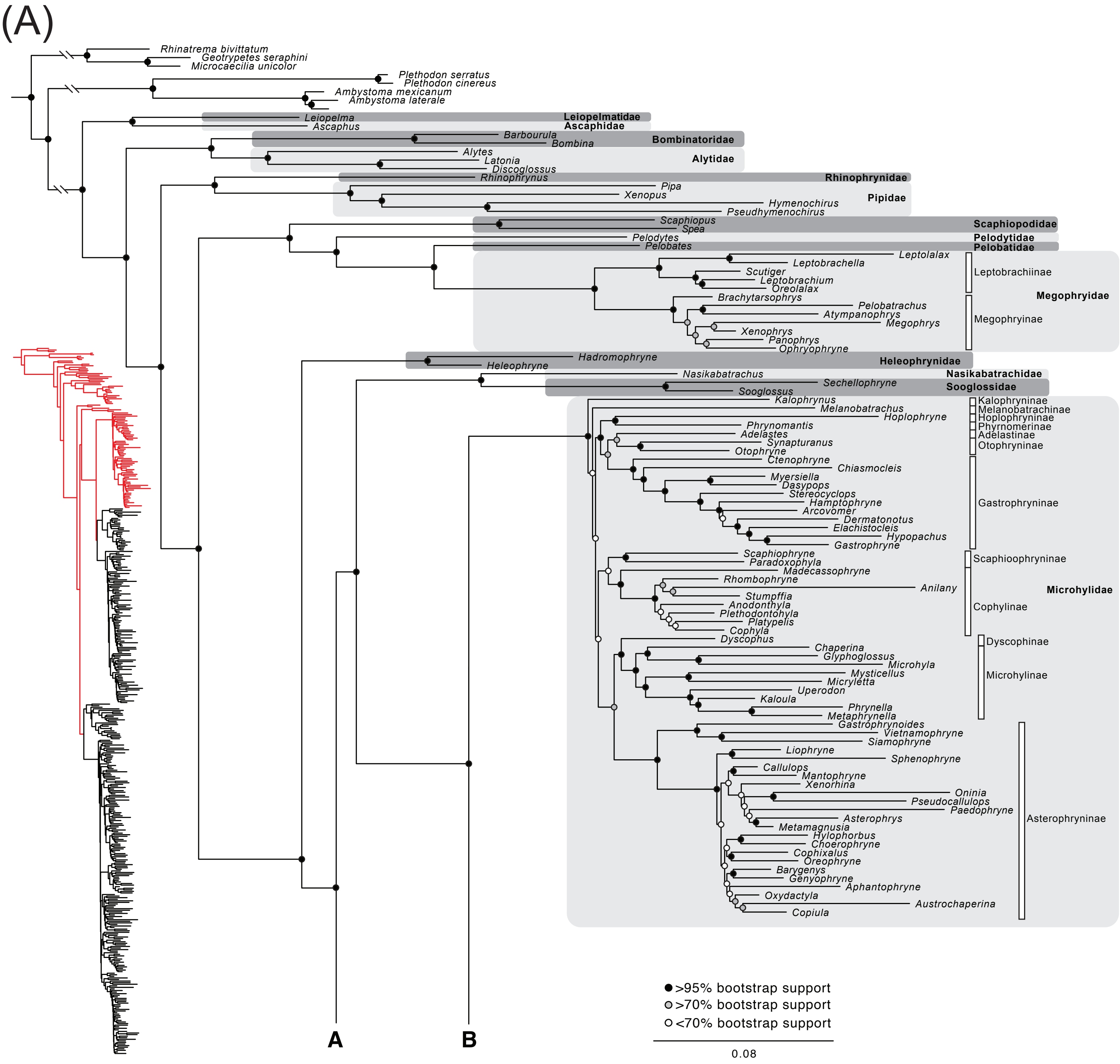

Figure S3. (continued)

(B)

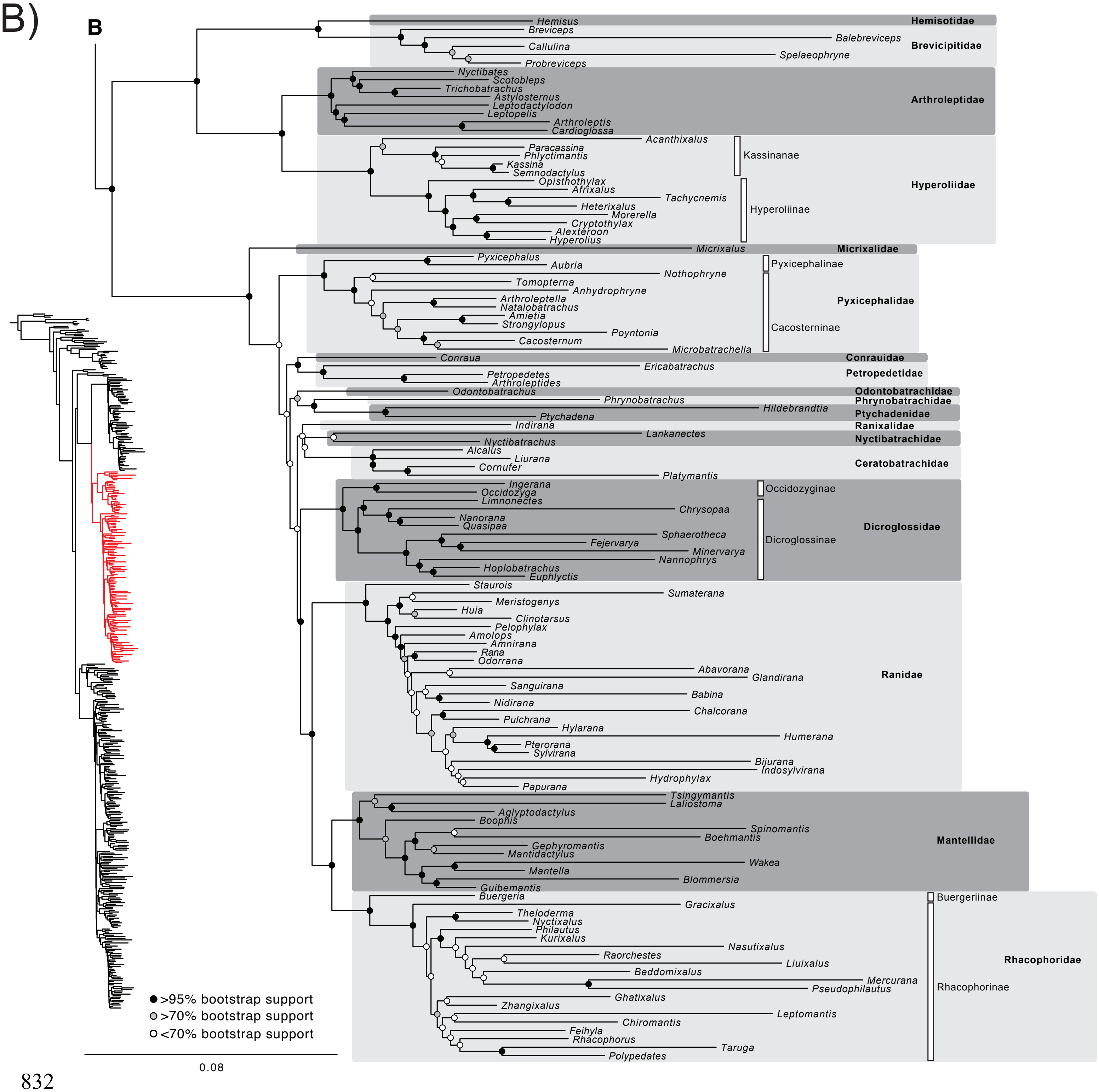

Figure S3. (continued)

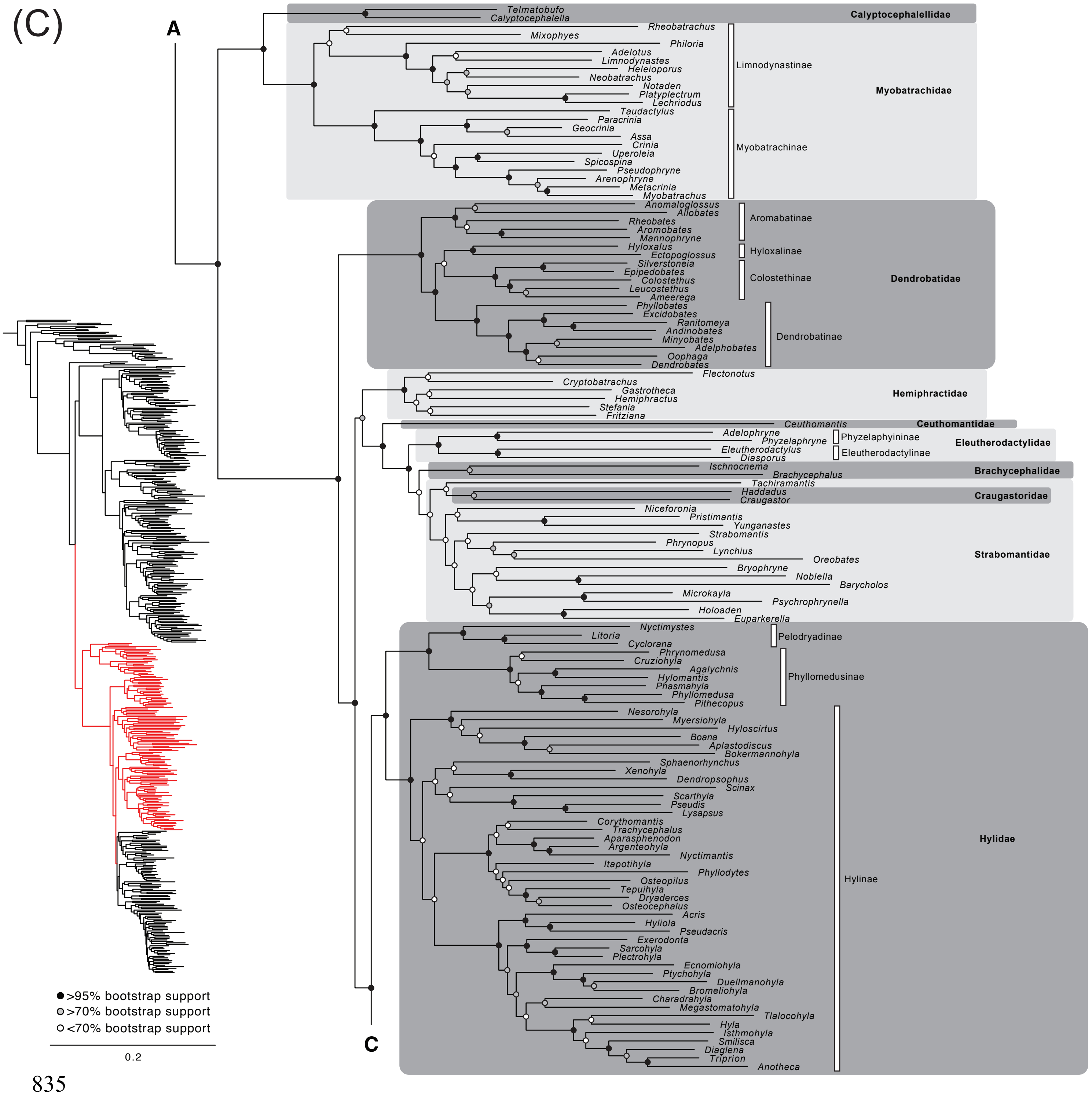

Figure S3. (continued)

(D)

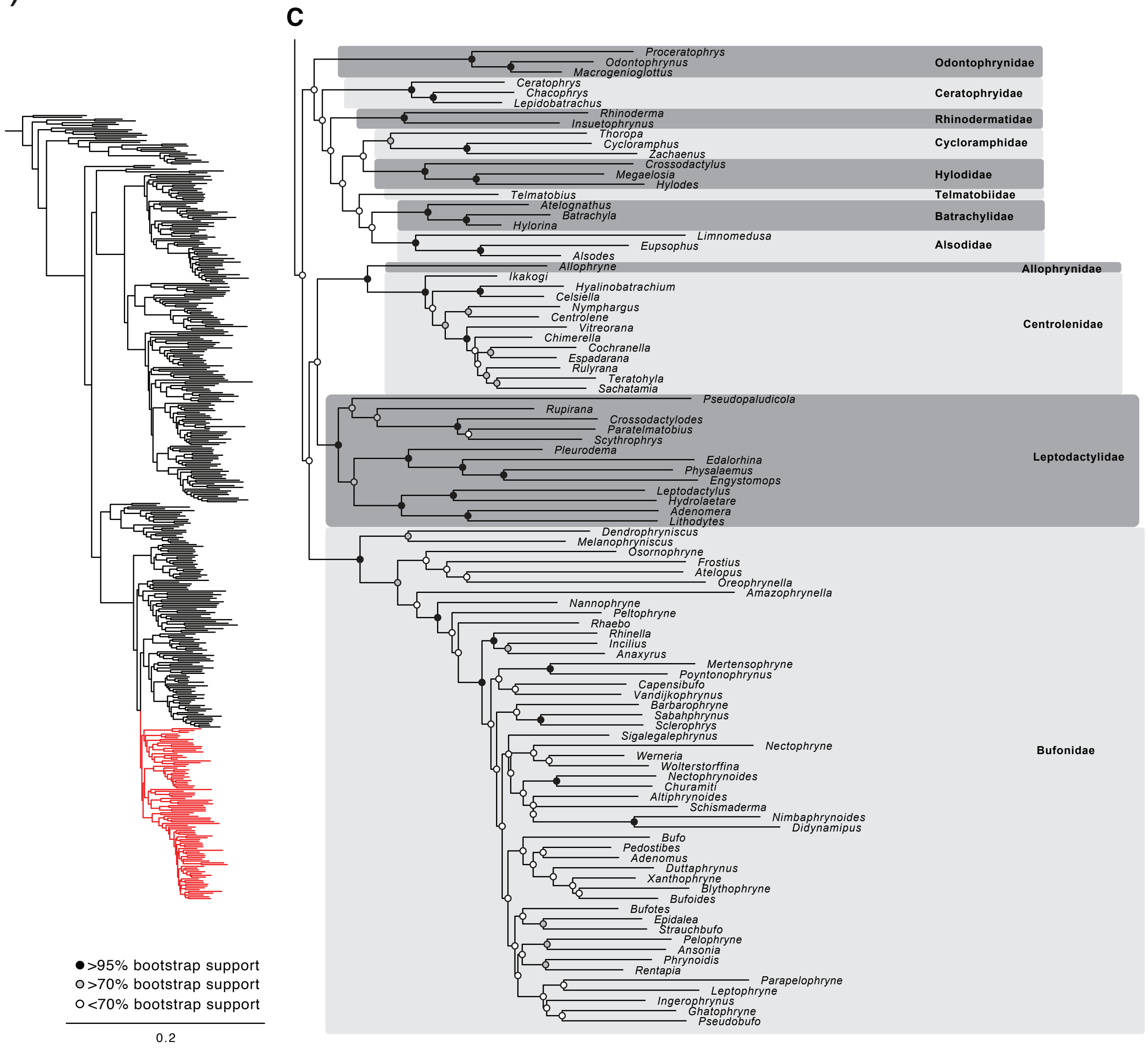

Figure S4. Estimated tree from maximum likelihood analysis of the supermatrix. The supermatrix consists of the 307 AHE, NPCL, and legacy markers (no UCEs included). Scale bar represents substitutions per site. The phylogenetic tree is shown across four panels (A–D), with letters on branches representing connection points across panels. Bootstrap values for each node are given in Supplementary File S5, Fig. S7.

(A)

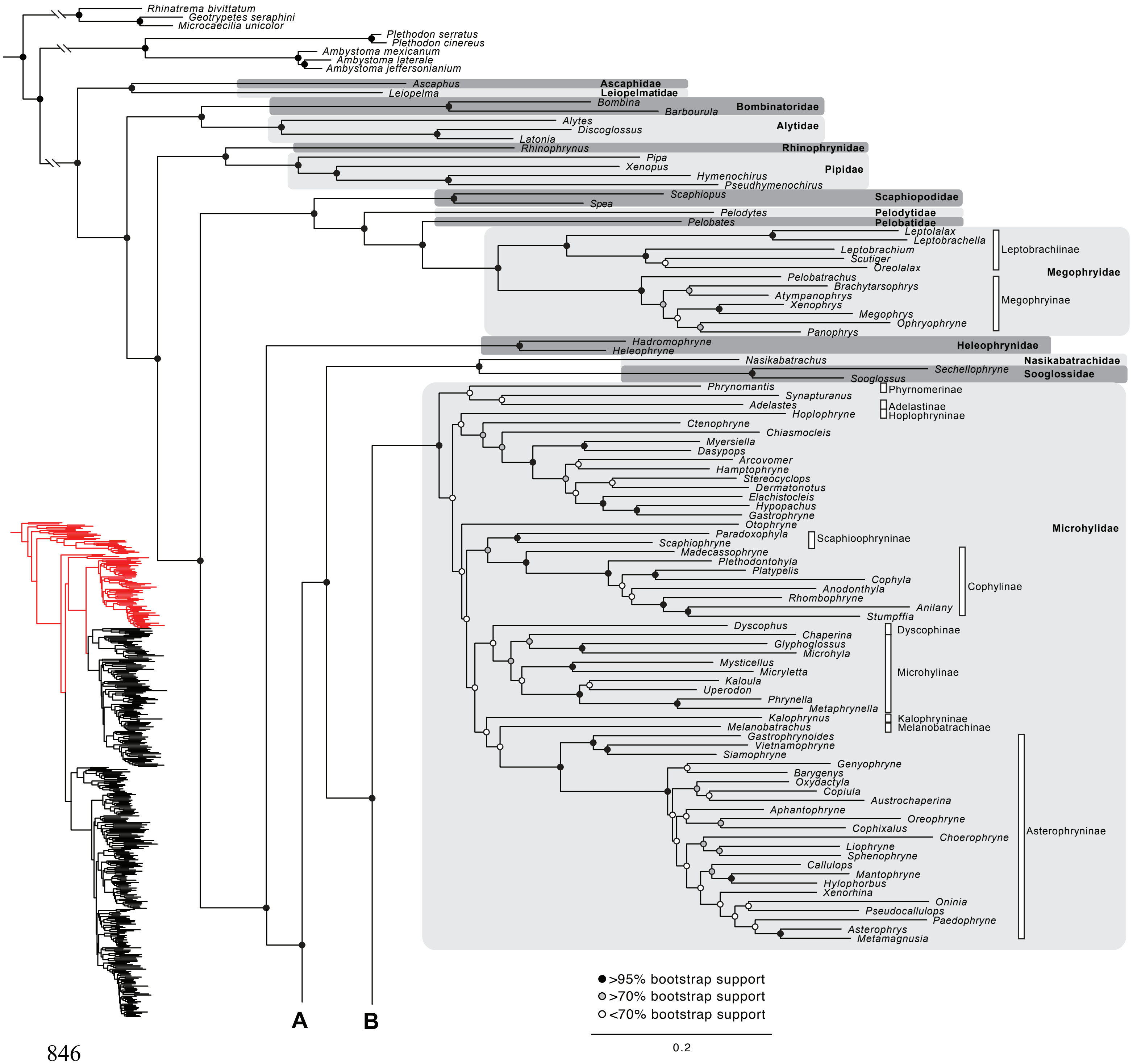

Figure S4. (continued)

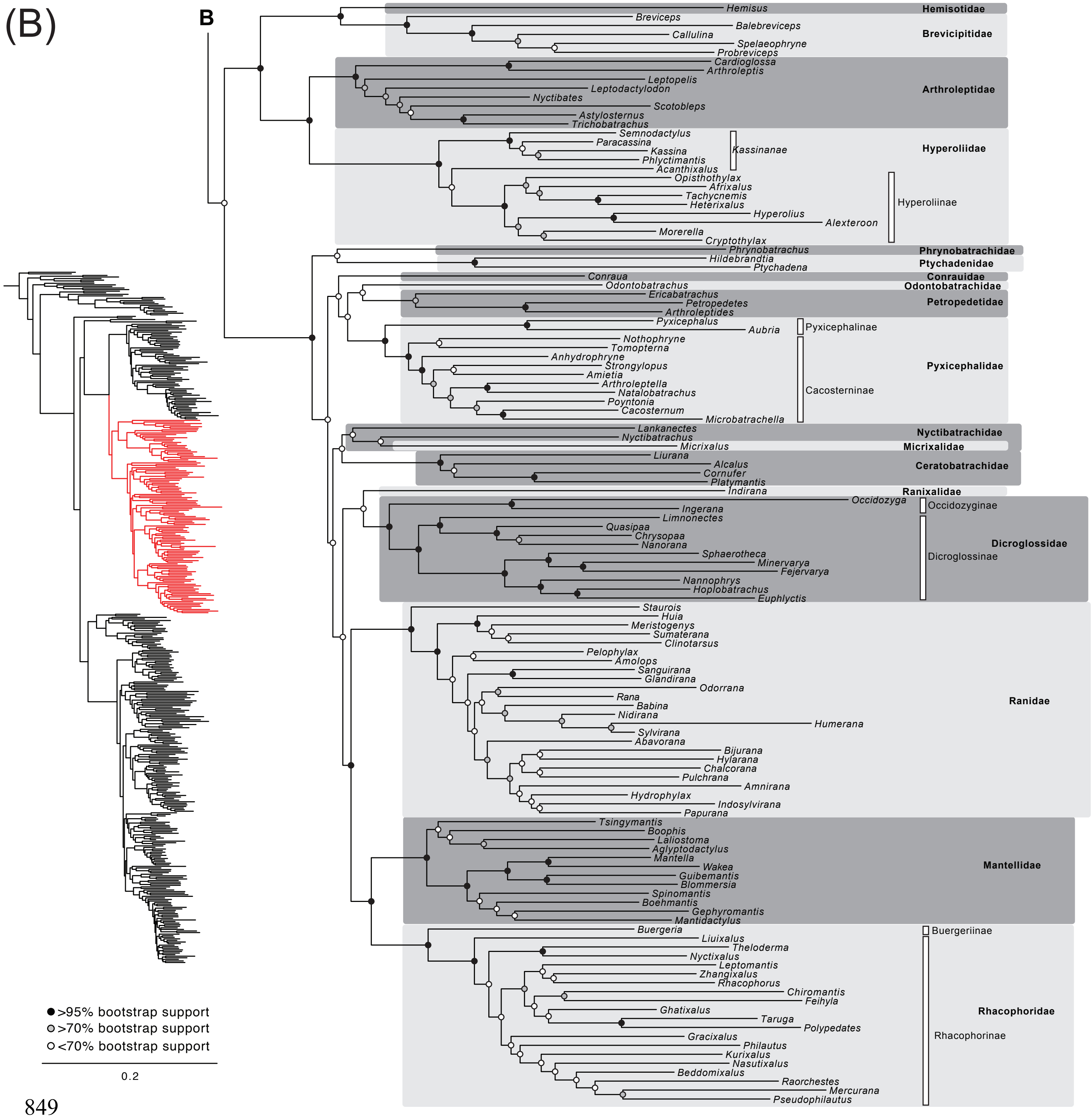

Figure S4. (continued)

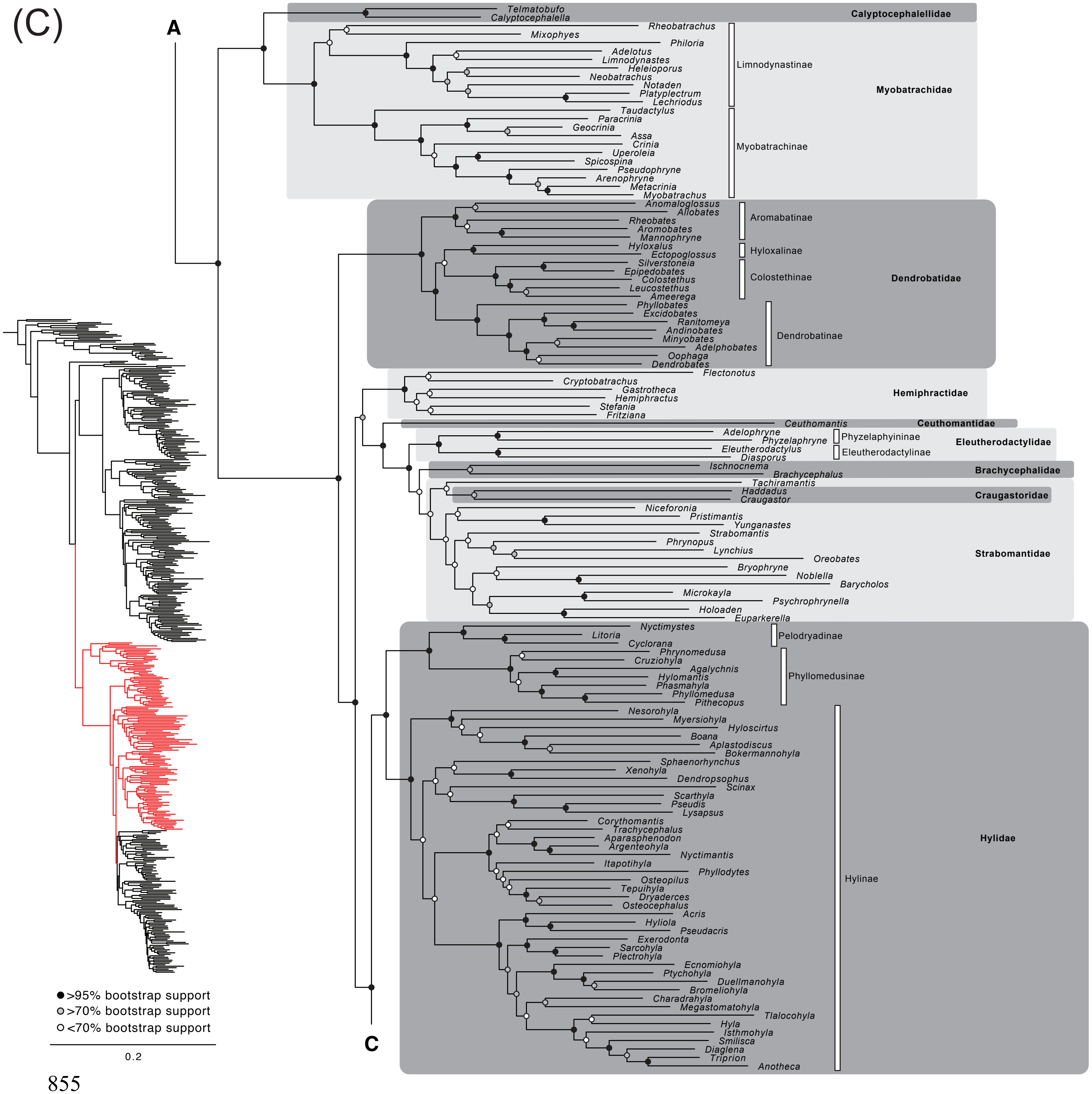

Figure S4. (continued)

(D)

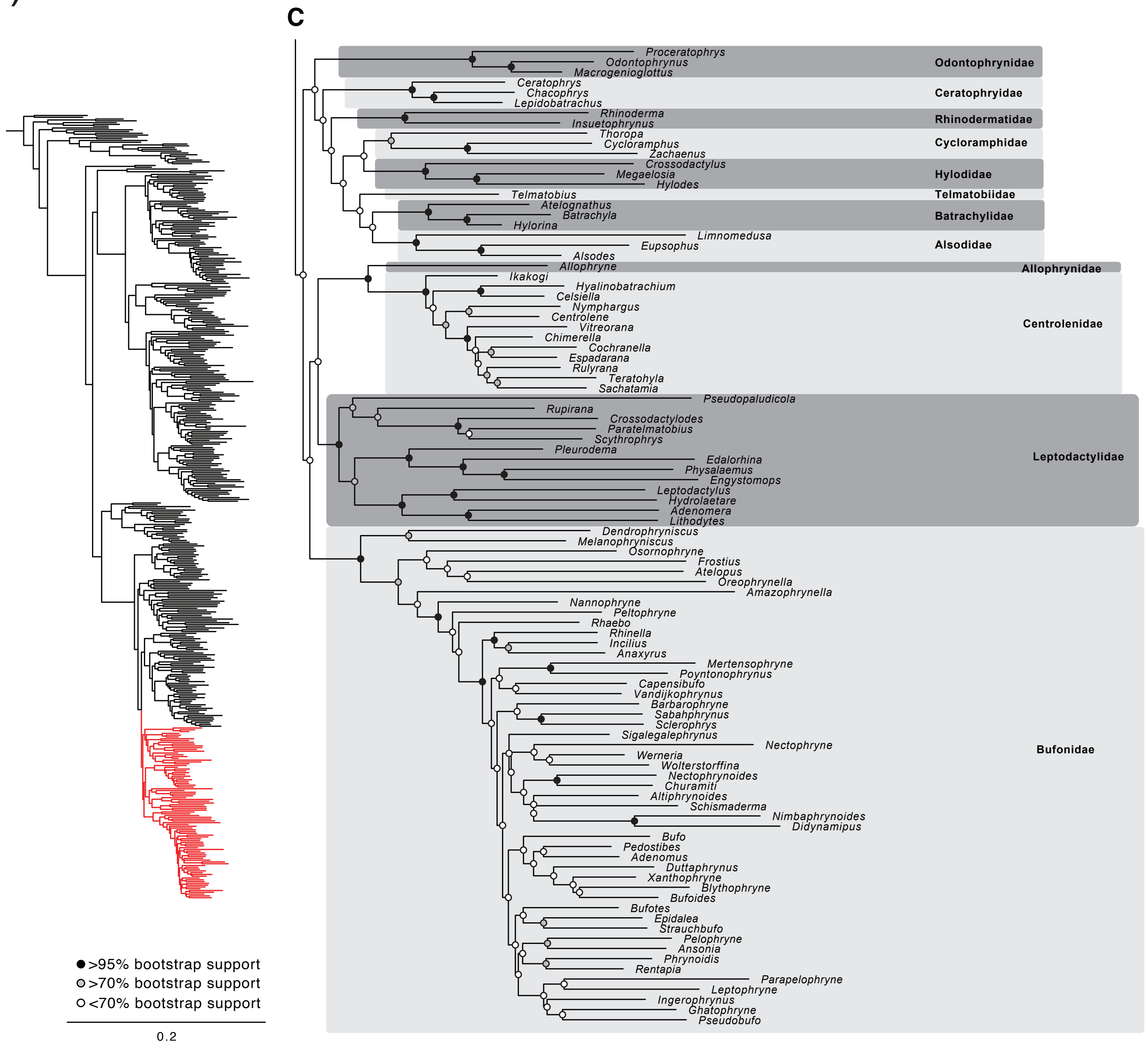

Figure S5. Tree from concatenated maximum likelihood analysis of the gigamatrix, with rogue taxa removed. Scale bar represents substitutions per site. The phylogenetic tree is shown across four panels (A–D), with letters on branches representing connection points across panels. Bootstrap values for each node are given in Supplementary File S5, Fig. S7.

(A)

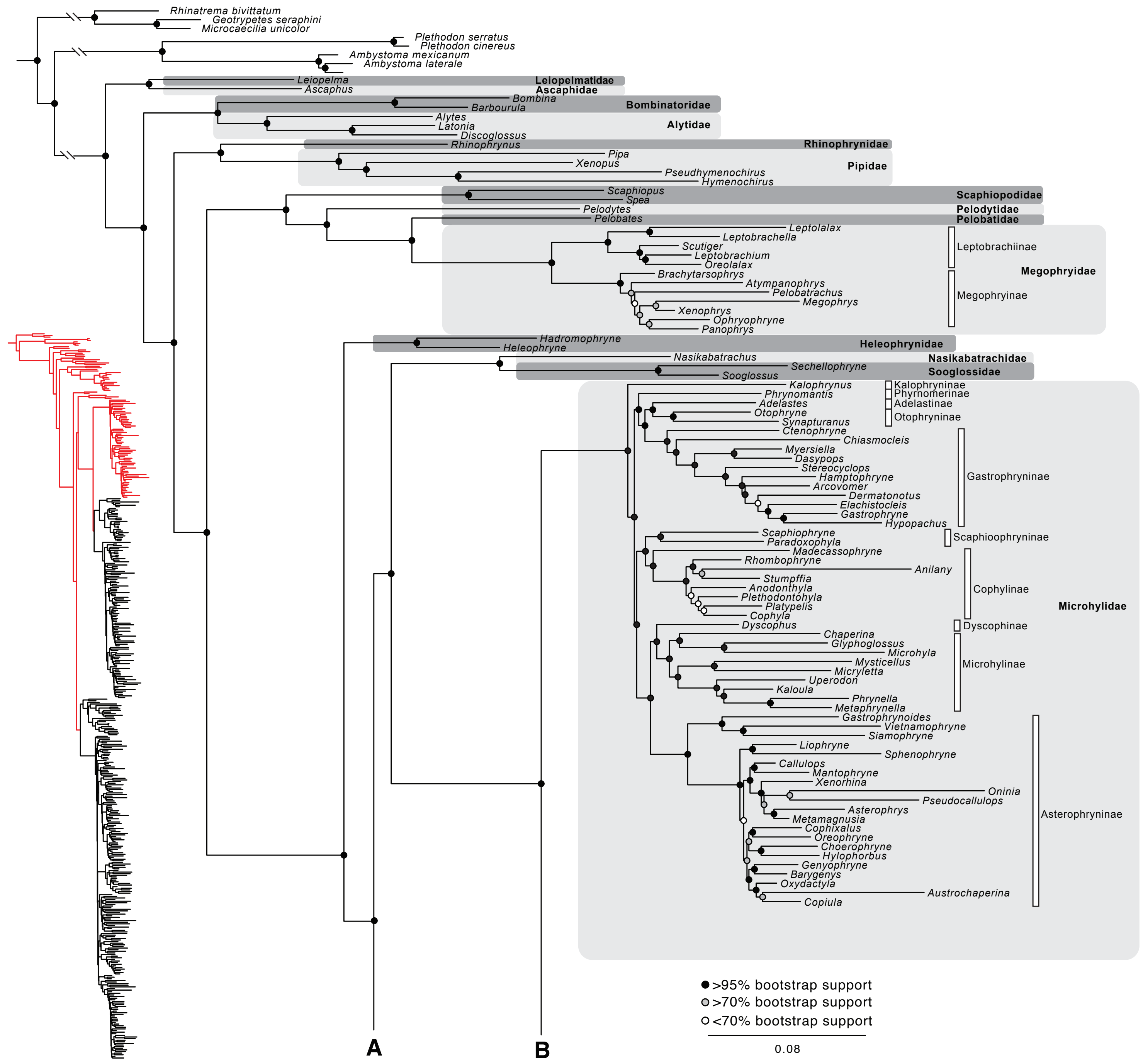

Supplementary Figure S5. (continued)

(B)

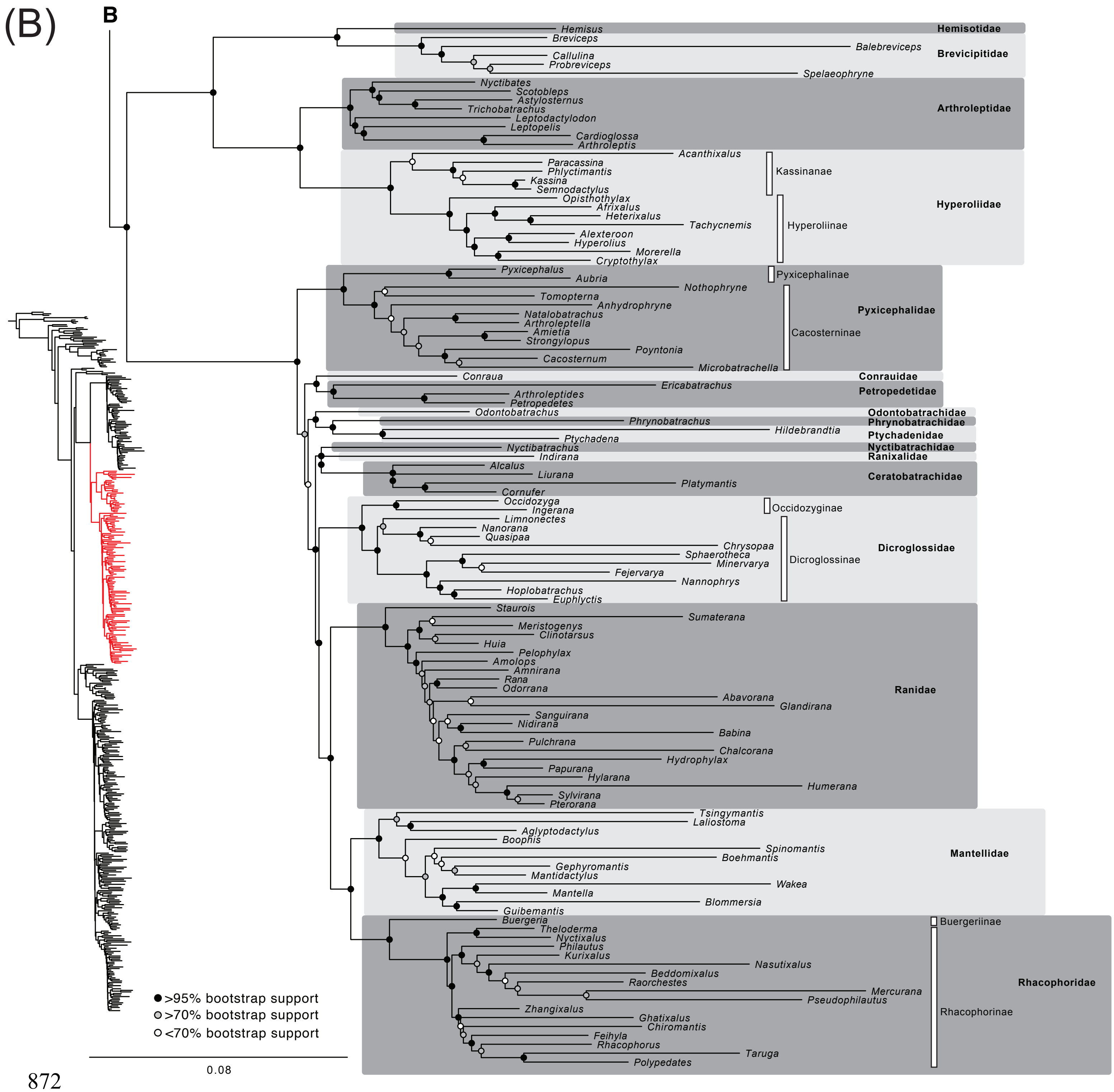

Figure S5. (continued)

(C)

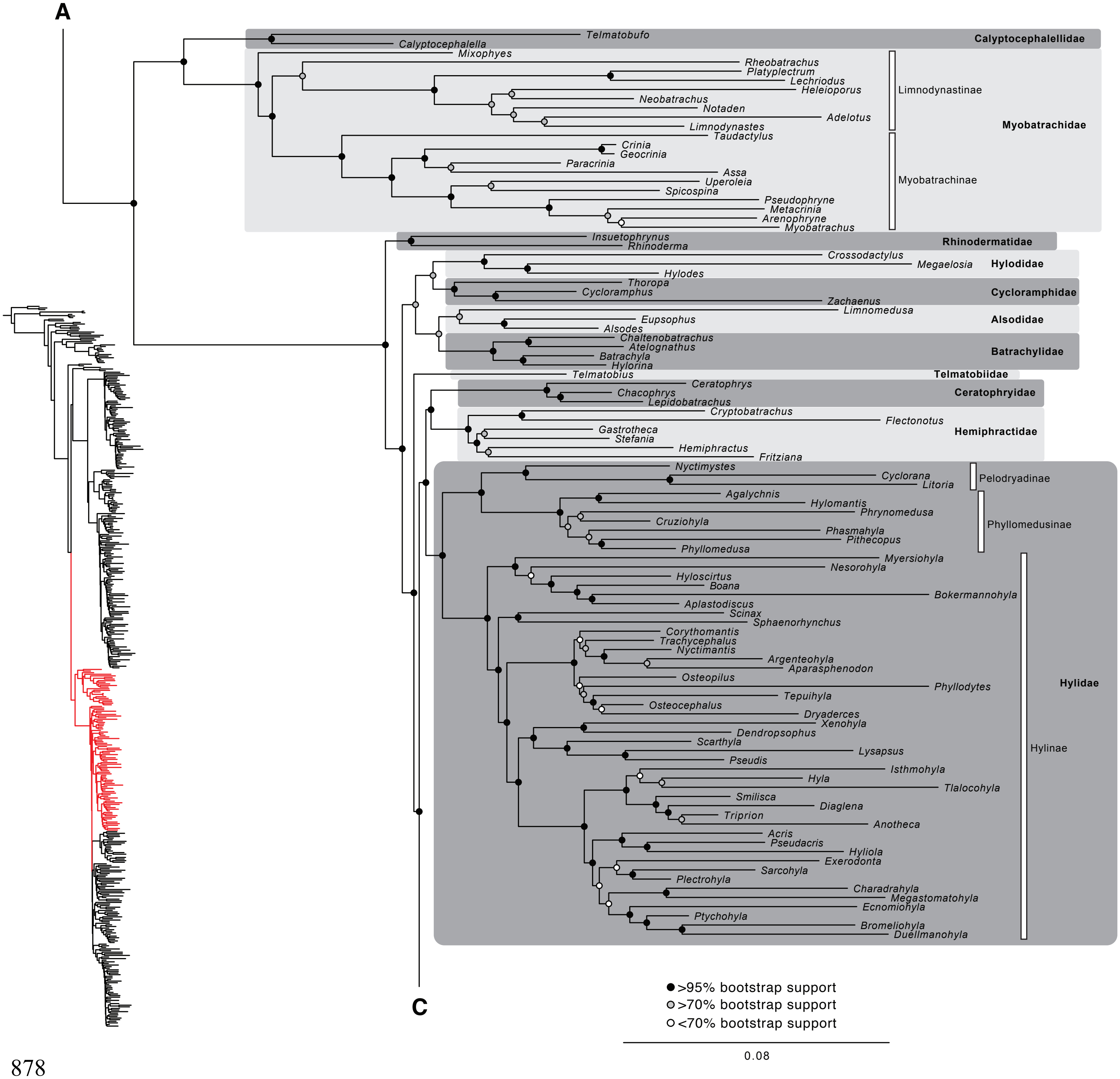

Figure S5. (continued)

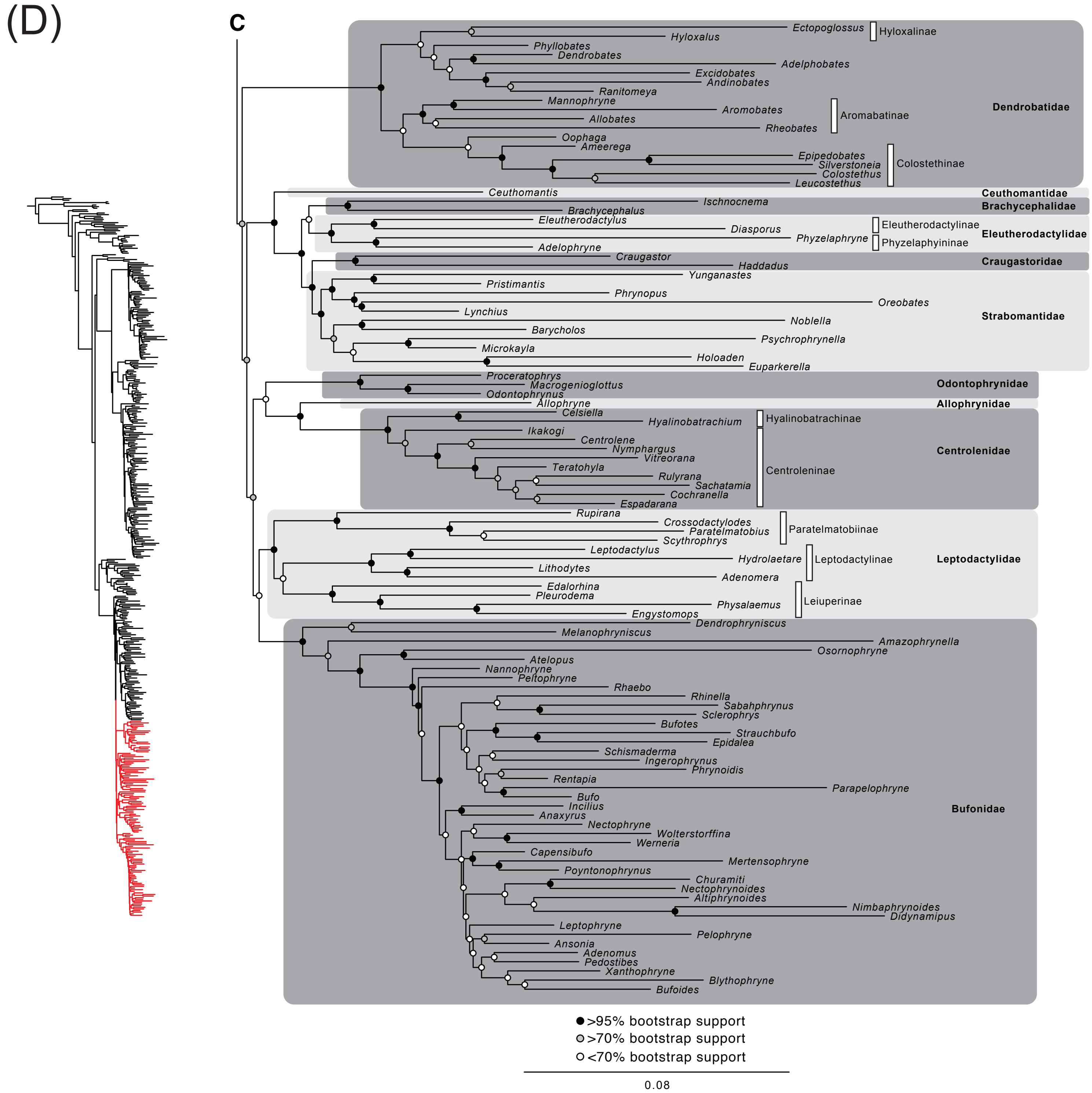

Table S1. Voucher information for tissue samples used this study.

Species	Museum number/ Collector ID	Family	Subfamily
Aglyptodactylus madagascariensis	UF 153651	Mantellidae	Laliostominae
Amnirana lepus	GBC-A 0344	Ranidae	-
Anaxyrus americanus	JJW 729	Bufonidae	-
Arthroleptis poecilonotus	UF 180748	Arthroleptidae	-
Ascaphus montanus	DSM 0840	Ascaphidae	-
Boophis jaegeri	UF 153611	Mantellidae	Boophinae
Brachytarsophrys feae	CAS 224375	Megophryidae	Megophryinae
Breviceps macrops	CAS 193965	Brevicipitidae	-
Buergeria oxycephalus	MVZ 230427	Rhacophoridae	Buergeriinae
Callulina kreffti	CAS 168715	Brevicipitidae	-
Clinotarsus alticola	CAS 232169	Ranidae	-
Conraua crassipes	CAS 258318	Conrauidae	-
Cophyla occultans	UF 153629	Microhylidae	Cophylinae
Cornufer pelewensis	CAS 236907	Ceratobatrachidae	Ceratobatrachinae
Duttaphrynus stomaticus	ID 7602	Bufonidae	-
Dyscophus guineti	MVZ 238743	Microhylidae	Dyscophinae
Gastrotheca griswoldi	MTD-TD 833	Hemiphractidae	-
Guibemantis liber	MVZ 238740	Mantellidae	Mantellinae
Hemisus perreti	UF 180509	Hemisotidae	-
Hoplobatrachus rugulosus	CAS 240996	Dicroglossidae	Dicroglossinae
Hymenochirus boettgeri	CAS 253587	Pipidae	-
Hyperolius pardalis	UF 180880	Hyperoliidae	-
Indirana leithii	ID 7600	Ranixalidae	-
Kalophrynus pleurostigma	CAS 245749	Microhylidae	Kalophryninae
Kaloula pulchra	CAS 210158	Microhylidae	Microhylinae
Kassina maculosa	UF 180614	Hyperoliidae	-
Leptobrachium smithi	CAS 247976	Megophryidae	Leptobrachiinae
Leptopelis aubryi	CAS 258070	Arthroleptidae	-
Leptopelis calcaratus	UF 180849	Arthroleptidae	-
Microhyla berdmorei	CAS 213432	Microhylidae	Microhylinae
Occidozyga martensii	CAS 239421	Dicroglossidae	Occidozyginae
Odontobatrachus natator	CAS 230055	Odontobatrachidae	-
Pelobates cultripes	MVZ 231951	Pelobatidae	-
Pelodytes ibericus	MVZ 186009	Pelodytidae	-
Petropedetes vulpiae	GBC-A 0526	Petropedetidae	-
Phrynobatrachus auritus	UF 180893	Phrynobatrachidae	-

Phrynomantis annectens	CAS 255056	Microhylidae	Phrynomerinae
Phyllomedusa duellmani	KU 212206	Hylidae	Phyllomedusinae
Pipa pipa	MVZ 247509	Pipidae	-
Ptychadena aequiplicata	UF 180901	Ptychadenidae	-
Rana pipiens	JJW 748	Ranidae	-
Rhinophrynus dorsalis	MVZ 137712	Rhinophrynidae	-
Rhombophryne testudo	UF 153589	Microhylidae	Cophylinae
Scaphiophryne marmorata	MCZ A-136273	Microhylidae	Scaphiophryninae
Scaphiopus couchii	DMP 1742	Scaphiopodidae	-
Tomopterna tuberculosa	JET 0033	Pyxicephalidae	Cacosterninae
Trachycephalus jordani	KU 217771	Hylidae	Hylinae
Trichobatrachus robustus	UF 180821	Arthroleptidae	-
Zhangixalus nigropunctatus	CAS 242203	Rhacophoridae	Rhacophorinae

Abbreviations are as follows: CAS: California Academy of Sciences; DSM: Daniel S. Moen field series; DMP: Daniel M. Portik field series; GBC, Gabon Biodiversity Collection; ID: Indraneil Das field series; JET: James Titus-McQuillan field series; JJW: John J. Wiens field series; KU: Biodiversity Institute and Natural History Museum, University of Kansas; MCZ: Museum of Comparative Zoology, Harvard University; MVZ: Museum of Vertebrate Zoology, UC Berkeley; UF: Florida Museum of Natural History, University of Florida.

Table S2. List of rogue taxa identified by RogueNaRok.

Genus	Family
Anomaloglossus	Dendrobatidae
Aphantophryne	Microhylidae
Barbarophryne	Bufonidae
Bijurana	Randae
Bryophryne	Craugastoridae
Chimerella	Centrolenidae
Duttaphrynus	Bufonidae
Frostius	Bufonidae
Ghatophryne	Bufonidae
Gracixalus	Rhacophoridae
Hoplophryne	Microhylidae
Indosylvirana	Ranidae
Itapotihyla	Hylidae
Lankanectes	Nyctibatrachidae
Leptomantis	Rhacophoridae
Liuixalus	Rhacophoridae
Melanobatrachus	Microhylidae
Micrixalus	Micrixalidae
Minyobates	Dendrobatidae
Niceforonia	Craugastoridae
Oreophrynella	Bufonidae
Paedophryne	Microhylidae
Philoria	Myobatrachidae
Pseudobufo	Bufonidae
Pseudopaludicola	Leptodactylidae
Sigalegalephrynus	Bufonidae
Strabomantis	Strabomantidae
Tachiramantis	Strabomantidae
Vandijkophrynus	Bufonidae

Table S3. Comparison of congruence and support among higher-level nodes in the gigamatrix, UCE, and supermatrix trees. For each clade in the partitioned gigamatrix ML tree, we give the bootstrap support values (Giga-BS). We also give the bootstrap values for the higher-level clades in the UCE-only concatenated ML tree (UCE-BS) and in the supermatrix tree (Super-BS). For the UCE tree, if the relevant taxa in a clade were not present in the UCE tree, this is noted under "UCE clade." We also note here if a conflicting clade was supported instead, and the bootstrap value for that clade is given under UCE-BS. Similarly, under "Supermatrix clade" we report if a different clade was supported, and the bootstrap value for that clade is given under Super-BS (note that the listing of supermatrix clades that are in conflict with the gigamatrix tree do not align perfectly with clades in the gigamatrix tree). If nothing is listed under UCE clade and/or under Supermatrix clade, then that clade in the gigamatrix tree is supported in one or more of those trees. Bootstrap values of clades that are incongruent with the gigamatrix tree are also indicated in red.

**Higher-level clade in gigamatrix tree**	**Giga-BS**	**UCE-BS**	**UCE clade**	**Super-BS**	**Supermatrix clade**
Frog monophyly	100	100		100	
Leiopelmatoidea	100		no *Leiopelma*	100	
Frogs above Leiopelmatoidea	100	100		94	
Discoglossoidea	100	100		100	
Frogs above Discoglossoidea	100	100		92	
Pipoidea	100	100		100	
Frogs above Pipoidea	100	100		94	
Pelobatoidea	100	100		100	
Pelodytidae+Pelobatidae+Megophryidae	100	100		95	
Pelobatidae+Megophryidae	100	100		100	
Neobatrachia	100	100		100	
Frogs above Heleophrynidae	100		no Heleophrynidae	93	
Ranoidea+Sooglossidae+Nasikabatrachidae	100		no Sooglossidae, Nasikabatrachidae	69	
Sooglossidae+Nasikabatrachidae	100		no Sooglossidae, Nasikabatrachidae	100	
Ranoidea	100	100		100	
Afrobatrachia+Natatanura	100	100		37	
Afrobatrachia	100	100		100	
Hemisotidae+Breviceptidae	100	100		100	
Arthroleptidae+Hyperoliidae	100	100		100	

Natatanura	100	100		100	
Natatanura above Micrixalidae	33		no Micrixalidae	7	Natatanura above Phrynobatrachidae+Ptychadenidae
Natatanura above Pyxicephalidae	31	100		23	Conrauidae+Odontobatrachidae+Petropedetidae+Pyxicephalidae
Conrauidae+Petropedetidae	97	100		22	Odontobatrachidae+Petropedetidae+Pyxicephalidae
Natatanura above Conrauidae+Petropedetidae	28	89	Conrauidae+Petropedetidae+Odontobatrachidae+Phrynobatrachidae+Ptychadenidae	32	Odontobatrachidae+Petropedetidae
Odontobatrachidae+Phrynobatrachidae+Ptychadenidae	97	100		4	Natatanura above Phrynobatrachidae+Ptychadenidae+Conrauidae+Odontobatrachidae+Petropedetidae+Pyxicephalidae
Phrynobatrachidae+Ptychadenidae	99	100		11	
Natatanura above Odontobat+Phryno+Ptychadenidae	38	100		4	Micrixalidae+Nyctibatrachidae+Ceratobatrachidae
Ranixalidae+Nyctibatrachidae+Ceratobatrachidae	36	100	no Nyctibatrachidae	17	Micrixalidae+Nyctibatrachidae
Nyctibatrachidae+Ranixalidae	18		no Nyctibatrachidae	13	Dicroglossidae+Ranixalidae+Ranidae+Mantellidae+Rhacophoridae
Natatanura above Ranixalidae+Nyctibatrachidae+Ceratobatrachidae	97	100		14	Dicroglossidae+Ranixalidae
Ranidae+Mantellidae+Rhacophoridae	98	100		61	
Mantellidae+Rhacophoridae	100	100		90	
Hyloidea + Myobatrachidae+Calyptocephalellidae	100	100		100	
Myobatrachidae+Calyptocephalellidae	100	100	Calyptocephalellidae+Hyloidea	100	
Hyloidea	100	100		100	
Hyloidea above Rhinodermatidae	99	100		97	Hyloidea above Dendrobatidae

Neoaustrarana	87	100		87	Hemiphractidae+Terrarana
Cycloramphidae+Hylodidae	100	100		43	
Alsodidae+Batrachylidae	87	100	Batrachylidae+Cycloramphidae+Hylodidae	61	
Hyloidea above Neoaustrarana	100	100		99	Hyloidea above Dendrobatidae, Hemiphractidae+Terrarana
Hyloidea above Telmatobiidae	87	100		40	Hyloidea above Hylidae
Amazorana	100	100		76	Odontophrynidae+ Ceratophryidae+ Rhinodermatidae+ Cycloramphidiae+Hylodidae+Telmatobiidae+Alsodidae+Batrachytlidae
Ceratophryidae+Hemiphractidae	83	78		28	Ceratophryidae+Rhinodermatidae+ Cycloramphidiae+Hylodidae+Telmatobiidae+Alsodidae+Batrachytlidae
Commutibarana	87	100		30	Rhinodermatidae+ Cycloramphidiae+Hylodidae+Telmatobiidae+Alsodidae+Batrachytlidae
Commutibarana above Dendrobatidae	87	99		25	Cycloramphidiae+Hylodidae+Telmatobiidae+Alsodidae+Batrachytlidae
Terrarana	100	100		100	
Terrarana above Ceuthomantidae	100		no Ceuthomantidae	97	
Eleutherodactylidae+Strabomantidae+Craugastoridae	74	100		61	Brachycephalidae+Strabomantidae+ Craugastoridae
Strabomantidae+Craugastoridae	89	100		38	
Commutibarana above Terrarana	87	100		41	Telmatobiidae+Alsodidae+Batrachylidae
Leptodactylidae+Allophrynidae+Centrolenide	66	100		36	
Allophrynidae+Centrolenide	100	100		100	
Odontophrynidae+Bufonidae	87	100		23	Bufonidae+Leptodactylidae+Centrolenidae+Allophrynidae
Mean bootstrap	88.52	99.26		67.06	

Table S4. Comparison of estimated clade ages in this study (gigamatrix tree, all taxa included) to a selection other recent trees. Ages are approximate for most clades, since many studies did not provide labeled, time-calibrated trees. The newick tree is available as Supplementary File S18.

Clade	This study	Feng et al. (2017)	Hime et al. (2021)	Jetz and Pyron (2018)
Crown Anura	178	210	210	222
Crown Leiopelmatoidea	107	195	200	160
Crown Alytoidea	125	140	140	168
Crown Pipoidea	148	160	160	168
Crown Pelobatoidea	135	125	120	151
Crown Neobatrachia	136	140	140	178
Crown Hyloidea	82	70	70	157
Crown Ranoidea	103	100	105	143

Table S5. Comparison of estimated clade ages in this study (gigamatrix tree, rogue taxa excluded) to a selection other recent trees. Ages are approximate for most clades, since many studies did not provide labeled, time-calibrated trees. The newick tree is available as Supplementary File S19.

Clade	This study	Feng et al. (2017)	Hime et al. (2021)	Jetz and Pyron (2018)
Crown Anura	177	210	210	222
Crown Leiopelmatoidea	100	195	200	160
Crown Alytoidea	125	140	140	168
Crown Pipoidea	148	160	160	168
Crown Pelobatoidea	135	125	120	151
Crown Neobatrachia	138	140	140	178
Crown Hyloidea	87	70	70	157
Crown Ranoidea	106	100	105	143
