# Supplementary material for: Redefining Possible: Combining Phylogenomic and Supersparse Data in Frogs
Source: Mol Biol Evol. 2023 May 4;40(5):msad109. doi: 10.1093/molbev/msad109 (PMC10202597; doi:10.1093/molbev/msad109)
Supplement: msad109_Supplementary_Data [file msad109_supplementary_data.zip › Supplementary File S5.pdf]

Supplementary File 5, Figure S1. Concatenated maximum likelihood analysis of the UCE dataset consisting of 3,784 markers. Scale bar represents substitutions per site. Bootstrap support shown on nodes.

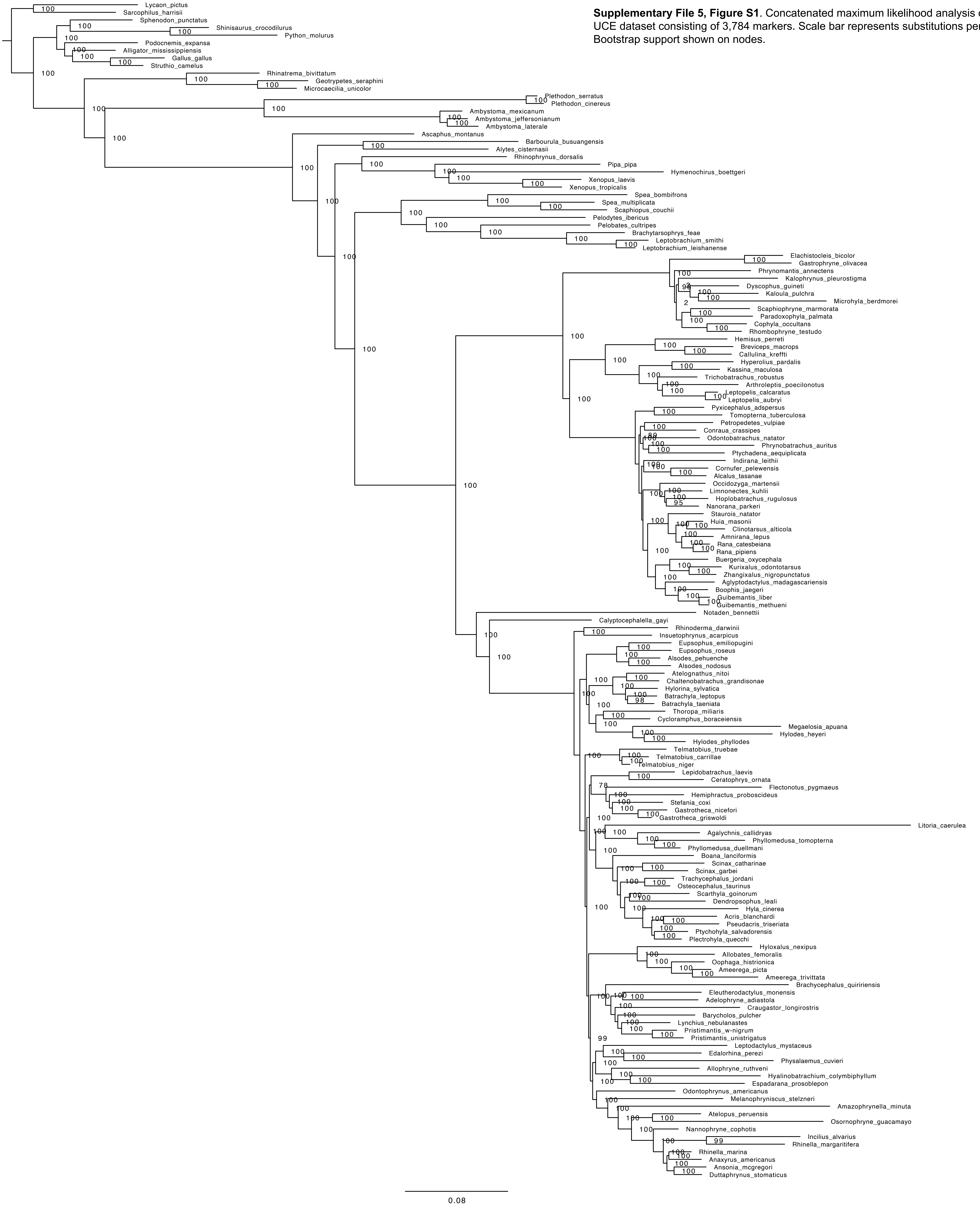

Supplementary File 5, Figure S2. ASTRAL-III species tree analysis of the UCE dataset consisting of 3,784 markers. Scale bar represents coalescent units. Local posterior probabilities are shown on nodes.

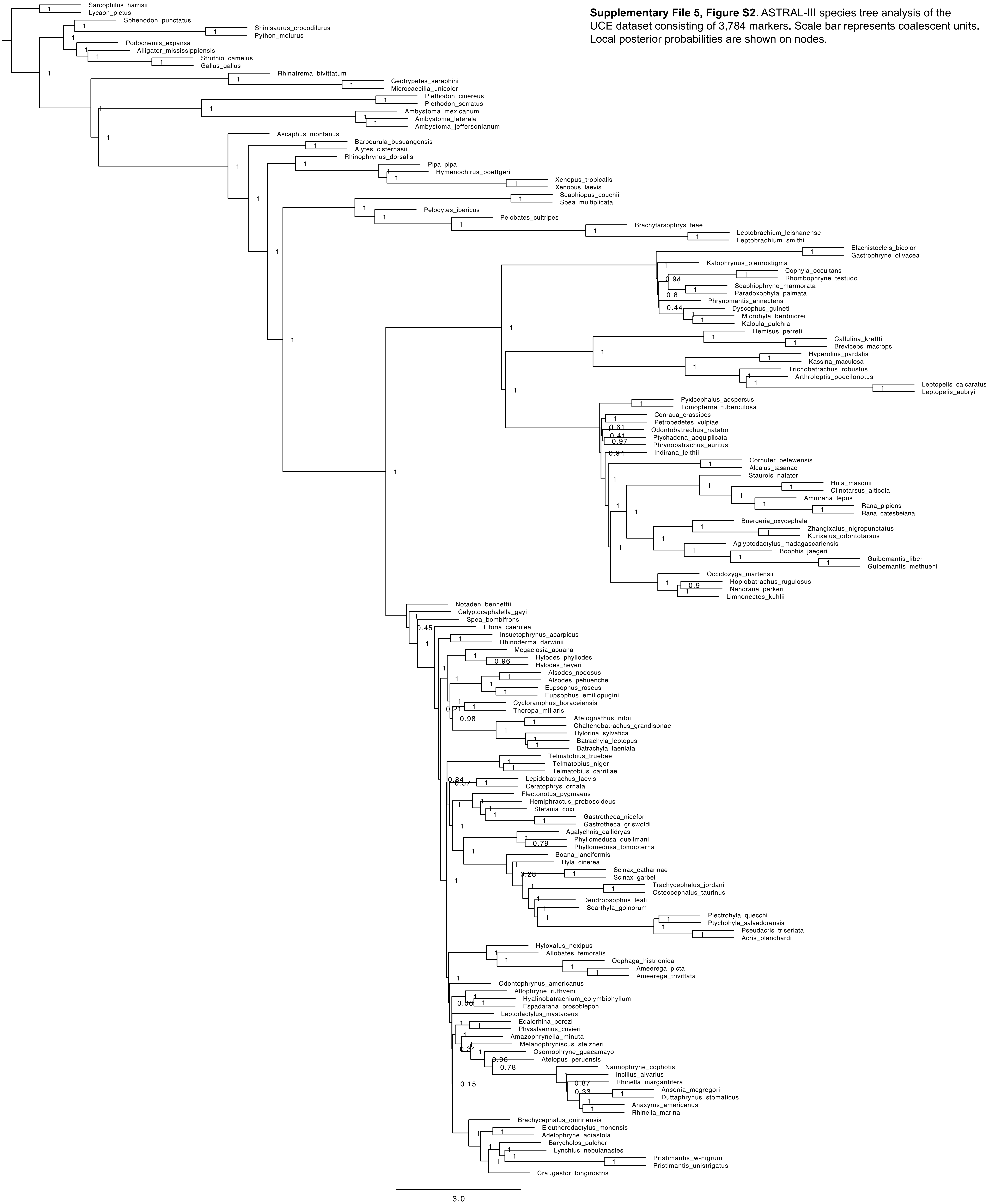

Supplementary File 5, Figure S3. ASTRAL-III species tree analysis of the UCE dataset consisting of 3,784 markers. Scale bar represents coalescent units. Quartet support is shown on nodes.

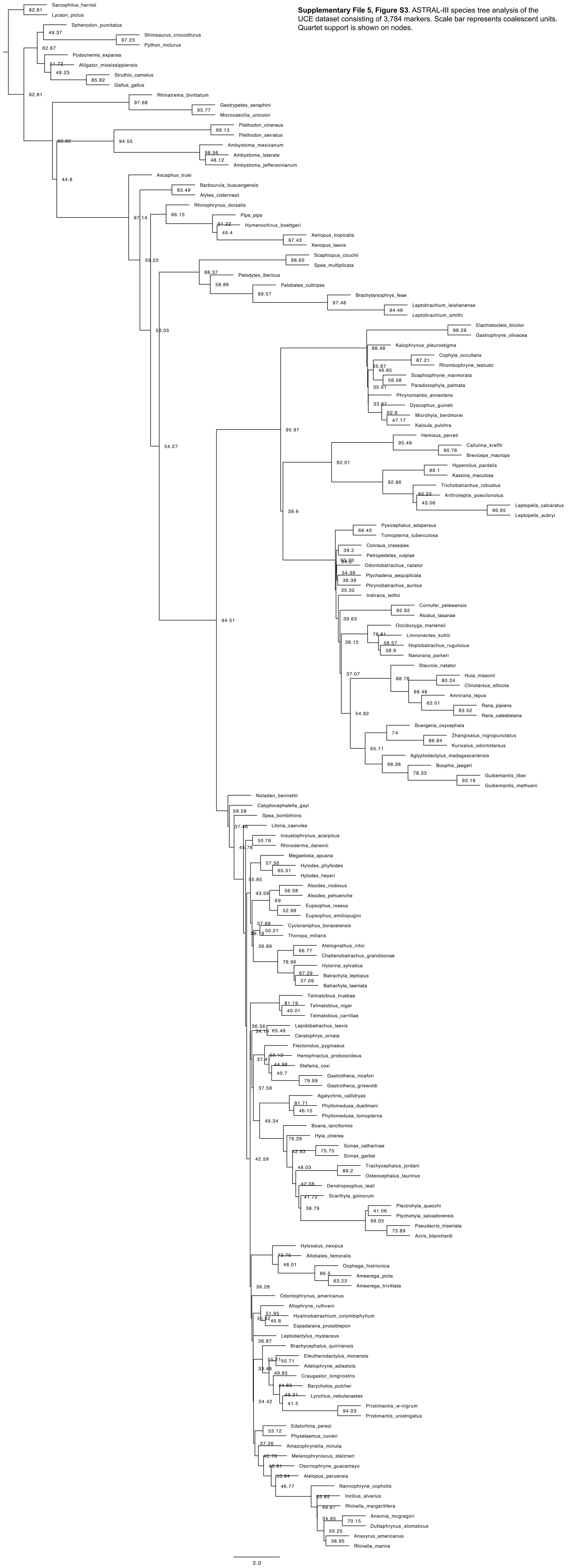

Supplementary File 5, Figure S4. Concatenated maximum likelihood analysis of the gigamatrix dataset consisting of 4,091 markers (including UCEs, AHE, NPCL, and legacy markers), using a single partition for all data. Scale bar represents substitutions per site. Bootstrap support shown on nodes.

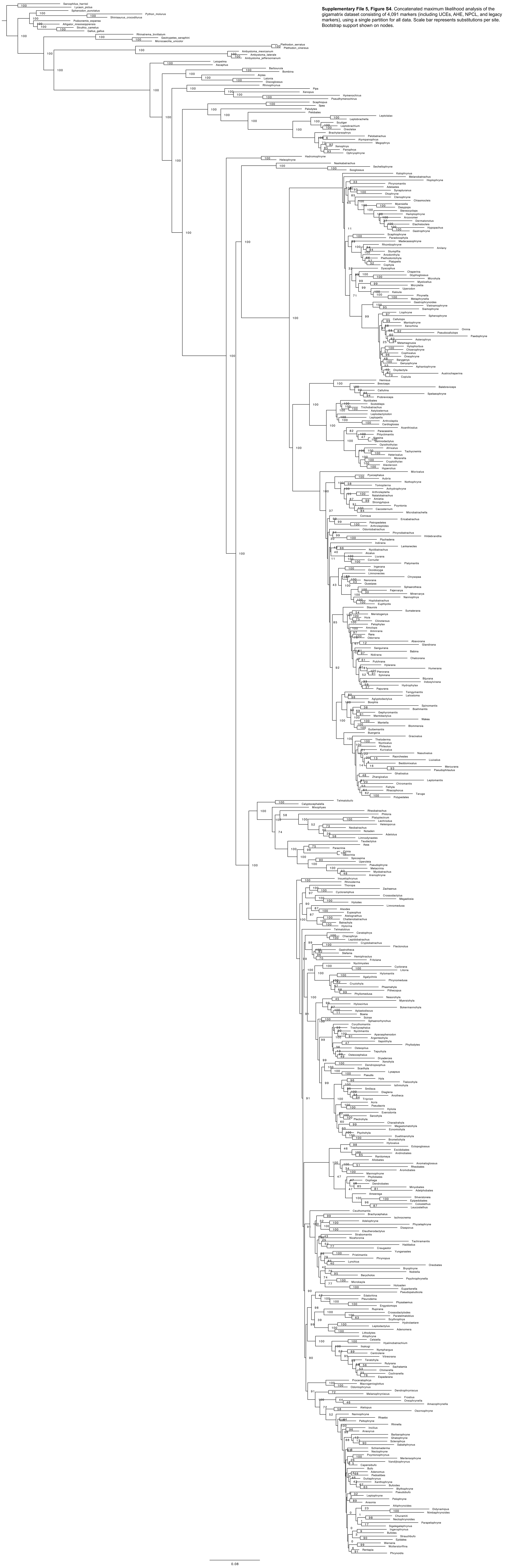

Supplementary File 5, Figure S5. Partitioned concatenated maximum likelihood analysis of the gigamatrix dataset consisting of 4,091 markers (including UCEs, AHE, NPCL, and legacy markers). Here, node colors represent the concordance of nodes shared among the 10 optimized trees, which are annotated on the optimal tree. Scale bar represents substitutions per site.

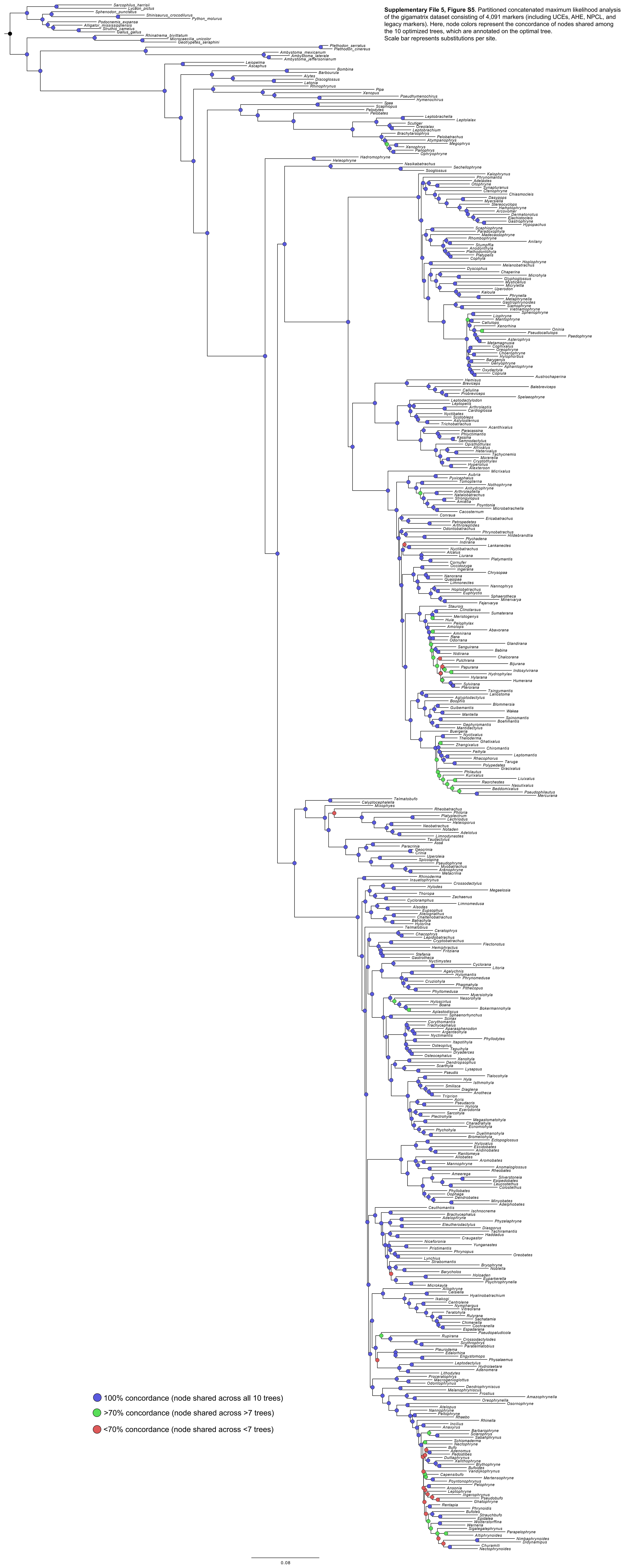

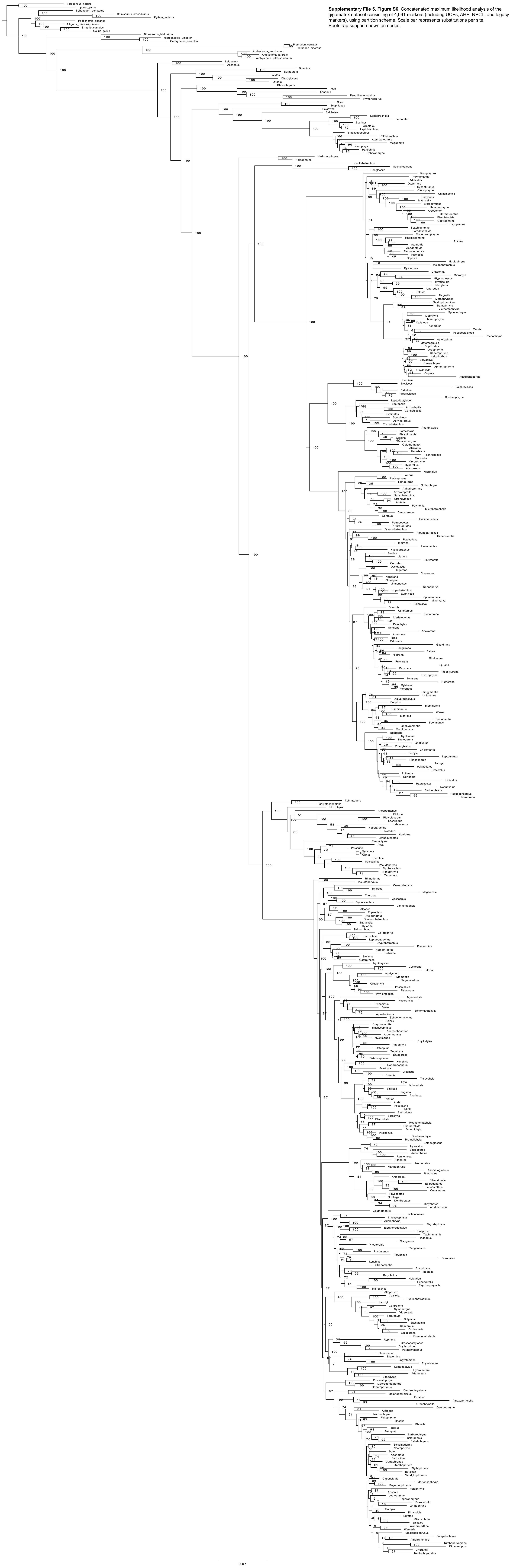

Supplementary File 5, Figure S6. Concatenated maximum likelihood analysis of the gigamatrix dataset consisting of 4,091 markers (including UCEs, AHE, NPCL, and legacy markers), using partition scheme. Scale bar represents substitutions per site. Bootstrap support shown on nodes.

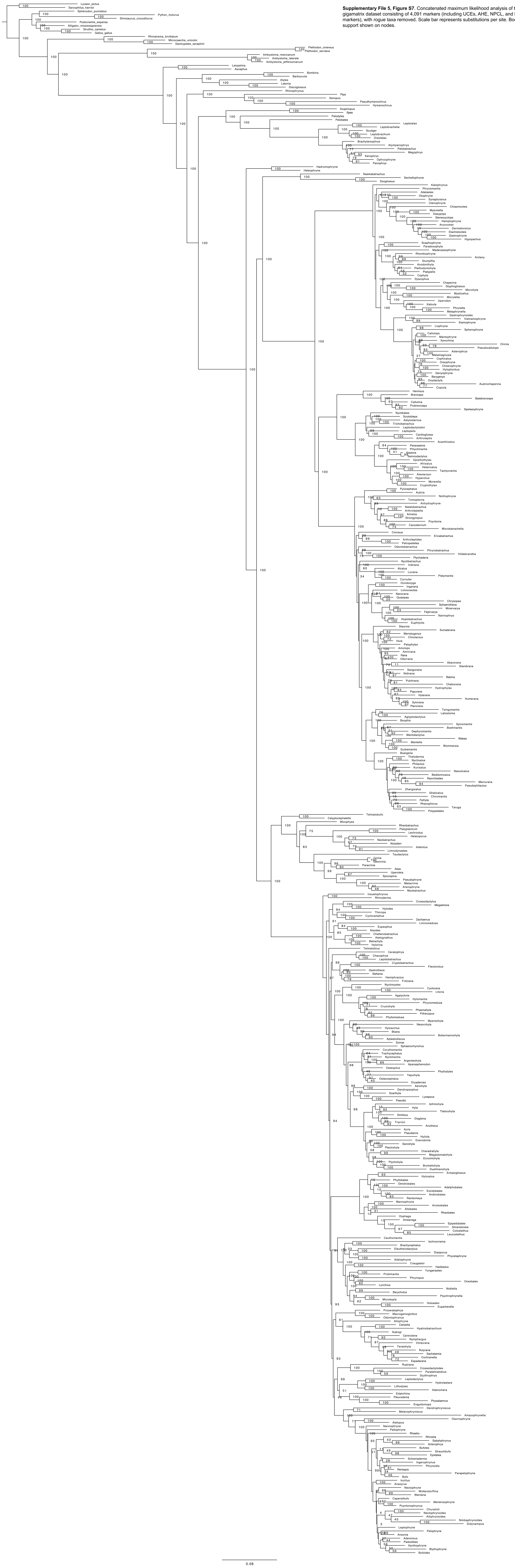

Supplementary File 5, Figure S7. Concatenated maximum likelihood analysis of the gigamatrix dataset consisting of 4,091 markers (including UCEs, AHE, NPCL, and legacy markers), with rogue taxa removed. Scale bar represents substitutions per site. Bootstrap support shown on nodes.

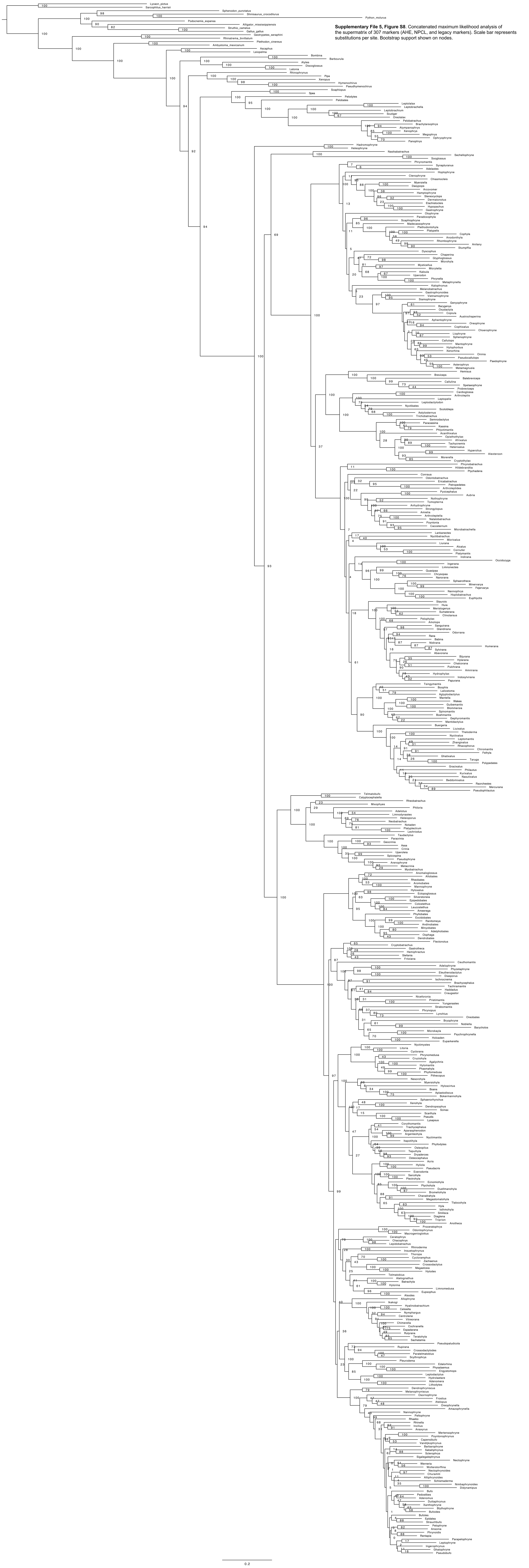

Supplementary File 5, Figure S8. Concatenated maximum likelihood analysis of the supermatrix of 307 markers (AHE, NPCL, and legacy markers). Scale bar represents substitutions per site. Bootstrap support shown on nodes.
