# Supplementary figures and images for: Redefining Possible: Combining Phylogenomic and Supersparse Data in Frogs
Source: Mol Biol Evol. 2023 May 4;40(5):msad109. doi: 10.1093/molbev/msad109 (PMC10202597; doi:10.1093/molbev/msad109)

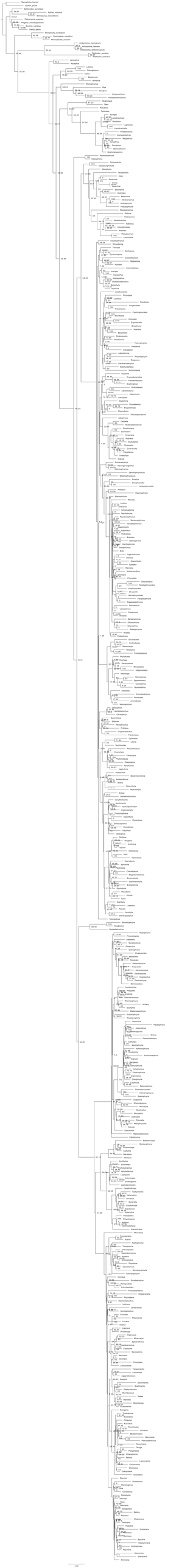

Supplement: msad109_Supplementary_Data [file msad109_supplementary_data.zip › Supplementary File S7.pdf]

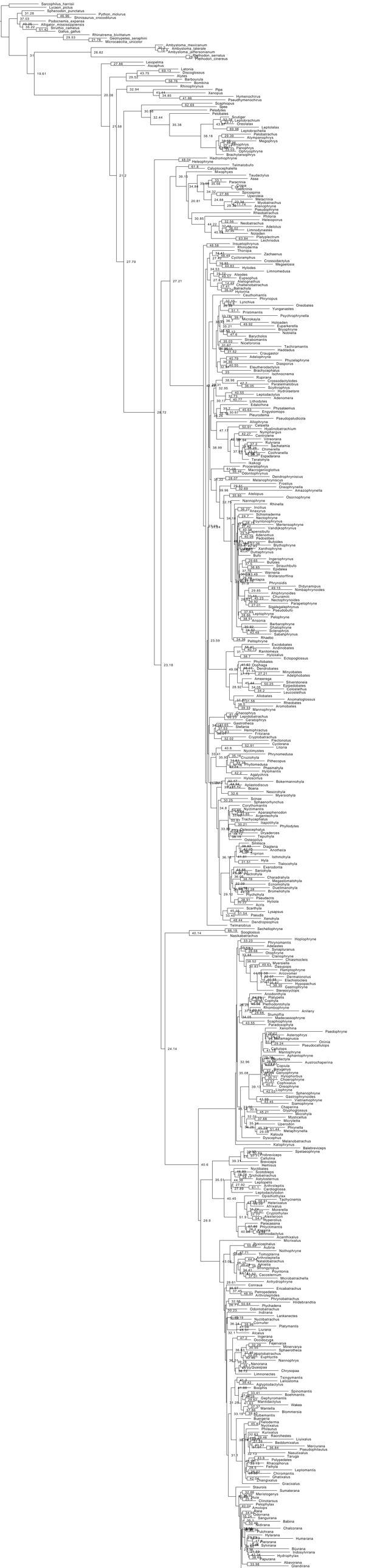

Supplement: msad109_Supplementary_Data [file msad109_supplementary_data.zip › Supplementary File S8.pdf]

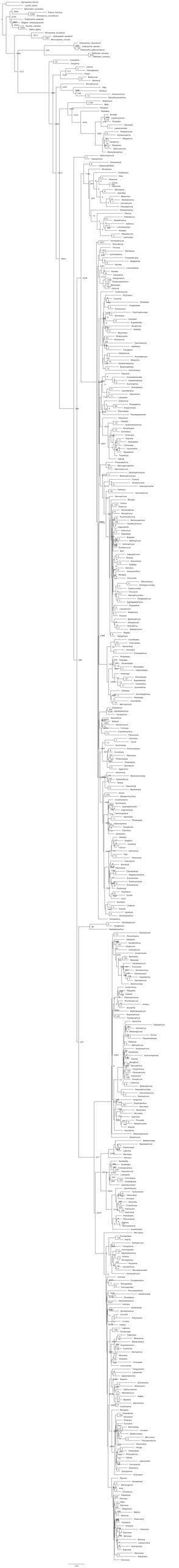

Supplement: msad109_Supplementary_Data [file msad109_supplementary_data.zip › Supplementary File S9.pdf]

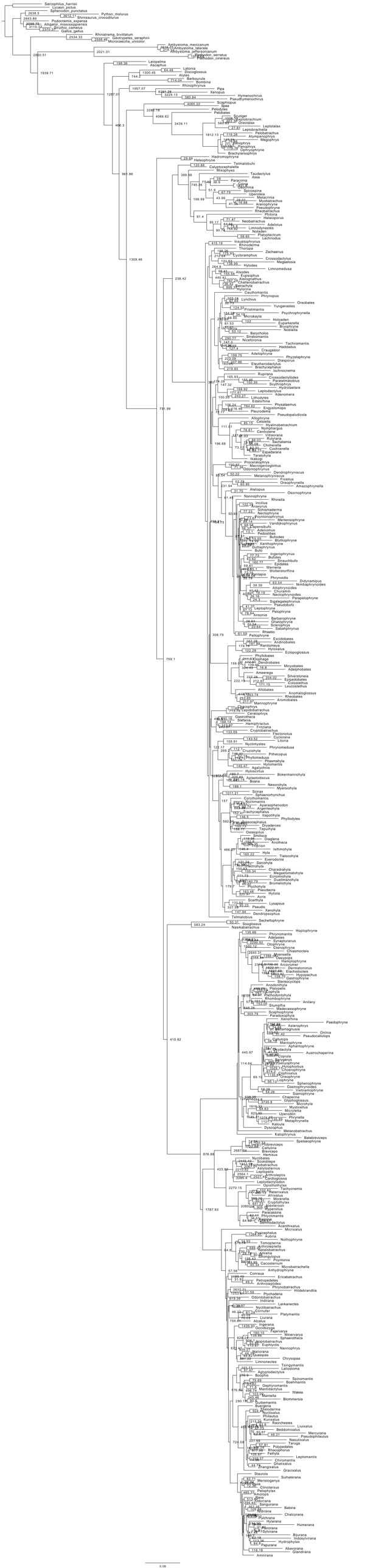

Supplement: msad109_Supplementary_Data [file msad109_supplementary_data.zip › Supplementary File S10.pdf]

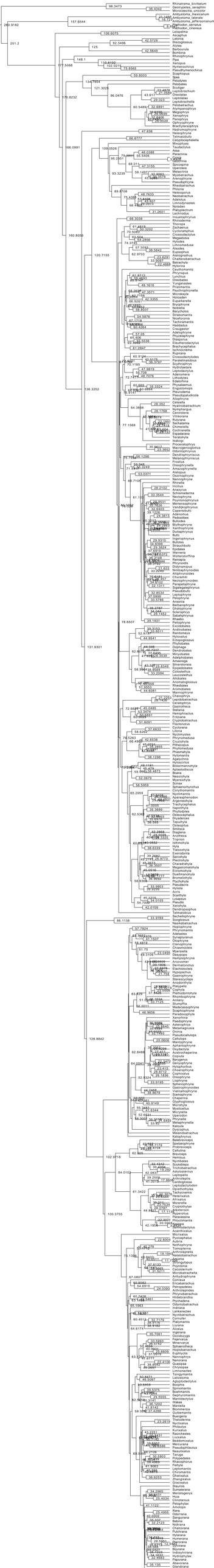

Supplement: msad109_Supplementary_Data [file msad109_supplementary_data.zip › Supplementary File S11.pdf]

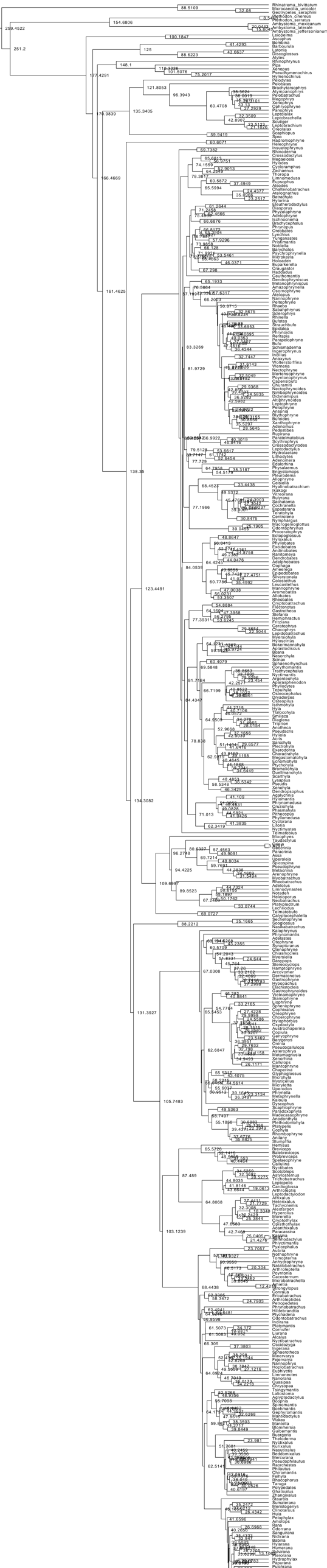

Supplement: msad109_Supplementary_Data [file msad109_supplementary_data.zip › Supplementary File S12.pdf]
